# Investigating the impact of Sprouty-related EVH1 domain-containing protein 3 on gastric cancer prognosis and tumor microenvironments

**DOI:** 10.3389/fonc.2026.1791690

**Published:** 2026-08-21

**Authors:** Shaowei Sun, Jiangtao Yu, Xiangyun Zheng, Huanhu Zhang

**Affiliations:** Department of Colorectoanorectal Surgery, Weihai Municipal Hospital, Weihai, China

**Keywords:** gastric cancer, immune, prognosis, progression, SPRED3

## Abstract

**Introduction:**

Gastric cancer is a prevalent and lethal malignancy, yet effective biomarkers remain limited. SPRED3 has been implicated in other cancers, but its role in gastric cancer has remained completely unexplored.

**Methods:**

SPRED3 expression and clinical significance were analyzed using TCGA and GEO datasets. Knockdown experiments were performed in AGS and N87 gastric cancer cell lines to assess effects on proliferation (CCK-8), migration, and invasion (Transwell). Pathway changes were examined by Western blot and qRT-PCR. Immune infiltration was evaluated via CIBERSORT and TIDE algorithms, and drug sensitivity was predicted using GDSC database, with CETSA validating alpelisib-SPRED3 binding.

**Results:**

SPRED3 was significantly upregulated in gastric cancer tissues and cells, demonstrating diagnostic value (AUC = 0.794) and a significant association with poor prognosis. Knockdown of SPRED3 inhibited cell proliferation, migration, and invasion, and suppressed the MAPK and NF-κB pathways. SPRED3 expression correlated positively with M2 macrophage infiltration and immune checkpoints (PD-L1, CTLA-4), and negatively with CD8+ T cells and activated NK cells. High SPRED3 expression was associated with elevated tumor immune dysfunction and exclusion scores, indicating immune exclusion and poor immunotherapy response, whereas low SPRED3 expression predicted increased sensitivity to alpelisib.

**Discussion:**

Our results indicate that SPRED3 serves as a prognostic and immunological biomarker in gastric cancer. Conceptually, this study introduces SPRED3 as a previously unrecognized regulator in gastric cancer pathogenesis and immunity.

## 1 Introduction

Gastric cancer is one of the most common malignant tumors around the world, representing a considerable risk to public health. According to global cancer statistics from 2022, there were over 968, 000 new cases of gastric cancer and approximately 660, 000 deaths, making it the fifth most frequently occurring cancer and the fourth leading cause of cancer-related deaths internationally (1). Despite significant advancements in postoperative adjuvant chemotherapy, neoadjuvant chemotherapy, and perioperative chemotherapy, numerous challenges persist in its management (2). The early stages of gastric cancer are often asymptomatic, leading to poor specificity and sensitivity of clinical disease markers; consequently, most patients are diagnosed at advanced stages (3). Studies indicate that over 60% of patients present with local or distant metastasis at diagnosis, with 5-year survival rates of 30% and 5%, respectively (3). Therefore, there is an urgent need for new diagnostic or screening methods for gastric cancer to facilitate early diagnosis and improve the prognosis for affected individuals.

Sprouty-related EVH1 domain containing 3 (*SPRED3*), also known as Eve-3 or spred-3, is a member of the Sprouty-related protein family. It features an N-terminal EVH-1 domain and a C-terminal SRY domain (4, 5). SPRED3 primarily influences cell proliferation, differentiation, and migration by regulating the MAPK signaling pathway (6). Research indicates that abnormal expression of SPRED3 is closely associated with the occurrence and progression of various tumors. For example, in thyroid cancer (THCA), *SPRED3* expression levels are significantly elevated and can be used to predict the progression of the disease and the prognosis of patients with THCA (6). Furthermore, THCA patients exhibiting high *SPRED3* expression demonstrate an increased mutation rate, particularly in the *BRAF* gene (6). Functional studies have shown that *SPRED3* promotes the proliferation of THCA cells through regulating the NF-κB signaling pathway (6). Regarding the immune microenvironment, elevated *SPRED3* expression is closely linked to immune cell infiltration (eosinophils, neutrophils, and macrophages), within the tumor microenvironment (TME) (7). Higher *SPRED3* expression is associated with poor prognosis in head and neck squamous cell carcinoma (HNSCC) (8). Additionally, *SPRED3* exhibits significant biological functions in other conditions. For example, in idiopathic hypogonadotropic hypogonadism (IHH), *SPRED3* has been identified as a novel candidate gene, with its variation potentially linked to the genetic background of the disease (9). Recombination of *Spred3* has been shown to exacerbate the BPD phenotype and related pulmonary arterial hypertension (PAH) (10). Accordingly, *SPRED3* plays a crucial role in various diseases. Its oncogenic properties in tumors and its influence on the immune microenvironment position it as a potential prognostic biomarker and therapeutic target.

Despite growing evidence implicating *SPRED3* in other cancers, its role in gastric cancer, particularly regarding prognosis, tumor progression, and immune modulation, remains completely unknown. The central biological question of this study is whether *SPRED3* serves as a novel prognostic and immune-related biomarker in gastric cancer. To address this question, we integrated multi-omics data analysis, *in vitro* functional experiments, and immune infiltration characterization to systematically evaluate the clinical significance and biological functions of *SPRED3* in gastric cancer.

## 2 Materials and methods

### 2.1 Data acquisition

The RNA sequencing data along with the relevant clinical information for gastric cancer were sourced from The Cancer Genome Atlas (TCGA, https://tcga-data.nci.nih.gov/tcga/), which encompasses 350 samples of gastric cancer alongside 32 adjacent normal samples (11). Furthermore, datasets GSE65801 (12) and GSE15459 (13) were retrieved from the Gene Expression Omnibus (GEO) database (http://www.ncbi.nlm.nih.gov/geo/). The independent groups in the GSE65801 dataset consisted of 32 gastric cancer samples and 32 normal specimens, whereas the GSE15459 dataset featured 192 gastric cancer cases complete with clinical details.

### 2.2 Gene expression analysis

*SPRED3* expression was evaluated between gastric cancer and normal samples using differential gene expression analysis from the TCGA cohort and the GSE65801 dataset. Differential gene expression analysis was performed using the R package “DESeq2” (14). *SPRED3* expression was considered to have significant differences between the two groups when |log2 Fold Change| > 1 and FDR < 0.05. Moreover, the expression differences of *SPRED3* between different pathological stages, ages and genders of gastric cancer were also performed.

### 2.3 Survival and receiver operating characteristics analysis

In the TCGA cohort and the GSE15459 dataset, gastric cancer patients were categorized into *SPRED3*^high^ and *SPRED3*^low^ groups, determined by the median expression value of *SPRED3*. To estimate the overall survival rates for these classifications, the Kaplan-Meier method was applied, utilizing the “survival” and “survminer” packages within R (15). Differences in survival rates among the groups were evaluated with the log-rank test to ascertain their significance. In addition, the R package “pROC” was employed to construct the ROC curve and compute the area under the curve (AUC) (16). The relationship between *SPRED3* expression levels and the prognosis of gastric cancer patients was analyzed through both univariate (17) and multivariate Cox regression approaches. Following this analysis, a nomogram model was created based on these variables, making use of the “rms” package.

### 2.4 Functional enrichment analysis

Gene Ontology (GO) enrichment analysis, including Biological Process, Molecular Function, and Cellular Component, along with Kyoto Encyclopedia of Genes and Genomes (KEGG) pathway analysis (18), was performed using the clusterProfiler package in R (v4.8.3) (19). Significantly enriched GO terms and KEGG pathways were identified with a threshold of P-value < 0.05.

Gene Set Enrichment Analysis (GSEA) was conducted between groups using the clusterProfiler package (v4.8.3) (19). Pathways with p.adjust < 0.05 were considered significantly enriched. Gene Set Variation Analysis (GSVA) was performed using the R package GSVA with the gene set “c2.all.v2024.1.Hs.symbols” from the MSigDB database. For each sample, an enrichment score was calculated based on the expression distribution of genes within each gene set. Positive scores indicate pathway activation, while negative scores indicate suppression. This approach transformed the gene expression matrix into a pathway activity matrix, enabling subsequent identification of differentially activated pathways between groups using standard statistical methods.

### 2.5 Cell culture and transfection

Human gastric mucosal epithelial cell line GES-1 (BNCC337969), human gastric cancer cell lines N87 (BNCC341304) and MKN45 (BNCC337682), and human monocyte THP-1 (BNCC358410) were purchased from BNCC (China). Human gastric cancer cell line AGS (CL-0022) was obtained from Procell Life Science & Technology Co., Ltd. (Wuhan, China). The N87, MKN45, and GES-1 cells were cultured in RPMI-1640 medium (Gbico, C11875500BT), while AGS cells were maintained in F-12K culture medium (Procell, PM150810). The culture media routinely contained 10% FBS (Solarbio, P1020) and 1% PS (Biosharp, BL505A). THP-1 cells were cultured in THP-1 cell-specific medium (BNCC354257, BNCC, China) at 37 °C in 5% CO_2_ and 95% air. Macrophages (M0) were generated by exposing THP-1 cells to 100 ng/mL Phorbol 12-myristate 13-acetate (PMA) (HY-18739, MCE) for 24 h. All cell lines used in this study were authenticated by an expert before the experiments. All cell lines were confirmed to be free of mycoplasma contamination.

*SPRED3*-siRNAs (si-*SPRED3*) and control siRNA (si-NC) were obtained from Tsingke Biotechnology Co., Ltd (Beijing, China) and used for transfection into AGS and N87 cell lines (**Table 1**). Cell transfection was conducted using Trans Exp transfection reagent (Gencefe, TRS001) (20). Subsequently, RNA was extracted 48 hours post-transfection to assess the mRNA and protein expression levels of *SPRED3*. Functional experiments were also performed following transfection.

**Table 1:** Knockdown siRNA sequence of SPRED3 gene.

Gene	Knockdown siRNA sequence
Si-*SPRED3*-1	Sense- GUUUCGAGGAGCUGGAAGU
	Antisense-ACUUCCAGCUCCUCGAAAC
Si-*SPRED3*-2	Sense-CAUCCGGAACUUGGAGCUU
	Antisense-AAGCUCCAAGUUCCGGAUG
Si-*SPRED3*-3	Sense-GCUCGGAUAUACUGAAACA
	Antisense-UGUUUCAGUAUAUCCGAGC
Si-NC	Sense-UUCUCCGAACGUGUCACGUTT
	Antisense-ACGUGACACGUUCGGAGAATT

### 2.6 Co‐culture system

Conditioned media (CM) were collected from AGS cells transfected with si-NC or si-*SPRED3*. The supernatants were centrifuged at 1, 500×g for 10 min at 4 °C to remove cell debris to obtain AGS-conditioned medium (AGS-CM). Next, M0 macrophages were treated with freshly prepared AGS-CM for 24 h. The experimental groups were as follows (1): M0 control (2), M0 + AGS-siNC-CM (, and (3) M0 + AGS-si*SPRED*3-CM.

### 2.7 Quantitative reverse-transcription polymerase chain reaction

RNA extraction from tissues and cells was performed utilizing TRNzol Universal Reagent (Thermo Fisher Scientific, 15596018CN), following the guidelines provided by the manufacturer. The extracted RNA was reverse transcribed using the M-MLV RT Premix (Accurate Biology, AG11706). Subsequently, reverse transcription quantitative PCR (RT-qPCR) was performed on a Veriti PCR system (Thermo Fisher Scientific) using SYBR Green Premix (Accurate Biology, AG11739). The expression levels of target genes were assessed through the 2^-△△Ct^ technique (21), utilizing *ACTB* as the reference gene. Comprehensive details about the primer sequences can be found in **Table 2**.

**Table 2:** Primer sequences for qRT-PCR.

Genes	Forward primer (5’-3’)	Reverse primer (5’-3’)
*ACTB*	GCTCACCATGGATGATGATATCGC	TAGGAATCCTTCTGACCCATGCC
*SPRED3*	CCAGGGGCACTACGTCATC	AACGTCAGTCCAAACTTGCAG
*CCL2*	CAGCCAGATGCAATCAATGCC	TGGAATCCTGAACCCACTTCT
*CCL5*	CCAGCAGTCGTCTTTGTCAC	CTCTGGGTTGGCACACACTT
*iNOS*	TCCGAGGCAAACAGCACATTCA	GGGTTGGGGGTGTGGTGATGT
*IL-6*	ACTCACCTCTTCAGAACGAATTG	CCATCTTTGGAAGGTTCAGGTTG
*ARG1*	GTGGAAACTTGCATGGACAAC	AATCCTGGCACATCGGGAATC
*IL-10*	GACTTTAAGGGTTACCTGGGTTG	TCACATGCGCCTTGATGTCTG
*CD206*	TCTTTGCCTTTCCCAGTCTCC	TGACACCCAGCGGAATTTC
*IL-8*	TTTTGCCAAGGAGTGCTAAAGA	AACCCTCTGCACCCAGTTTTC
*TNF-α*	CTGAACTTCGGGGTGATCGG	GGCTTGTCACTCGAATTTTGAGA
*CSF1R*	GGGAATCCCAGTGATAGAGCC	TTGGAAGGTAGCGTTGTTGGT

### 2.8 Western blot assay

Cell lysis was carried out on ice using RIPA lysis buffer (Beyotime Biotechnology, P0013B) supplemented with PMSF protease inhibitor for a duration of 15 minutes (22). The lysates were then subjected to centrifugation at 12, 000 rpm for 10 minutes at 4 °C, after which the supernatant was collected for total protein analysis. The quantification of proteins was performed using a BCA Protein Assay Kit (BIOSHARP, BL521A). Following this, SDS-PAGE was utilized to separate the proteins, and they were subsequently transferred onto PVDF membranes (Millipore, IPVH00010). After the transfer step, the membranes were blocked with 5% nonfat dry milk for 1 hour, and then incubated overnight at 4 °C with a primary antibody. The membranes were washed with 1x TBST and subsequently incubated for 1 hour with a secondary antibody. The primary antibodies used in this study were as follows: anti-SPRED3 polyclonal antibody (Thermo Fisher Scientific, PA5-20626, 1:300), anti-phospho-p38 (Proteintech, 28796-1-AP, 1:1000), anti-p38 (Proteintech, 14064-1-AP, 1:1000), anti-phospho-NF-κB p65 (Proteintech, 82335-1-RR, 1:2000), anti-NF-κB p65 (Proteintech, 80979-1-RR, 1:5000), anti-phospho-MKK3/6 (Abcam, ab289976, 1:1000), anti-MKK3/6 (Abcam, ab200831, 1:1000), anti-IκBα (Proteintech, 10268-1-AP, 1:5000), anti-phospho-IκBα (Proteintech, 82349-1-RR, 1:5000), and anti-GAPDH (Proteintech, 60004-1-Ig, 1:50, 000). The secondary antibodies used were Goat Anti-Rabbit IgG (ZSGB-Bio, ZB-2301, 1:5000) and Goat Anti-Mouse IgG (ZSGB-Bio, ZB-2305, 1:5000). Protein bands were detected using an ECL reagent (BIOSHARP, BL520B) on an Amersham Imager 600 instrument (GE Healthcare Bio-Sciences AB, Danderyd, Sweden). The primary images of the blots were shown in **Supplementary Figure S1**.

### 2.9 Cell viability assay

The impact of *SPRED3* knockdown on the viability of cells was assessed through the use of the Cell Counting Kit-8 (CCK-8) assay. AGS cells (5 × 10³ cells/well) transfected with si-NC and si-SPRED3 were seeded into 96-well plates and incubated at 37 °C for 24, 48, and 72 hours, respectively. At each time point, the cells were treated with CCK-8 solution (Biosharp, BS350A) for 2 hours at 37 °C. Finally, cell viability was assessed by measuring the absorbance at 450 nm using an enzyme-labeled instrument (Beijing Pulang Technology Co., Ltd, DNM-9602).

### 2.10 Migration and invasion assay

In this research, we utilized the Transwell assay to evaluate the migratory and invasive potential of the cells. We introduced 200 μL of a cell suspension at a concentration of 2.5×10^4^/mL into the upper chamber, which was either treated with Matrigel (Corning, 356234) or left untreated, whereas the lower chamber was filled with medium enriched with serum. The incubation period lasted for 48 hours. Following this, the medium from the upper chamber was discarded, and the chamber underwent two washes with PBS. Next, the cells were subjected to staining with crystal violet (Solarbio, G1070), and images were obtained using a fluorescence inverted microscope (CKX53-LP, OLYMPUS).

### 2.11 Enzyme linked immunosorbent assay

Cell culture supernatants were centrifuged at 1000×g for 15 min to remove particulates and were either assayed immediately or aliquoted and stored at ≤−20 °C until use. Repeated freeze-thaw cycles were avoided. The concentrations of monocyte chemoattractant protein-1 (MCP-1/CCL2) and regulated on activation, normal T cell expressed and secreted (RANTES/CCL5) in the supernatants were measured using commercial ELISA kits (Shanghai Enzyme-linked Biology, China; MCP-1: ml105321; RANTES: ml061100), following the manufacturer’s instructions. The optical density (OD) was read at 450 nm using a microplate reader (Infinite F50, TECAN).

### 2.12 CETSA

AGS cells were lysed using cell lysis buffer, and the lysates were incubated with 5 μM Alpelisib (MCE, HY-15244) or DMSO, respectively. The lysates were then aliquoted and subjected to a temperature gradient (40 °C, 43 °C, 46 °C, 49 °C, 52 °C, 55 °C, 58 °C, 61 °C, 64 °C, 67 °C, and 70 °C) for 10 min at each temperature. After incubation, the samples were centrifuged at 20, 000×g for 10 min at 4 °C to separate soluble proteins from denatured precipitates. The supernatants were collected, and SPRED3 levels were quantitatively analyzed by Western blotting.

### 2.13 Immune characteristics analysis

In the TCGA cohort, the tool CIBERSORT (23) was utilized to determine the relative proportions of 22 different immune cell types in each of the cancer samples. To explore the connection between *SPRED3* expression and the infiltrating immune cells, Pearson correlation analysis was employed. The Immune, Stromal, and ESTIMATE Scores were evaluated in *SPRED3*^high^ and *SPRED3*^low^ groups using the ESTIMATE function package (24). Additionally, the Tumor Immune Dysfunction and Exclusion (TIDE) algorithm was applied to forecast tumor immune evasion and responses to immune checkpoint inhibitors. A Pearson correlation analysis was performed to examine the association between *SPRED3* expression and immune checkpoints.

Immunotherapy response was evaluated using the Immunophenoscore (IPS) algorithm provided by The Cancer Immunome Atlas (TCIA, https://tcia.at). Four distinct IPS categories were calculated based on the predicted response to immune checkpoint inhibitors: ips_ctla-4_neg_pd-1_neg, ips_ctla-4_neg_pd-1_pos, ips_ctla-4_pos_pd-1_neg, and ips_ctla-4_pos_pd-1_pos. Higher IPS values indicate greater immunogenicity and predicted responsiveness to immunotherapy.

### 2.14 Drug sensitivity analysis

The “oncopredict” (25) function package in the R language was utilized for predicting drug sensitivity. The training set consists of the Genomics of Drug Sensitivity in Cancer 2 (GDSC2)_Expr expression matrix, containing 17, 419 genes and 805 cell lines, in addition to the GDSC2_Res drug sensitivity data that provides IC_50_ values for 198 drugs across those same 805 cell lines. The expression matrix of the sample to be predicted is calculated using the calcPhenotype function, resulting in the IC_50_ value for the sample.

### 2.15 Statistical analysis

The Wilcoxon rank sum test was employed to compare differences in gene expression and immune cell infiltration between the various groups. A Pearson correlation analysis was carried out utilizing the R language, with a significance level set at *p* < 0.05. All statistical analyses were executed using R software version 4.3.3.

All experiments were performed in triplicate. Data are presented as mean ± SD. Statistical analysis was conducted using GraphPad Prism 9 (9.5.0 (730)). Two-tailed unpaired Student’s t-test was used for comparisons between two groups, and p < 0.05 was considered statistically significant.

## 3 Results

### 3.1 The expression characteristics of *SPRED3* in gastric cancer

**Figure 1:**
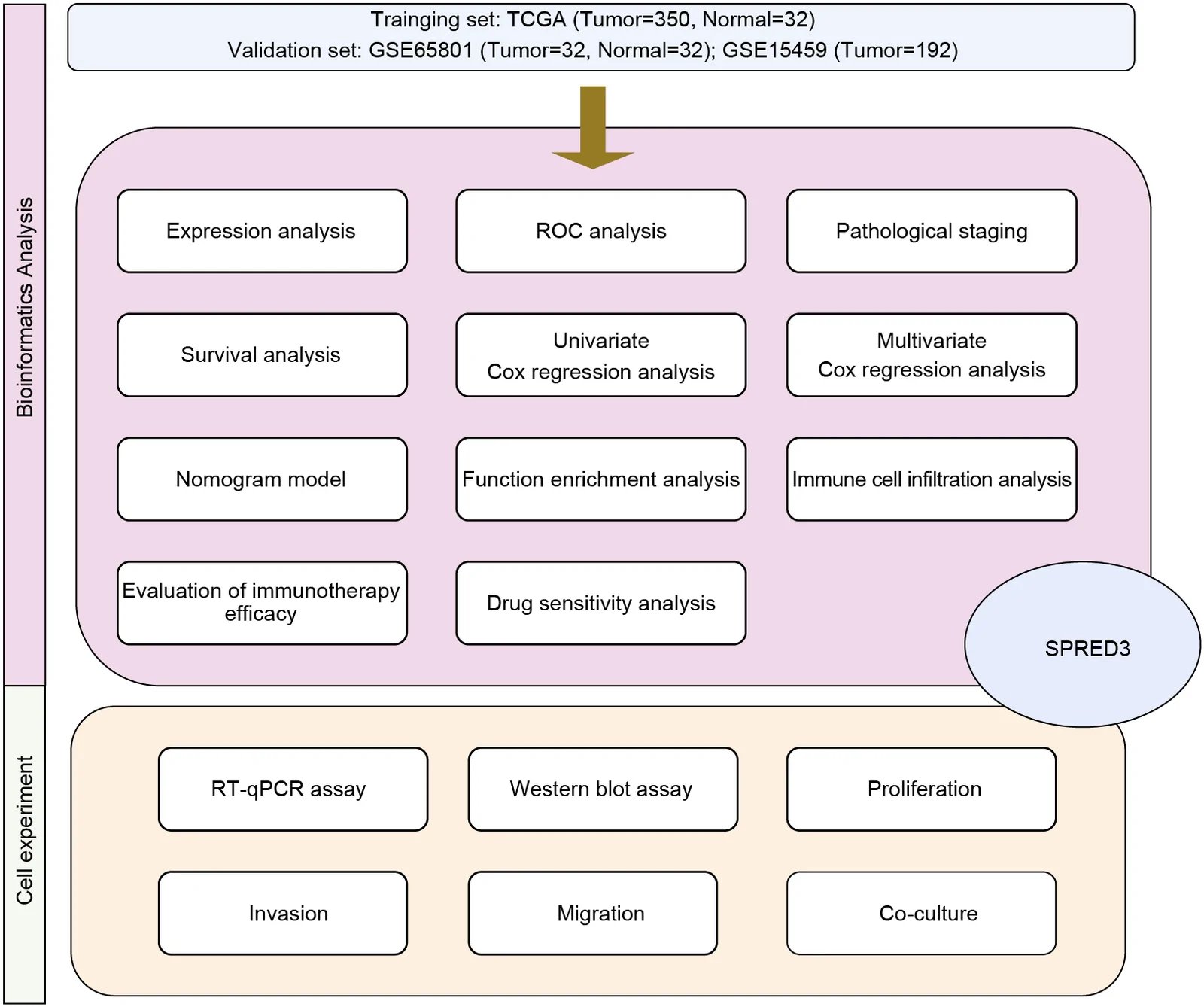
The flow diagram of this study.

**Figure 2:**
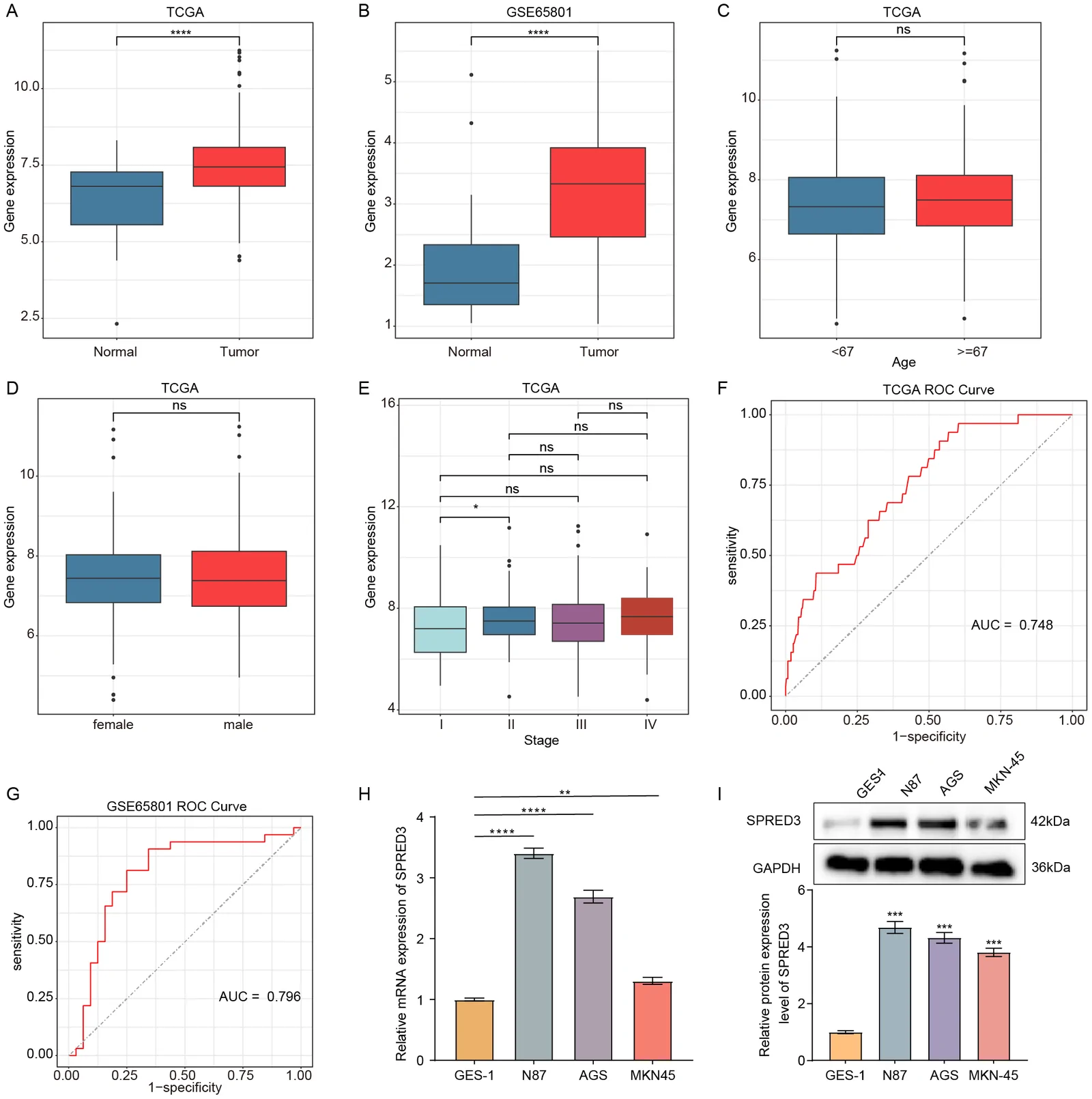
The expression characteristics of *SPRED3* in gastric cancer. **(A, B)** The expression of *SPRED3* in gastric cancer and normal samples in the TCGA **(A)** and GSE65801 cohorts **(B)**. C-E. Box plot comparing the expression level of *SPRED3* in different age **(C)** and sex **(D)**, and stage **(E)** in gastric cancer patients. F-G. ROC curve of *SPRED3* in gastric cancer in the TCGA **(F)** and GSE65801 cohorts **(G)**. H-I. The expression levels of SPRED3 mRNA **(H)** and protein **(I)** in gastric cancer cells were determined by qRT-PCR and Western blot assay, respectively. ns means no significance; * means *p* < 0.05; ** means *p* < 0.01; *** means *p* < 0.001; **** means *p* < 0.0001; ns means not significant.

The flow diagram representing this study is illustrated in **Figure 1**. Initially, we examined the expression levels of *SPRED3* in gastric cancer samples obtained from the TCGA and GSE65801 cohorts. Our findings revealed that *SPRED3* was notably upregulated in tumor tissue compared to normal samples (**Figures 2A, B**). Following this, we investigated how *SPRED3* expression correlates with the clinicopathological characteristics of gastric cancer patients within the TCGA cohort. No significant variations in *SPRED3* expression were observed concerning age and sex (**Figures 2C, D**). Importantly, an increase in *SPRED3* expression was found in stage II compared to stage I among gastric cancer patients (**Figure 2E**). Additionally, we employed the ROC curve to evaluate *SPRED3*’s diagnostic potential in differentiating between gastric cancer and normal samples. The AUC values were recorded at 0.748 for the TCGA (**Figure 2F**) and 0.796 for the GSE65801 cohorts (**Figure 2G**), indicating a significant diagnostic capacity for *SPRED3* in gastric cancer patients. Lastly, we confirmed the expression levels of *SPRED3* mRNA and protein through qRT-PCR and Western blot analysis, which demonstrated a marked upregulation in N87, AGS, and MKN-45 cells compared to GES-1 cells (**Figures 2H, I**).

### 3.2 Prognosis analysis of *SPRED3* expression in gastric cancer

**Figure 3:**
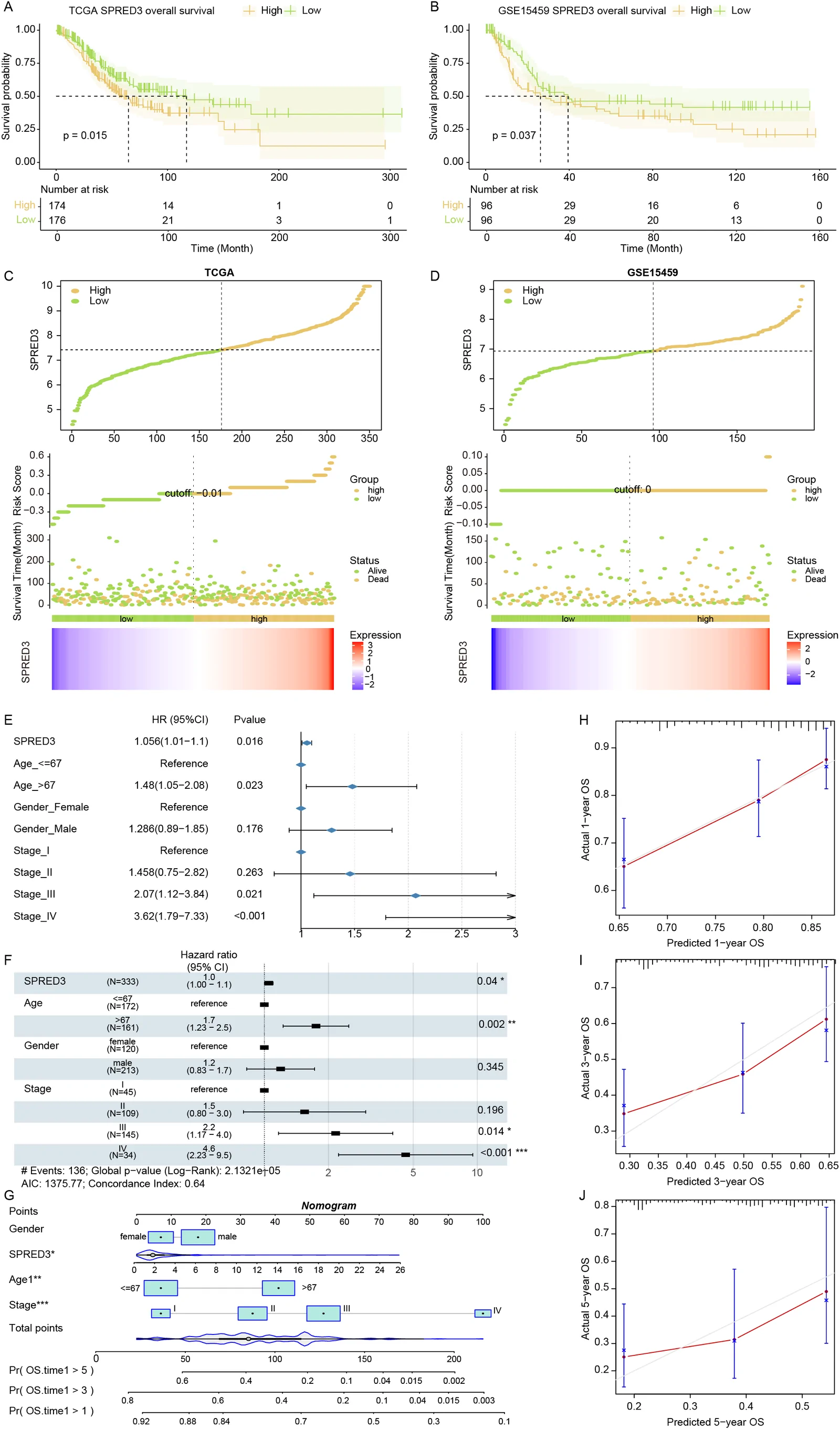
Prognosis analysis of *SPRED3* expression in gastric cancer. **(A, B)** Kaplan-Meier survival analysis for overall survival of gastric cancer with *SPRED3* high and low expression in the TCGA cohort **(A)** and GSE15459 dataset **(B)**. C-D. Distribution of adjusted *SPRED3* according to the survival status and time in TCGA **(C)**, GSE15459 **(D)** cohort. **(E)** The results of univariate Cox regression analysis. **(F)** The results of multivariate Cox regression analysis. **(G)** A nomogram was established to predict the prognostic of gastric cancer patients. H-J. Calibration plots showing the probability of 1- **(H)**, 3- **(I)**, and 5-year **(J)** overall survival in the TCGA cohort. * means *p* < 0.05; ** means *p* < 0.01; *** means *p* < 0.001.

To explore the influence of *SPRED3* on the prognosis of patients with gastric cancer, we conducted a Kaplan-Meier survival analysis for overall survival utilizing data collected from the TCGA and GSE15459 cohorts. The results indicated that gastric cancer patients with high *SPRED3* expression exhibited shorter OS compared to those with low *SPRED3* expression (**Figures 3A, B**). In both the TCGA and GSE15459 cohorts, patients in the high *SPRED3* expression group had a shorter survival time than those in the low *SPRED3* expression group (**Figures 3C, D**). Additionally, a univariate Cox regression analysis revealed that *SPRED3* expression, along with age, gender, and stage, was associated with the prognosis of gastric cancer patients in the TCGA cohort (**Figure 3E**). In addition, the results from the multivariate Cox regression analysis revealed that the expression of *SPRED3* acted as an independent prognostic marker within the TCGA cohort (**Figure 3F**). Lastly, we created a nomogram model for overall survival to estimate the survival status of patients at 1, 3, and 5 years (**Figure 3G**). The survival rates at 1 year and 3 years as predicted by the model closely matched the actual observed rates (**Figures 3H-J**), confirming the model’s effectiveness.

### 3.3 Influence of *SPRED3* knockdown on cell proliferation, invasion and migration in gastric cancer

**Figure 4:**
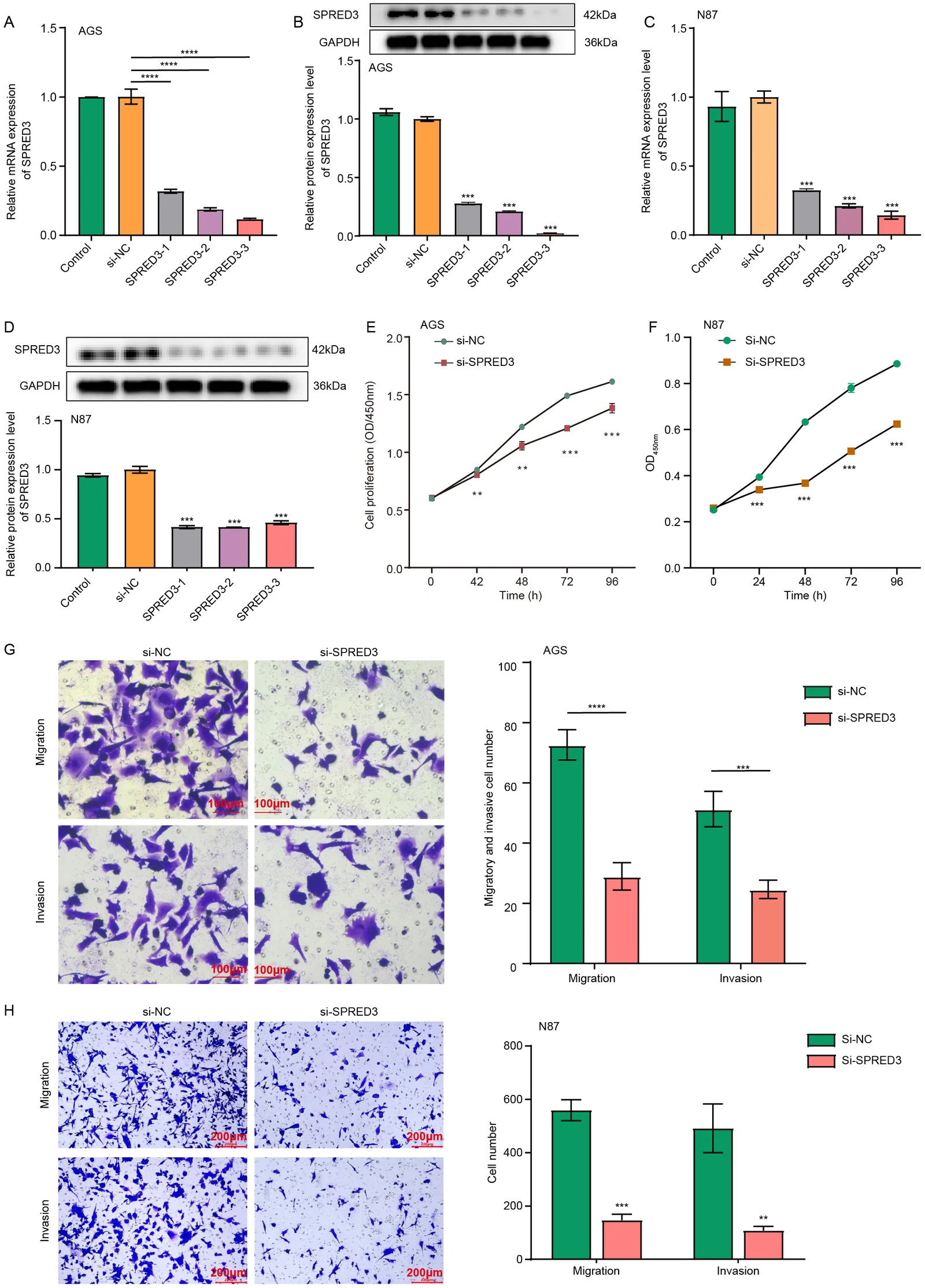
Influence of SPRED3 knockdown on cell proliferation, invasion and migration in gastric cancer. **(A-D)**. Knockdown efficiency of three independent siRNAs targeting *SPRED3* was validated in AGS **(A-B)** and N87 **(C, D)** cells. SPRED3 mRNA and protein levels were measured by qRT-PCR **(A, C)** and Western blot **(B, D)**, respectively. **(E, F)**. CCK-8 assay was performed to evaluate cell viability in AGS **(E)** and N87 **(F)** gastric cancer cells following *SPRED3* knockdown. **(G, H)**. Transwell assay was performed to evaluate the migration and invasion capabilities of AGS **(G)** and N87 **(H)** gastric cancer cells following *SPRED3* knockdown. Data are mean ± SD from three experiments. ** means *p* < 0.01; *** means *p* < 0.001; **** means *p* < 0.0001.

To investigate the effects of *SPRED3* knockdown on tumor cell phenotypes, AGS and N87 cells were transfected with *SPRED3*-specific siRNAs. As shown in **Figure 4A-D**, SPRED3 mRNA and protein levels were markedly reduced in the si-*SPRED3*-1, si-*SPRED3*-2, and si-*SPRED3*-3 groups compared to the negative control in both AGS (**Figure 4A, B**) and N87 (**Figure 4C, D**) cells. CCK-8 assays revealed that cell proliferation was markedly inhibited in *SPRED3*-knockdown cells compared to the si-NC group in both cell lines (**Figure 4E, F**). Furthermore, Transwell assays demonstrated that the migratory and invasive capacities of AGS and N87 cells were significantly reduced following *SPRED3* knockdown (**Figure 4G, H**). These findings indicate that *SPRED3* plays a critical role in regulating the proliferative, migratory, and invasive capacities of gastric cancer cells *in vitro*, suggesting its potential involvement in tumor progression.

### 3.4 Potential functional information of *SPRED3* in gastric cancer

**Figure 5:**
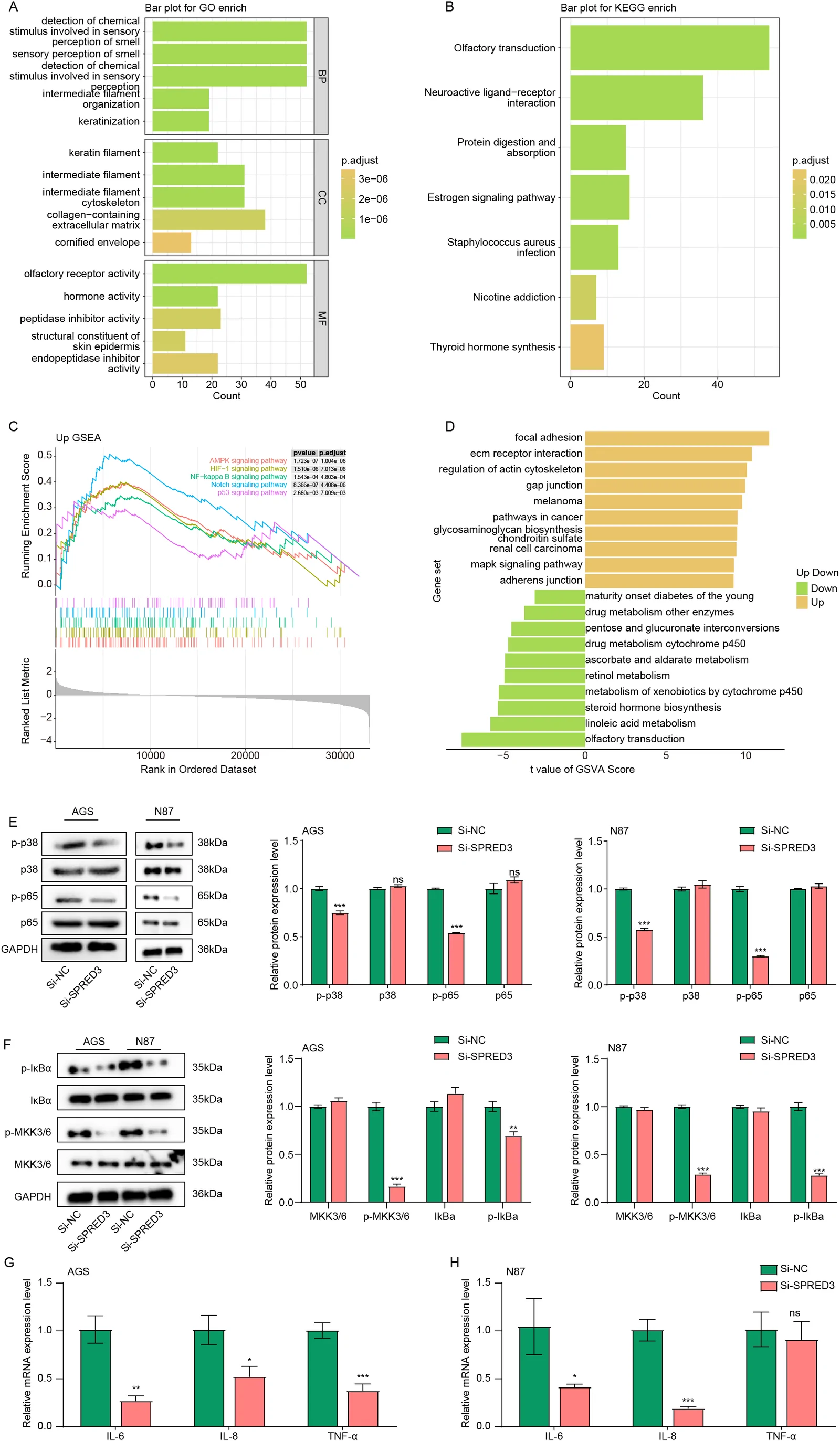
Potential functional information of *SPRED3* in gastric cancer. **(A)** The result of GO enrichment analysis. **(B)** The results of KEGG enrichment analysis. **(C)** The result of GSEA analysis. **(D)** The result of GSVA analysis. **(E)** Western blot analysis of total and phosphorylated p38 and p65 in AGS and N87 gastric cancer cells after *SPRED3* knockdown (Data are mean ± SD from three experiments). **(F)** Western blot analysis of total and phosphorylated MKK3/6 and IκBα in AGS and N87 gastric cancer cells after *SPRED3* knockdown (Data are mean ± SD from three experiments). G-H. qRT-PCR analysis of NF-κB downstream target gene expression in AGS **(G)** and N87 **(H)** gastric cancer cells following SPRED3 knockdown (Data are mean ± SD from three experiments). * means *p* < 0.05, ** means *p* < 0.01, *** means *p* < 0.001, ns means no significant difference.

In the TCGA cohort, gastric cancer patients were categorized into *SPRED3*^high^ and *SPRED3*^low^ groups, determined by the median expression value of *SPRED3* to identify DEGs. Furthermore, GO and KEGG pathway enrichment analyses were conducted based on these DEGs. The GO enrichment analysis revealed that these DEGs were significantly enriched detection of chemical stimulus involved in sensory perception of smell, sensory perception of smell, detection of chemical stimulus involved in sensory perception, and olfactory receptor activity (**Figure 5A**, **Supplementary Table S1**). The KEGG enrichment analysis indicated that these DEGs were primarily enriched in pathways such as olfactory transduction, neuroactive ligand-receptor interaction, and the estrogen signaling pathway (**Figure 5B**, **Supplementary Table S1**).

Furthermore, GSEA revealed that immune-related pathways, including AMPK, HIF-1, NF-κB, and Notch signaling, were significantly activated in the *SPRED3*^high^ group compared to *SPRED3*^low^ group (**Figure 5C**, **Supplementary Table S2**, p < 0.05). GSVA further indicated that patients with high *SPRED3* expression were mainly enriched in ECM-receptor interaction and the MAPK signaling pathway (**Figure 5D**, **Supplementary Table S3**, p < 0.05).

To experimentally validate these findings, we examined the effects of *SPRED3* knockdown on the predicted signaling pathways in both AGS and N87 gastric cancer cell lines. Western blot analysis revealed that *SPRED3* depletion significantly decreased the phosphorylation levels of p38, MKK3/6 (MAPK pathway), as well as p65 and IκBα (NF-κB pathway), without altering the total protein levels of these molecules in either cell line (**Figure 5E, F**). Furthermore, qRT-PCR analysis demonstrated that *SPRED3* knockdown markedly reduced the mRNA expression of the NF-κB downstream targets *IL-6*, *IL-8*, and *TNFα* in AGS cells (**Figure 5G**), and significantly decreased *IL-6* and IL-8 expression in N87 cells (**Figure 5H**). Collectively, these results confirm that *SPRED3* activates both the MAPK and NF-κB cascades across multiple gastric cancer cell models.

Consistently, correlation analysis revealed that *SPRED3* expression was significantly and positively correlated with key components of the HIF-1 (HIF1A, ARNT), Notch (NOTCH1, NOTCH2), MAPK (MAPK1, MAPK3), and NF-κB (NFKB1, NFKB2) pathways (**Supplementary Figure S2A-D**).

### 3.5 Immune characteristics analysis of *SPRED3* in gastric cancer

**Figure 6:**
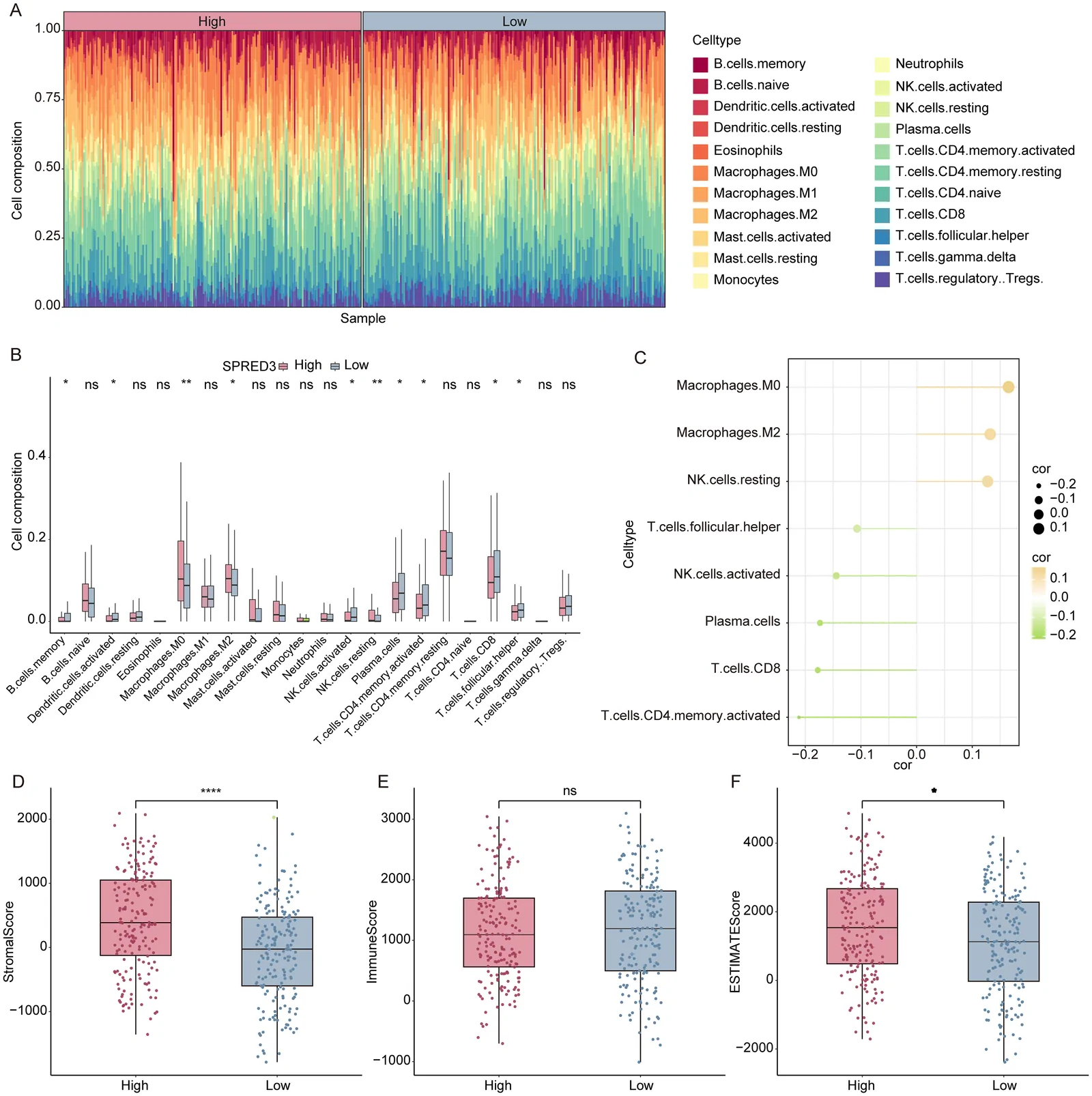
Immune characteristics analysis of SPRED3 in gastric cancer. **(A)** Heatmap of immune cell infiltration for each sample in the TCGA cohort. **(B)** The proportion of immune cell infiltration in the *SPRED3* high and low expression groups in the TCGA cohort. **(C)** The correlation between immune cell infiltration and *SPRED3* expression in the TCGA cohort. D-F. StromalScore **(D)**, ImmuneScore **(E)** and ESTIMATEScore **(F)** in the *SPRED3* high and low expression groups in the TCGA cohort. ns means no significance; * means *p* < 0.05; ** means *p* < 0.01; **** means *p* < 0.0001; ns means not significant.

In the TCGA cohort, the assessment of immune cell infiltration for each patient was carried out using the CIBERSORT algorithm (**Figure 6A**). There was a significant increase in the proportion of M0 macrophages, M2 macrophages, and resting natural killer (NK) cells, while the levels of memory B cells, activated dendritic cells, activated NK cells, plasma cells, activated memory CD4+ T cells, CD8+ T cells, and T follicular helper (Tfh) cells showed a marked decrease in the SPRED3^high^ group when compared to the SPRED3^low^ group (**Figure 6B**, p < 0.05). Additionally, further analysis indicated a positive correlation between *SPRED3* expression and the infiltration of M0 macrophages, M2 macrophages, and resting NK cells. Conversely, it was found to be negatively correlated with the infiltration of Tfh cells, activated NK cells, plasma cells, CD8+ T cells, and activated memory CD4+ T cells (**Figure 6C**). Moreover, we utilized the ESTIMATE algorithm to calculate the Stromal score, Immune score, and ESTIMATE score, which helped in assessing the distribution of immune cells and the extracellular matrix within TME. The results indicated that both the Stromal score and ESTIMATE score were significantly higher in the *SPRED3* high expression group compared to the *SPRED3* low expression group (**Figure 6D-F**).

Given the association between *SPRED3* and macrophages, we next investigated the mechanism by which *SPRED3* regulates macrophage polarization using a co-culture system. CM were collected from AGS cells transfected with si-NC or si-SPRED3, and the levels of CCL2 and CCL5 were measured by qRT-PCR and ELISA. As shown in **Supplementary Figures S3A, B**, knockdown of *SPRED3* significantly reduced CCL2 and CCL5 levels in the CM compared to the si-NC group. Subsequently, THP-1-derived M0 macrophages were co-cultured with CM from *SPRED3*-knockdown AGS cells. The expression of typical M0 phenotype marker (CSF1R), M1 phenotype markers (iNOS and IL-6), and M2 phenotype markers (ARG1, IL-10, and CD206) was assessed by qRT-PCR. Compared with the M0 + AGS-siNC-CM group, the M0 + AGS-si*SPRED3*-CM group exhibited significantly increased levels of CSF1R, iNOS and IL-6 (**Supplementary Figures S3C, D**), whereas the levels of ARG1, IL-10, and CD206 were markedly decreased (**Supplementary Figure S3E**). Furthermore, the migratory capacity of macrophages was also significantly reduced in the M0 + AGS-siSPRED3-CM group compared to the control group (**Supplementary Figure S3F**). These results support the hypothesis that *SPRED3* knockdown in AGS cells reduces M2 polarization and migration of macrophages.

### 3.6 Evaluation of immunotherapy efficacy of *SPRED3* in gastric cancer

**Figure 7:**
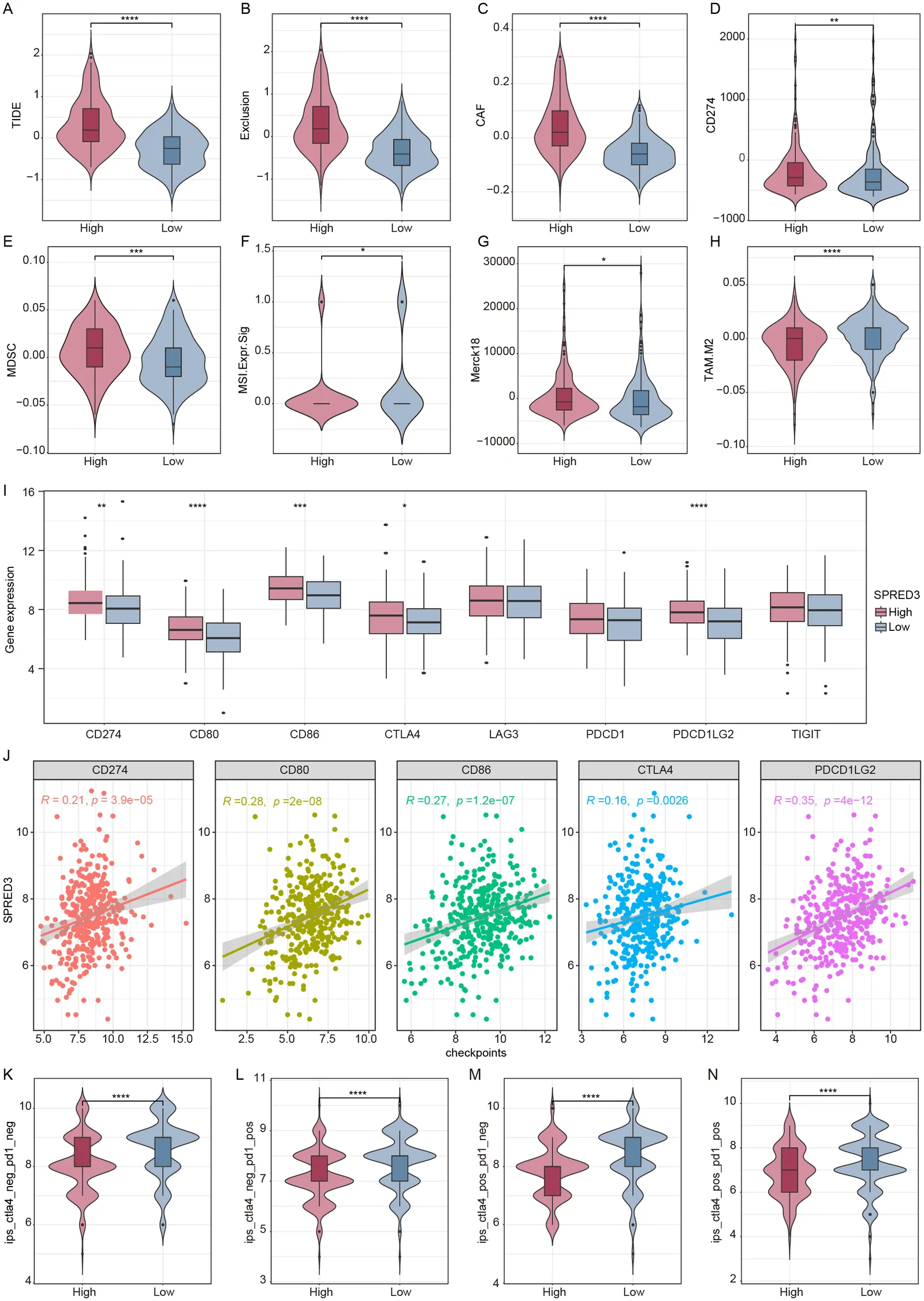
Evaluation of immunotherapy efficacy of *SPRED3* in gastric cancer. **(A–H)** The levels of TIDE score, immune exclusion score, CAF, CD274, MDSC, MSI, Merck 18, and TAM-M2 in the *SPRED3* high and low expression groups in the TCGA cohort. **(I)** The expression of immune checkpoint in the *SPRED3* high and low expression groups in the TCGA cohort. **(J)** The correlation between immune checkpoint expression and *SPRED3* expression in the TCGA cohort. **(K-N)** Immunotherapy efficacy scores according to four different scoring methods in the TCIA algorithm for gastric cancer patients with *SPRED3* high and low expression levels. * means *p* < 0.05; ** means *p* < 0.01; *** means *p* < 0.001; **** means *p* < 0.0001.

We next predicted tumor immune evasion and immune checkpoint inhibitor responses using Tumor Immune Dysfunction and Exclusion (TIDE) website. The result showed that gastric cancer patients with high *SPRED3* had higher TIDE score, immune exclusion score, CAF, CD274, MDSC, MSI and Merck 18 (high vs. low, *p* < 0.0001, **Figure 7A–G**) and exhibit low TAM-M2 (**Figure 7H**). Additionally, we examined the expression patterns of eight immune checkpoints in the *SPRED3*^high^ and *SPRED3*^low^ groups. Our findings indicated that the expressions of PD-L1 (CD274), CD80, CD86, CTLA4, and PD-L2 (PDCD1LG2) were markedly elevated in the *SPRED3*^high^ group compared to *SPRED3*^low^ group (**Figure 7I**). *SPRED3* expression was remarkably positively correlated with CD274, CD80, CD86, CTLA4, and PDCDILG2 expression in gastric cancer (**Figure 7J**). Furthermore, we found that the four different immunotherapy response scores (ips_ctla-4_neg_pd-1_neg, ips_ctla-4_neg_pd-1_pos, ips_ctla-4_pos_pd-1_neg, and ips_ctla-4_pos_pd-1_pos) were significantly increased in the *SPRED3*^low^ group compared to the *SPRED3*^high^ group (**Figure 7K–N**). Thus, our findings emphasize the crucial role of *SPRED3* in shaping therapeutic outcomes in gastric cancer.

### 3.7 Drug sensitivity analysis

**Figure 8:**
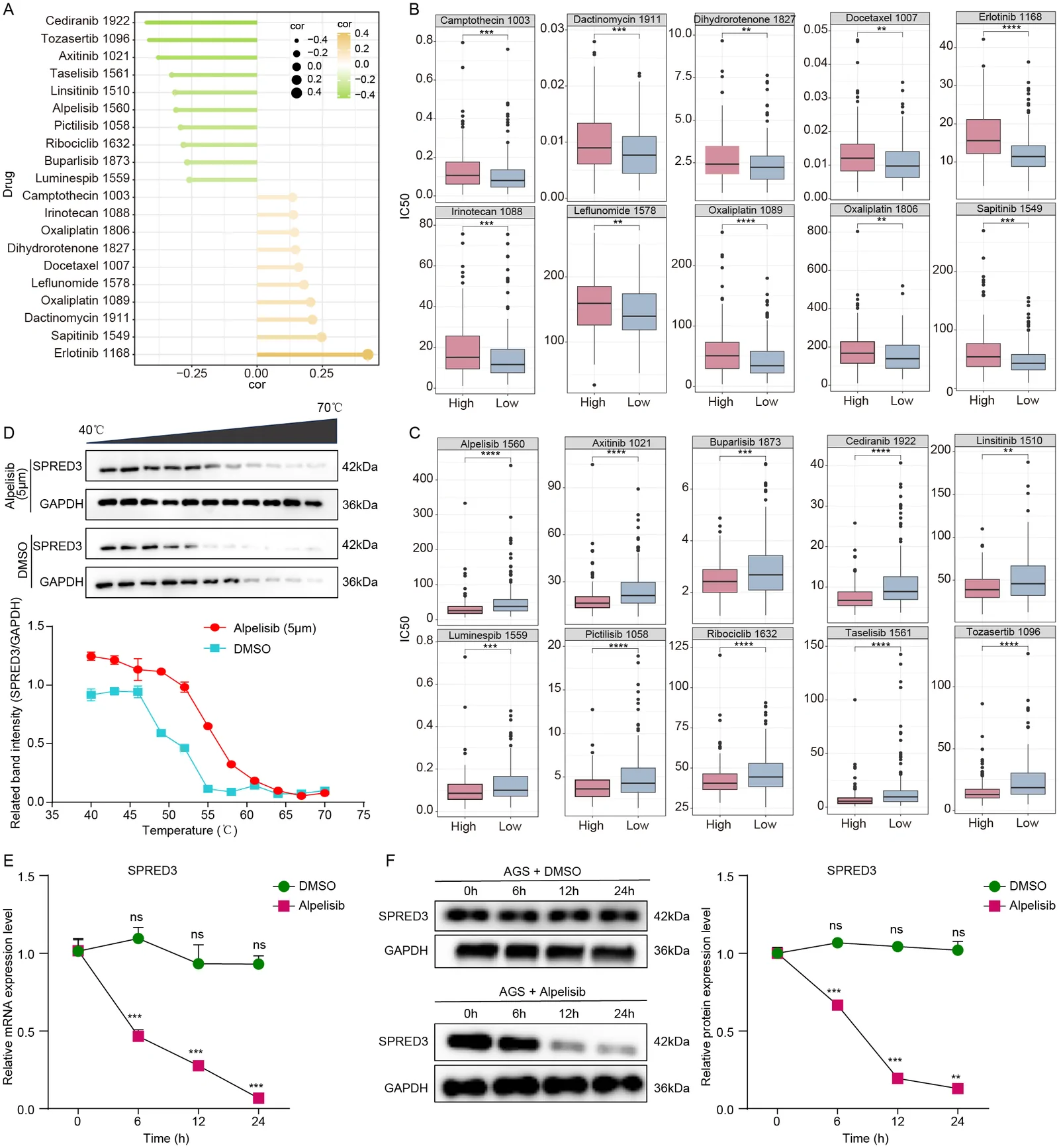
Drug sensitivity analysis. **(A)** Bubble plot of the relationship between drugs and *SPRED3* expression. **(B)** The correlation of the IC_50_ of drugs and *SPRED3* expression. **(D)** CETSA analysis of SPRED3 thermal stability in AGS cells treated with alpelisib or DMSO. E-F. AGS cells were treated with 5 µM alpelisib or DMSO for 0, 6, 12, and 24 h, and SPRED3 mRNA **(E)** and protein **(F)** levels were measured by qRT-PCR and Western blot, respectively. Data are mean ± SD from three experiments. ** means *p* < 0.01; *** means *p* < 0.001; **** means *p* < 0.0001.

To explore the relationship between the expression of SPRED3 and sensitivity to drugs, we performed a comprehensive sensitivity analysis encompassing 805 compounds sourced from the GDSC database. We subsequently evaluated the relationship between drug IC_50_ values and *SPRED3* expression levels, revealing a significant correlation between *SPRED3* expression and the IC_50_ values of 118 distinct drugs (**Figure 8A**, **Supplementary Table S4**). Notably, we observed that the IC_50_ values for Camptothecin_1003, Docetaxel_1007, Irinotecan_1088, Oxaliplatin_1089, Oxaliplatin_1806, and Sapitinib_1549 were elevated in the *SPRED3* high-expression group (**Figure 8B**), whereas the IC_50_ value for alpelisib_1560 was reduced (**Figure 8C**).

Given that our drug sensitivity prediction identified low SPRED3 expression as a correlate of increased sensitivity to alpelisib, a specific PI3Kα inhibitor, we next sought to determine whether this functional association might reflect a direct physical interaction between SPRED3 and alpelisib. To test this hypothesis, we performed CETSA, and the results demonstrated that alpelisib binding significantly enhanced the thermal stability of SPRED3 in AGS cells, as evidenced by a rightward shift of the melting curve between 40 °C and 70 °C compared to the control (**Figure 8D**). To determine whether alpelisib binding affects *SPRED3* expression, AGS cells were treated with 5 μM alpelisib for 0, 6, 12, and 24 h. Western blot and qRT-PCR analyses revealed that SPRED3 mRNA and protein levels remained constant over time in the DMSO-treated control group, whereas alpelisib treatment led to a time-dependent decrease in both SPRED3 mRNA and protein expression (**Figure 8E, F**). Collectively, these findings demonstrate that alpelisib binds to SPRED3 and is associated with a time-dependent reduction in its mRNA and protein levels, suggesting a potential regulatory feedback loop.

## 4 Discussion

In the current study, our findings indicate that *SPRED3* might serve as a potential biomarker for prognosis and immunotherapy in gastric cancer. We observed that *SPRED3* was upregulated in gastric cancer tissues and cells, and its elevated expression was correlated with poor patient outcomes. Moreover, our experimental data demonstrate that the knockdown of *SPRED3* significantly reduced the migratory and invasive capacities of gastric cancer cells. Further investigations reveal that *SPRED3* was not only associated with immune cell infiltration and immune checkpoint expression but was also linked to the response to immunotherapy. These findings indicate that *SPRED3* may play a multifaceted role in gastric cancer progression and immune regulation, highlighting its potential as a therapeutic target and prognostic indicator. Unlike previous studies that focused on *SPRED3* in thyroid or lung cancer (7, 26), this study is the first to systematically investigate its expression, prognostic value, biological functions, and immune correlations in gastric cancer, thereby addressing a previously unexplored area.

Studies investigating the expression profiles of *SPRED3* across various solid tumors have demonstrated elevated levels in THCA (6) and primary oral squamous cell carcinoma (8). In this research, we initially examined the molecular and biological features of *SPRED3* in gastric cancer. The expression of *SPRED3* was elevated in both gastric cancer tissues and cells, and the reduction of *SPRED3* levels led to a notable decrease in the proliferation, migration, and invasion of gastric cancer cells. Additionally, we found that increased *SPRED3* expression was associated with a worse prognosis in gastric cancer, which is consistent with findings in other types of cancer (7, 8).The expression of *SPRED3* demonstrates a significant correlation with histological grade and TNM stage in THCA patients (7). Notably, our study showed that the expression of *SPRED3* was significantly increased in stage II gastric cancer patients compared to stage I patients, suggesting that *SPRED3* may serve as an early indicator of gastric cancer. ROC analysis further supported the diagnostic potential of *SPRED3*, with an AUC value exceeding 0.7. This indicates that *SPRED3* has a relatively high discriminative power in distinguishing gastric cancer samples from normal samples. An AUC value greater than 0.7 suggests that *SPRED3* holds promise as a biomarker for the diagnosis of gastric cancer. These findings highlight the potential utility of *SPRED3* in clinical settings for early detection, risk stratification, and monitoring disease progression.

GO and KEGG enrichment analyses revealed that genes co-expressed with SPRED3 were predominantly enriched in olfactory transduction and neuroactive ligand-receptor interaction pathways. While these sensory-related pathways appear unconventional in the context of gastric cancer, they may reflect the limitations of transcriptome-wide enrichment approaches in capturing tumor-relevant biological processes. Alternatively, emerging evidence suggests that ectopic expression of olfactory receptors and neuroactive signaling molecules may play previously unrecognized roles in tumor biology (27–29). Nevertheless, these findings underscore the need for further experimental validation to elucidate the functional relevance of SPRED3 in gastric cancer.

*SPRED3* interacts with protein kinases via its EVH-1 domain, which in turn inhibits the activation of the Ras/MAPK signaling pathway (26). Furthermore, *SPRED3* enhances the proliferation of THCA cells by stimulating the NF-κB signaling pathway (6).This pathway is commonly associated with inflammation and tumor progression in various cancers (30, 31), and the elevated expression of *SPRED3* is closely linked to the activation of the NF-κB pathway (6). In NSCLC, the loss of *SPRED3* enhances the activation of the MAPK signaling pathway mediated by the EGFR, thereby promoting tumor progression (26). In the present study, the MAPK and NF-κB signaling pathways were significantly activated in gastric cancer patients with high *SPRED3* expression. Thus, we hypothesize that *SPRED3* may be involved in the progression of gastric cancer by modulating either the MAPK or the NF-κB signaling pathway. However, further research is required to elucidate the precise mechanisms through which *SPRED3* regulates the MAPK and NF-κB pathways in gastric cancer.

We acknowledge an apparent paradox between our findings and the aforementioned NSCLC study, where *SPRED3* loss enhanced MAPK activation, suggesting a beneficial role of SPRED3 in that context (26), whereas our study shows that *SPRED3* knockdown suppresses MAPK and NF-κB signaling and inhibits gastric cancer progression. This discrepancy likely reflects context-dependent functions of *SPRED3* across different cancer types. Notably, our functional enrichment and experimental validation indicate that both MAPK and NF-κB pathways are involved in *SPRED3*-mediated gastric cancer progression, with NF-κB signaling playing a prominent role (6). Therefore, the paradoxical observations may be explained by tissue-specific differences in pathway dependency and the relative contribution of *SPRED3* to each signaling axis. Further studies are warranted to dissect the mechanistic basis of this context dependency.

Gastric cancer remains a leading cause of cancer-related mortality worldwide, with high rates of recurrence and metastasis even after curative-intent surgery (2, 32–34). Immunotherapy has emerged as a promising therapeutic modality for gastric cancer, yet its efficacy varies considerably across patients (35–37). In the present study, we observed a positive correlation between SPRED3 expression and M2 macrophage infiltration in gastric cancer tissues. M2 macrophages are well-established immunosuppressive cells that promote tumor cell metastasis and angiogenesis while suppressing anti-tumor immune responses (38). This finding prompted us to investigate whether SPRED3 actively shapes the macrophage compartment within TME.

To address this question, we performed co-culture experiments and found that SPRED3 knockdown in gastric cancer cells significantly attenuated M2 macrophage polarization, accompanied by increased expression of M0 macrophage markers and impaired macrophage migration. Mechanistically, *SPRED3* knockdown markedly suppressed both the MAPK and NF-κB signaling pathways, as evidenced by reduced phosphorylation of MKK3/6, p38, IκBα, and p65, along with decreased expression of the downstream NF-κB target genes IL-6, IL-8, and TNFα. Given that IL-6 is a well-recognized inducer of M2-like polarization in TME (39), it is plausible that SPRED3 promotes M2 macrophage polarization, at least in part, through activating the MAPK/NF-κB signaling axis and subsequently upregulating this cytokine in tumor cells. This mechanistic finding is corroborated by a recent study demonstrating that *SPRED3* activates the NF-κB signaling pathway to promote proliferation in thyroid cancer (6), suggesting that *SPRED3*-mediated NF-κB activation may represent a conserved oncogenic mechanism across different malignancy types. Beyond its effects on macrophages, SPRED3 may also influence the infiltration or activity of other anti-tumor immune cell subsets. Indeed, we observed a negative correlation between *SPRED3* expression and the infiltration of activated NK cells, CD8+ T cells, and activated memory CD4+ T cells. NK cells and CD8+ T cells are critical effector cells in anti-tumor immunity, capable of directly eliminating tumor cells (40), while activated memory CD4+ T cells play a significant role in sustaining long-term immune responses (41). The inhibitory effect of *SPRED3* on the infiltration of these cells may therefore compromise the host’s immune surveillance and tumor clearance capabilities, further reinforcing an immunosuppressive microenvironment.

The TME is increasingly recognized as a critical determinant of immunotherapy efficacy (42, 43). In recent years, a growing number of TME modulators have been identified across various cancer types, functioning through diverse signaling mechanisms. For instance, *TRIM29* has been reported to promote glioblastoma malignancy through the *NEFL*-PI3K/AKT axis (44), and *PLAU* facilitates tumor progression via perineural invasion in head and neck squamous cell carcinoma (45). Additionally, the regulatory roles of other TME-associated molecules, such as *MARCH6* in ferroptosis modulation (46) and liquid biopsy-based biomarkers in precision oncology (47), further underscore the complexity and clinical relevance of the TME landscape. In this context, our findings position *SPRED3* as a novel TME modulator that may influence immunotherapy response through multiple mechanisms. First, by promoting M2 macrophage polarization and suppressing the infiltration of cytotoxic lymphocytes, *SPRED3* may contribute to an immune-excluded, immunosuppressive TME. Second, the positive correlation between *SPRED3* and immune checkpoint molecules (PD-L1, PD-L2, CD80, CD86, and CTLA-4) suggests that *SPRED3* may directly participate in checkpoint-mediated T cell dysfunction, further reinforcing the immunosuppressive landscape. Notably, the NF-κB signaling pathway, which we have identified as a key mediator of *SPRED3* function, has also been implicated in the pro-tumorigenic effects of other TME modulators. For instance, *WDR54* has recently been reported to promote hepatocellular carcinoma progression through activation of the NF-κB pathway (48), highlighting that NF-κB activation represents a common mechanism by which diverse oncogenic drivers shape the immunosuppressive TME. Collectively, our observations support a model in which *SPRED3* contributes to an immune-excluded, immunosuppressive tumor microenvironment, thereby diminishing the likelihood of a favorable response to immunotherapy. This is further corroborated by our TIDE analysis, which predicted poorer immunotherapy outcomes in *SPRED3*-high patients.

In addition, gastric cancer patients with high *SPRED3* expression exhibited higher TIDE scores, which indicates that these patients may have a poor response to immunotherapy. Interestingly, the expression of SPRED3 in gastric cancer showed a positive correlation with the levels of various important immune checkpoint molecules, including PD-L1, PD-L2, CD80, CD86, and CTLA-4. PD-L1 and PD-L2 inhibit T cell activation by binding to PD-1 (49), while CD80 and CD86 are crucial for T cell co-stimulation and immune regulation (50). Additionally, CTLA-4, an immune inhibitory molecule, suppresses T cell activation and proliferation (51). These findings collectively suggest that SPRED3 may enhance the immunosuppressive microenvironment by upregulating the expression of these immune checkpoint molecules. This enhancement facilitates the evasion of immune surveillance by tumor cells, thereby promoting tumor growth and progression.

The finding that alpelisib directly binds to SPRED3 and downregulates its expression is noteworthy, as alpelisib is a class I PI3K inhibitor with marked selectivity for the PI3Kα isoform (52). This observation raises the possibility that SPRED3 may represent an off-target effector of alpelisib, or alternatively, that SPRED3 functions downstream of or in crosstalk with the PI3K/AKT pathway in gastric cancer cells. Although the exact nature of this relationship remains to be defined, our data suggest that the anti-tumor effects of alpelisib in this context may involve, at least in part, the modulation of SPRED3 expression. Further mechanistic studies are warranted to dissect whether SPRED3 acts independently or converges with PI3K/AKT signaling to regulate gastric cancer progression and drug response.

Of note, the GSE65801 dataset used in this study has been previously employed by Sun et al. to screen for differentially expressed genes related to the MYB/SSBP2/ISL1 axis in gastric cancer (53). While their analysis identified SPRED3 as one of the differentially expressed genes in the GSE65801 cohort, SPRED3 was not mentioned, discussed, or functionally characterized in that study, as the authors focused exclusively on the MYB/SSBP2/ISL1 axis. To our knowledge, no prior investigation has systematically characterized SPRED3 as a prognostic and immunological biomarker in gastric cancer. Furthermore, our study integrates three independent datasets (TCGA, GSE65801, and GSE15459), provides experimental validation of SPRED3’s biological functions, and explores its role in immune infiltration and drug sensitivity. Thus, the conceptual novelty and scientific contribution of our study remain unaffected.

This study has several limitations. First, *SPRED3* expression in clinical samples was assessed solely using publicly available transcriptomic data (TCGA and GEO cohorts), without protein-level validation in patient tissues. Although mRNA expression often correlates with protein levels, post-transcriptional regulation may lead to discrepancies. Notably, MKN45 cells exhibited elevated *SPRED3* mRNA without a corresponding increase at the protein level, further underscoring the need for protein-based validation in independent clinical cohorts in future studies. Second, the functional experiments were conducted exclusively *in vitro*; therefore, *in vivo* models and clinical investigations are warranted to fully elucidate the biological role of *SPRED3* in gastric cancer progression. Additionally, although alpelisib was found to bind SPRED3 and downregulate its expression, its effects on SPRED3 post-translational modifications or protein-protein interactions were not explored, and represent an important direction for future investigations.

## 5 Conclusion

In conclusion, *SPRED3* is highly expressed in gastric cancer and serves as a potential diagnostic biomarker. Its upregulation is associated with poor prognosis and promotes gastric cancer cell progression, partially through MAPK and NF-κB pathways. SPRED3 expression correlates with immune cell infiltration and immune checkpoint expression, contributing to immune exclusion and an immunosuppressive tumor microenvironment. Low *SPRED3* expression may predict increased sensitivity to alpelisib. Conceptually, this study introduces *SPRED3* as a previously unrecognized regulator in gastric cancer pathogenesis and immunity. Further research is warranted to explore its mechanisms and clinical applications.

## Data Availability

The original contributions presented in the study are included in the article/**Supplementary Material**. Further inquiries can be directed to the corresponding author.

## Supplementary material

Supplementary Figure S1 The primary images of the blots.

Supplementary Figure S2 Correlation between SPRED3 expression and key genes in HIF-1, Notch, MAPK, and NF-κB signaling pathways. A. The correlation between SPRED3 and key genes (HIF1A, ARNT) in HIF-1 signaling pathway. B. The correlation between SPRED3 and key genes (NOTCH1, NOTCH2) in Notch signaling pathway. C. The correlation between SPRED3 and key genes (MAPK1, MAPK3) in MAPK signaling pathway. D. The correlation between SPRED3 and key genes (NFKB1, NFKB2) in NF-κB signaling pathway.

Supplementary Figure S3 SPRED3 knockdown in AGS cells alters macrophage polarization and migration via regulating CCL2 and CCL5 secretion. A-B. The levels of CCL2 and CCL5 in the conditioned medium from AGS cells transfected with si-NC or si-SPRED3 were measured by qRT-PCR (A) and ELISA (B), respectively. C. The expression of M0 phenotype marker CSF1R in macrophages co-cultured with conditioned medium from AGS cells transfected with si-NC or si-SPRED3 was measured by qRT-PCR. D. The expression of M1 markers iNOS and IL-6 in macrophages co-cultured with conditioned medium from AGS cells transfected with si-NC or si-SPRED3 was measured by qRT-PCR. E. The expression of M2 markers ARG1, IL-10, and CD206 in macrophages co-cultured with conditioned medium from AGS cells transfected with si-NC or si-SPRED3 was measured by qRT-PCR. F. Macrophage migration after co-culture with conditioned medium from AGS cells transfected with si-NC or si-SPRED3 was evaluated by Transwell assay. * means p < 0.05, ** means p < 0.01, *** means p < 0.001.

Supplementary Table S1 The result of GO and KEGG enrichment analysis.

Supplementary Table S2 The result of GSEA enrichment analysis.

Supplementary Table S3 The result of GSVA enrichment analysis.

Supplementary Table S4 The correlation between SPRED3 expression and the IC50 values of 118 distinct drugs.
