# Supplementary material for: Investigating the impact of Sprouty-related EVH1 domain-containing protein 3 on gastric cancer prognosis and tumor microenvironments
Source: Front Oncol. 2026 Aug 21;16:1791690. doi: 10.3389/fonc.2026.1791690 (PMC13541436; doi:10.3389/fonc.2026.1791690)

The primary images of the blots-Figure 2I--GAPDH

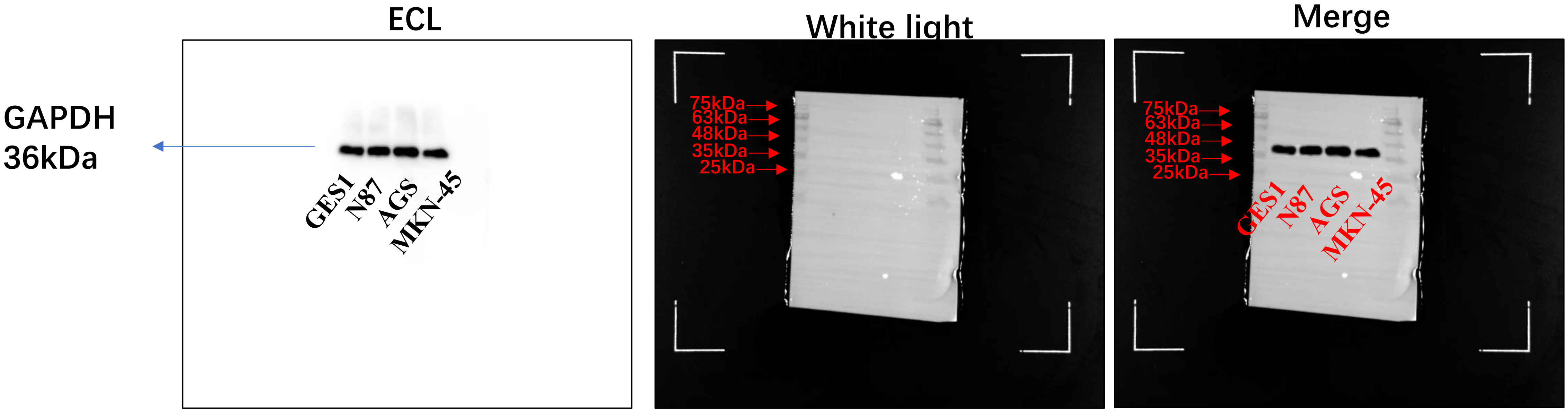

Multiple exposure images

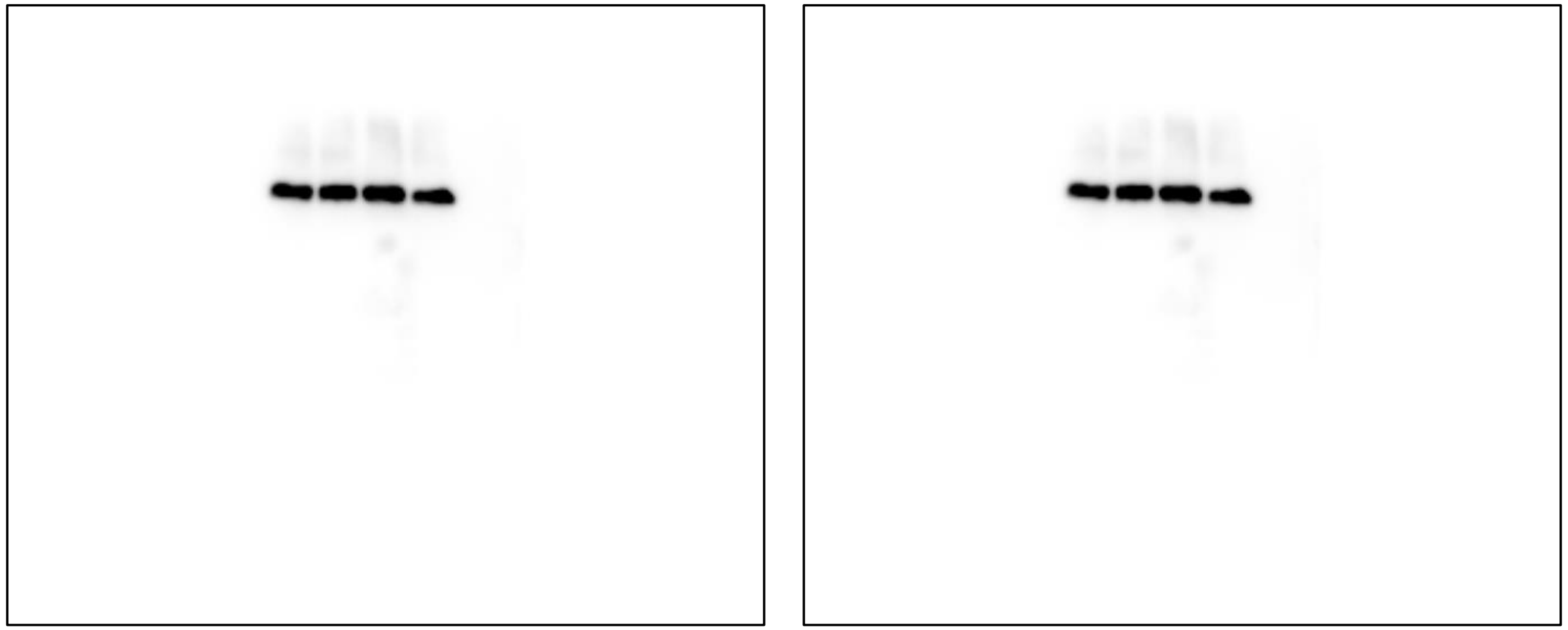

The primary images of the blots-Figure 2I--SPRED3

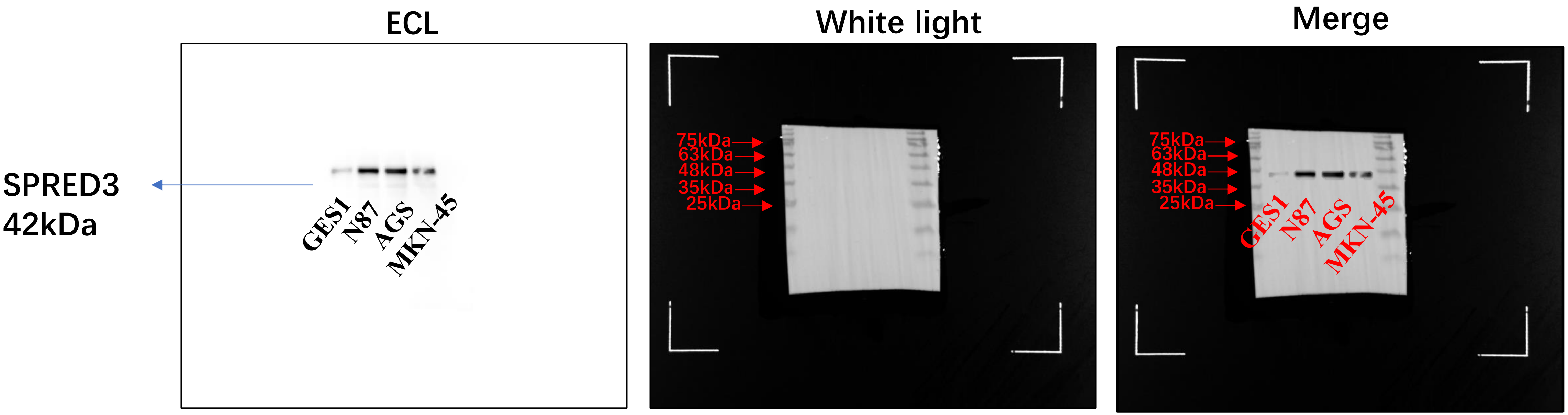

Multiple exposure images

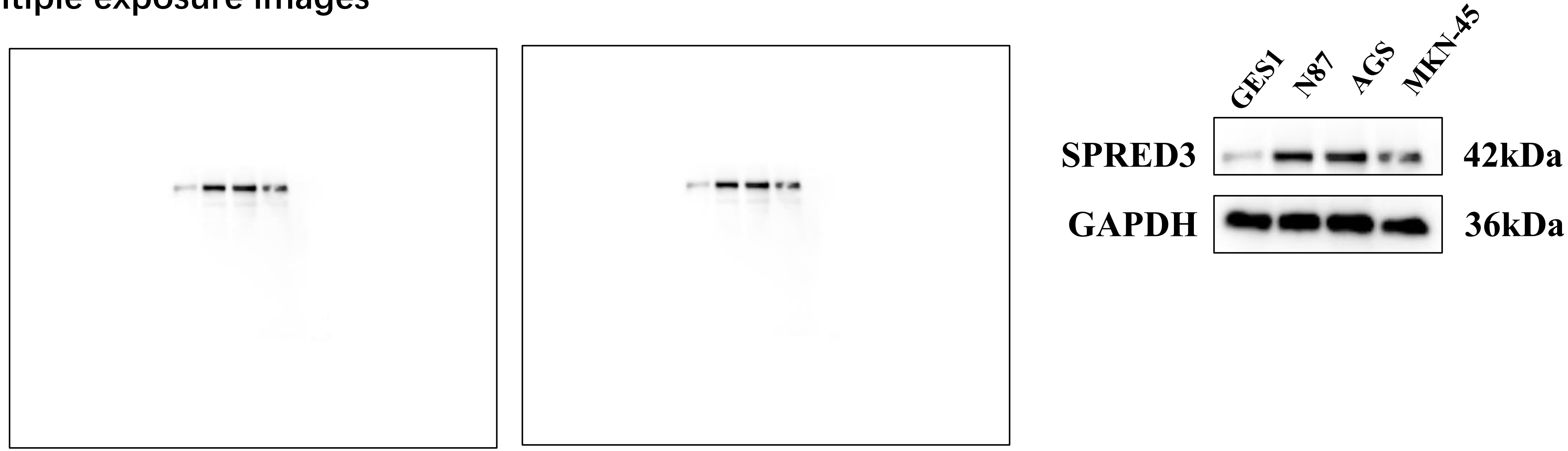

The primary images of the blots-Figure 4B--GAPDH

AGS cell line

ECL

GAPDH
36kDa

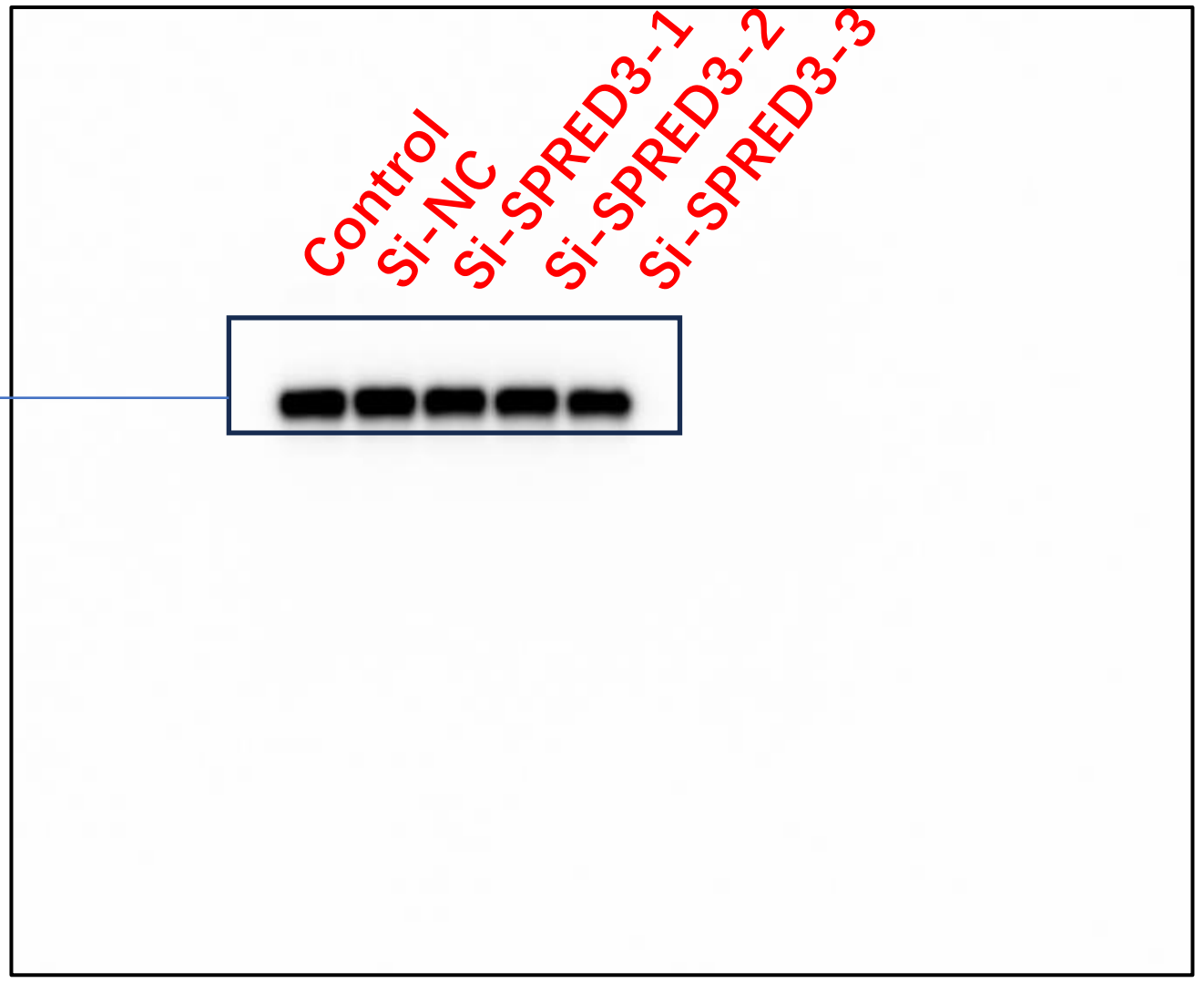

White light

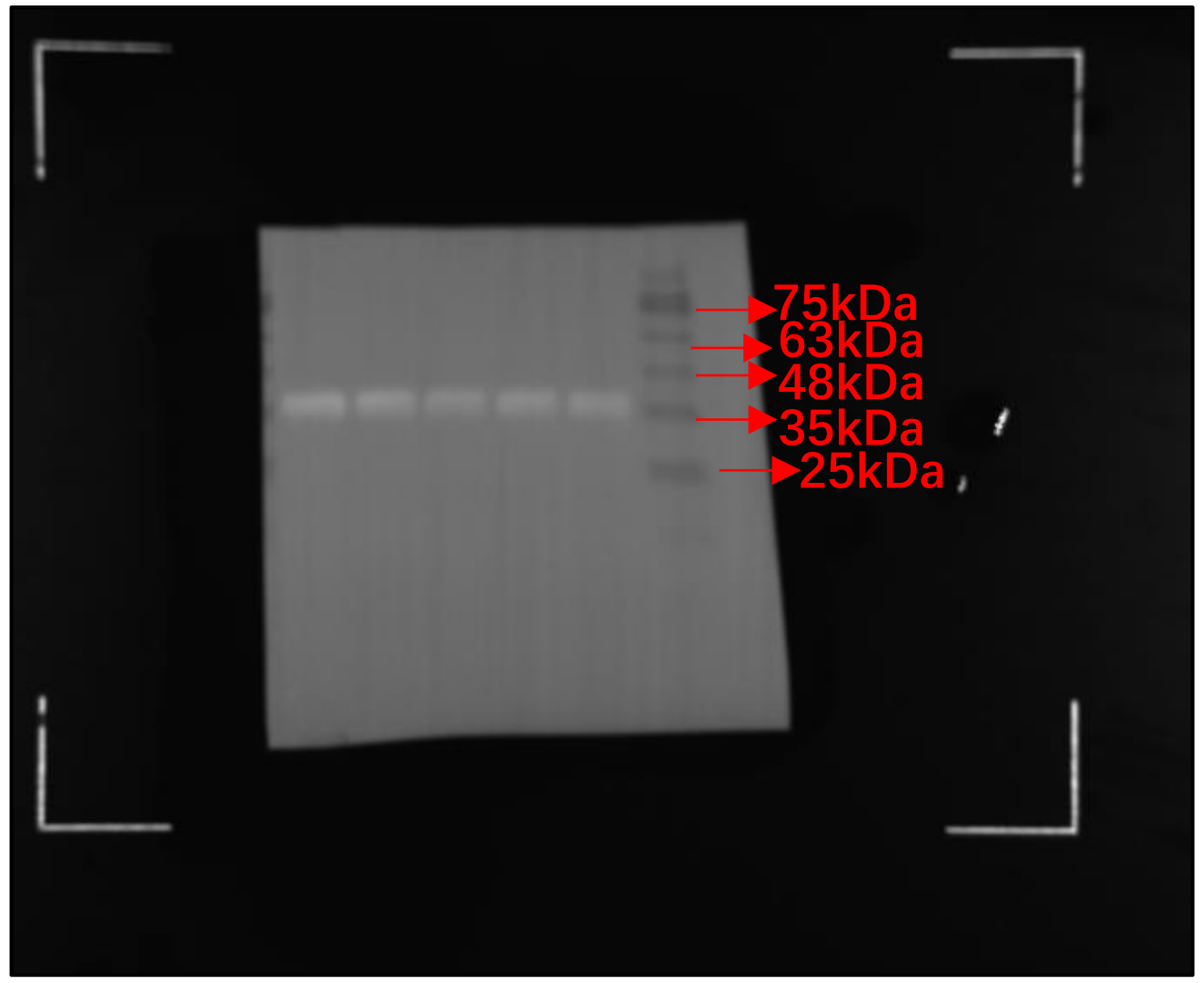

Merge

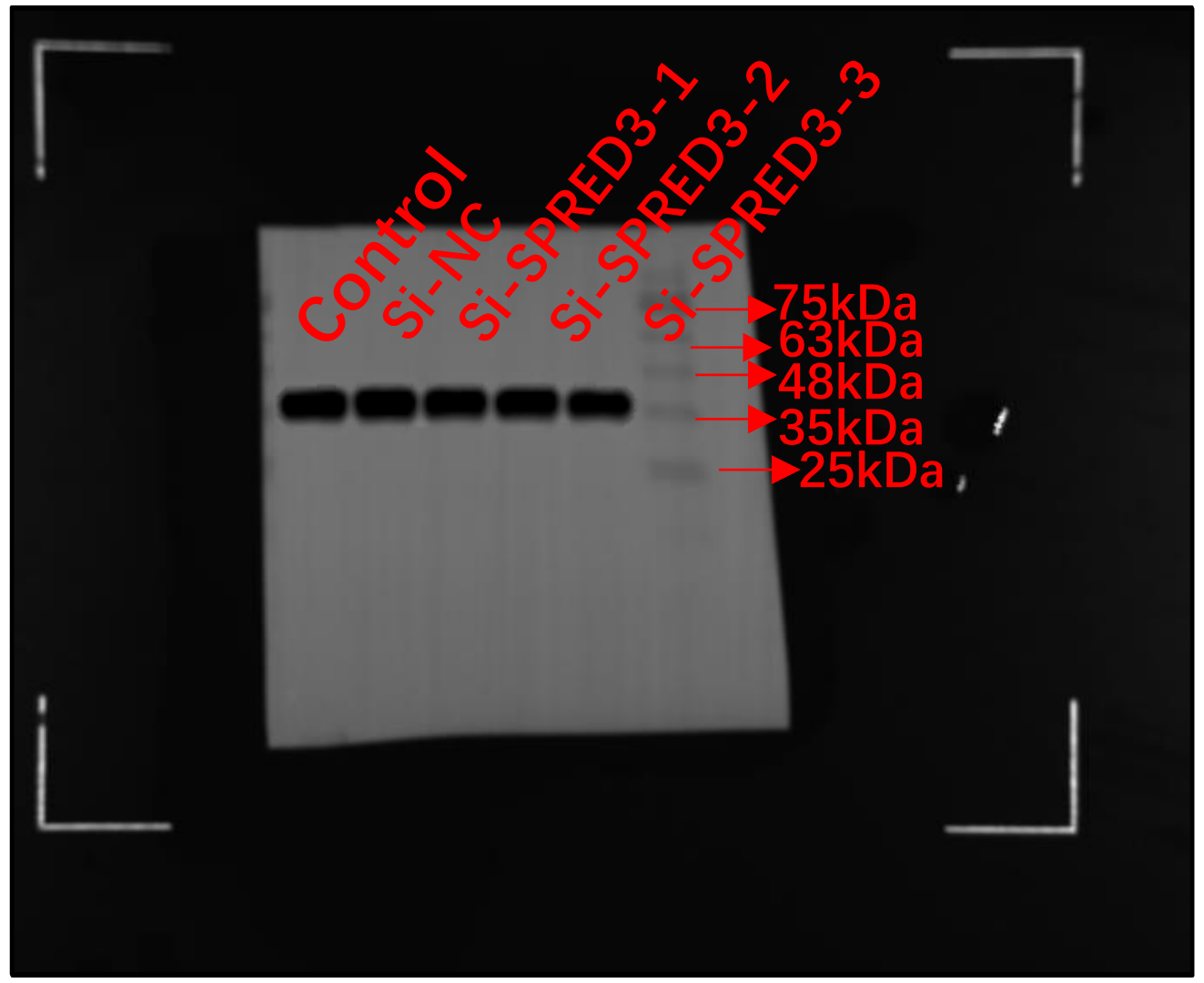

Multiple exposure images

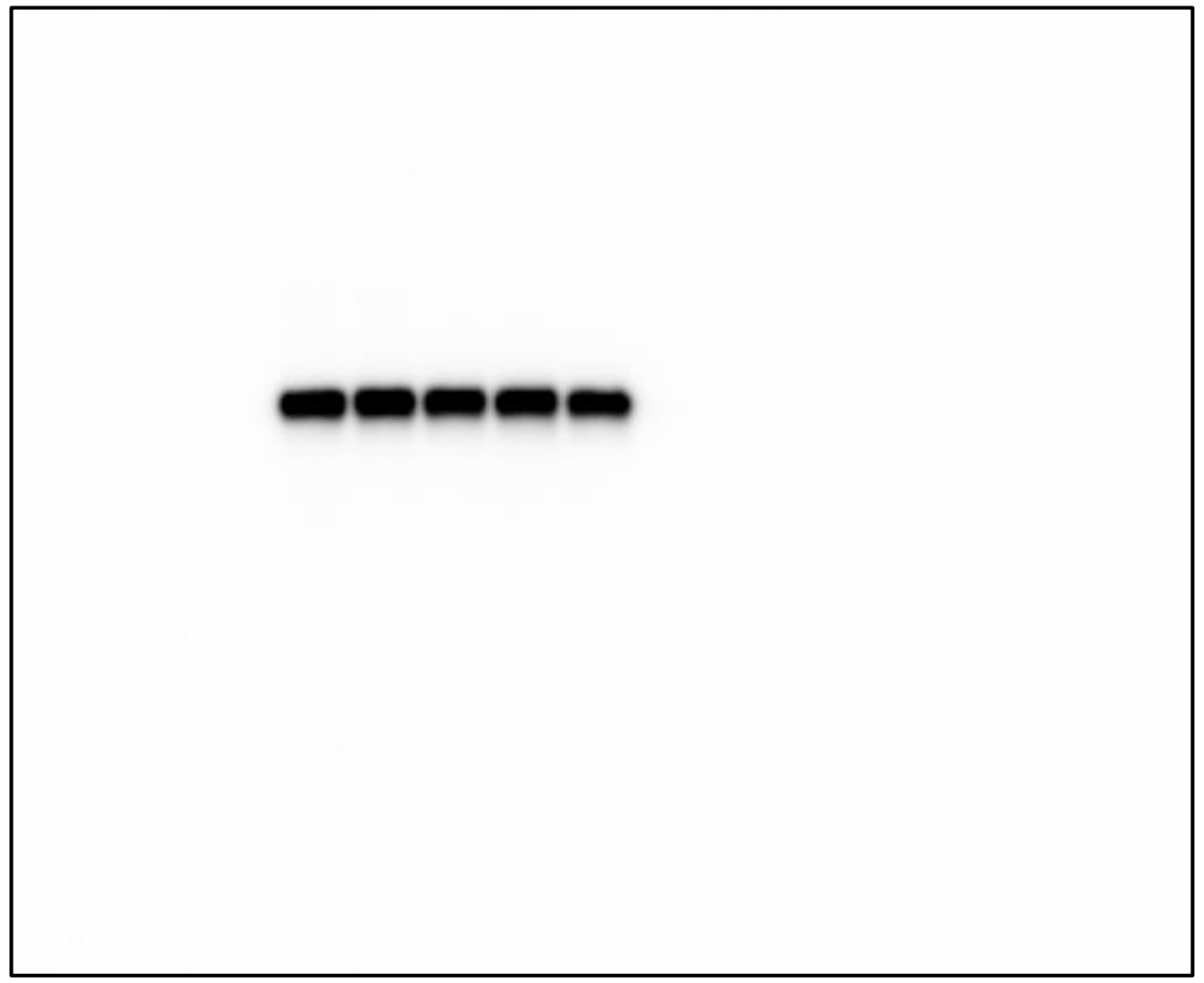

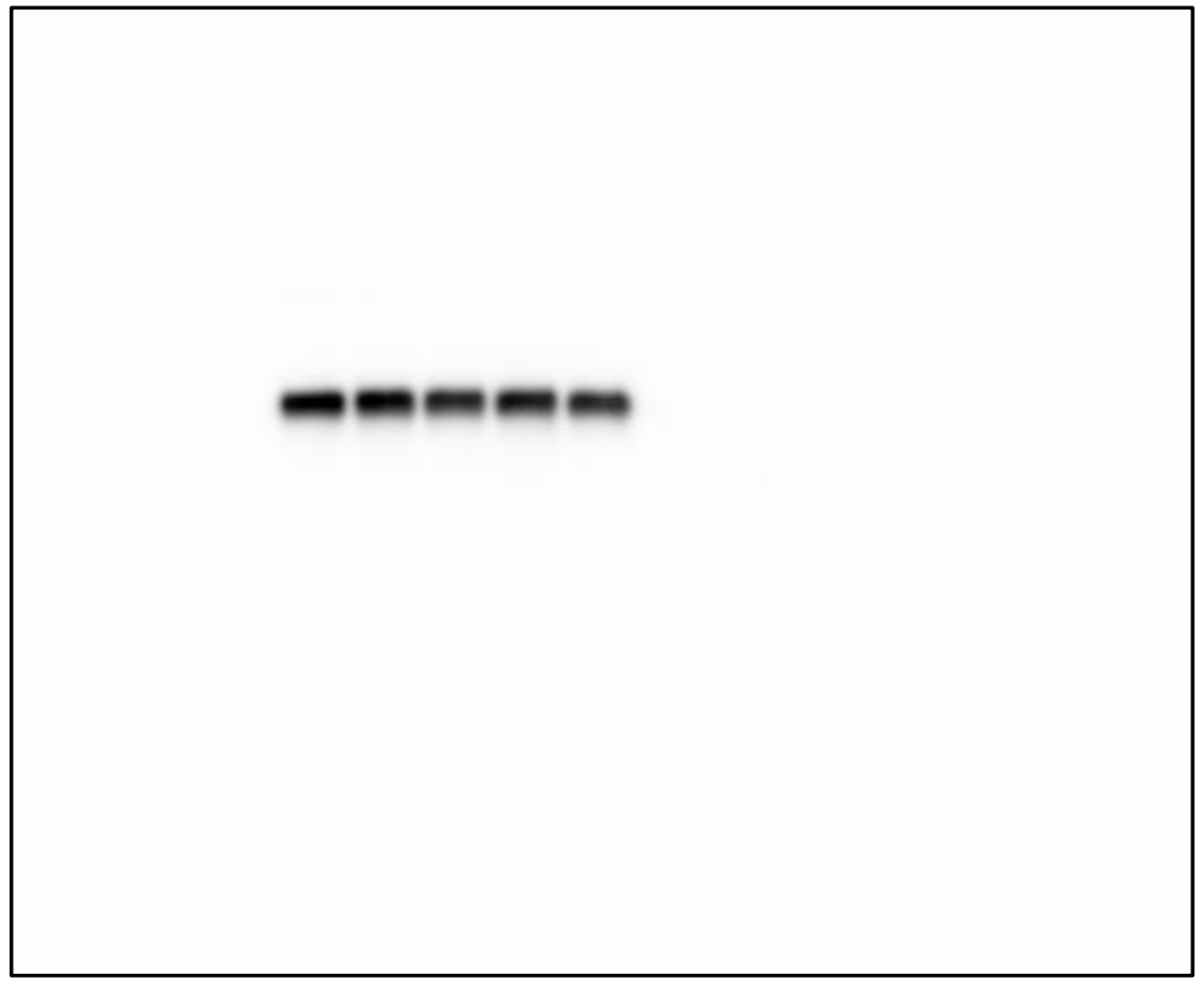

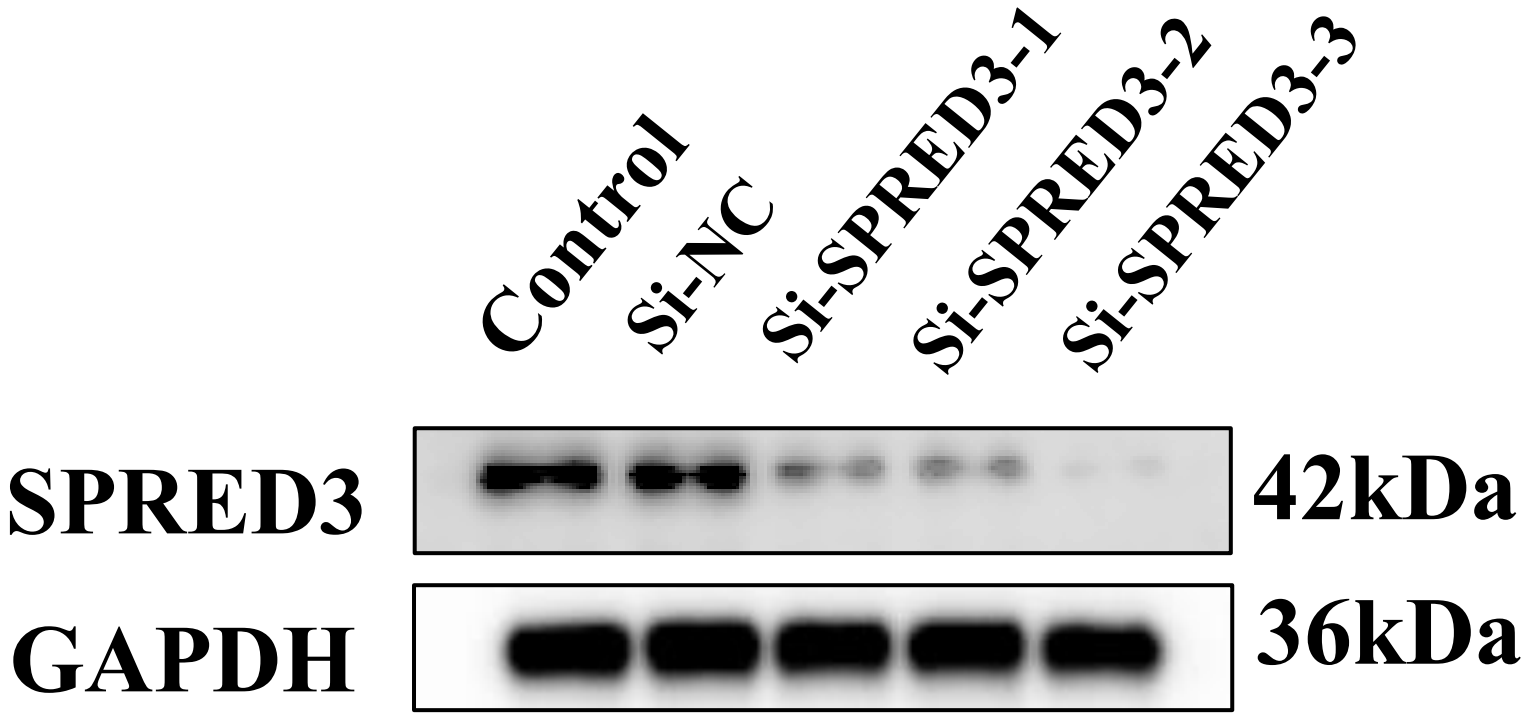

The primary images of the blots-Figure 4B—SPRED3 AGS cell line

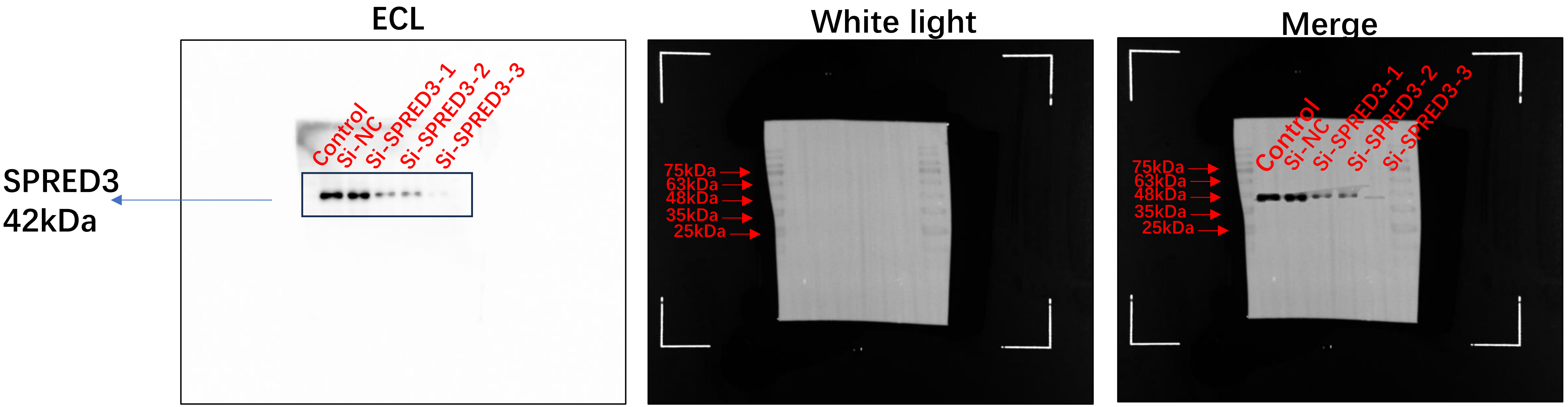

Multiple exposure images

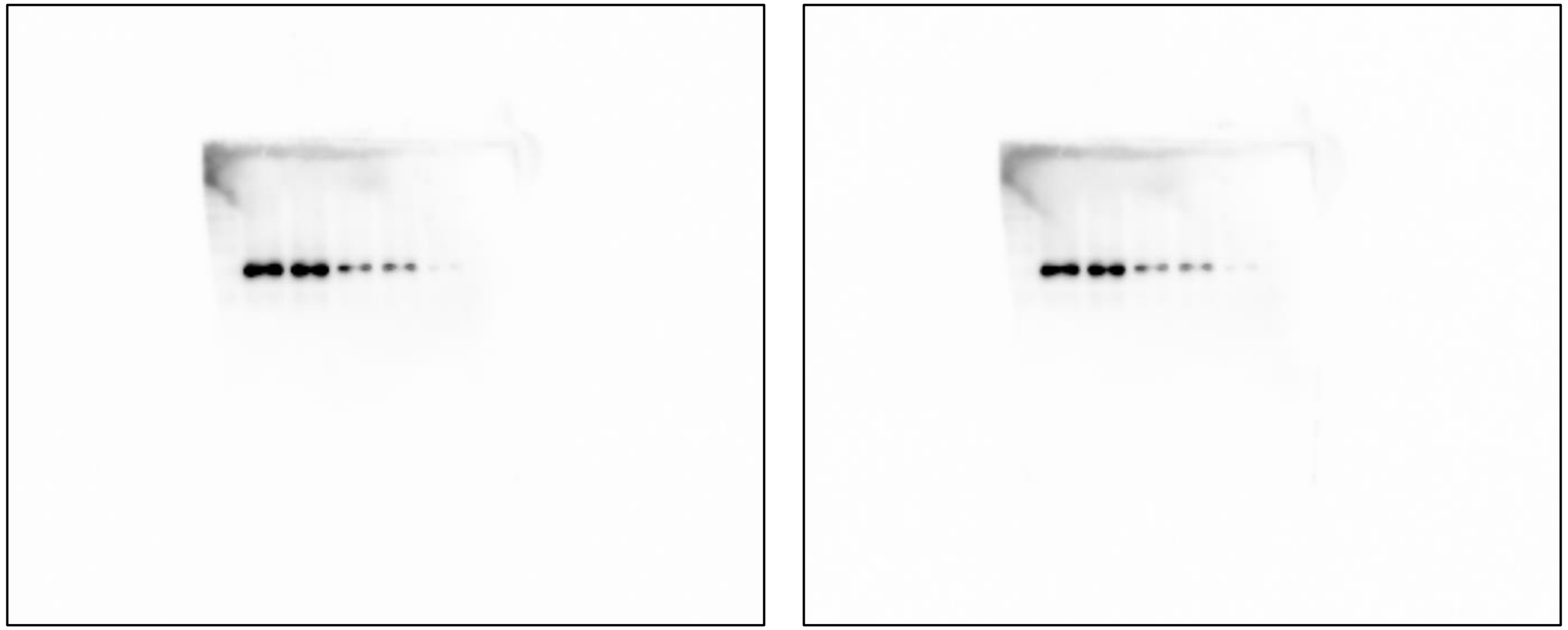

The primary images of the blots-Figure 4D--GAPDH

N87 cell line

ECL

GAPDH
36kDa

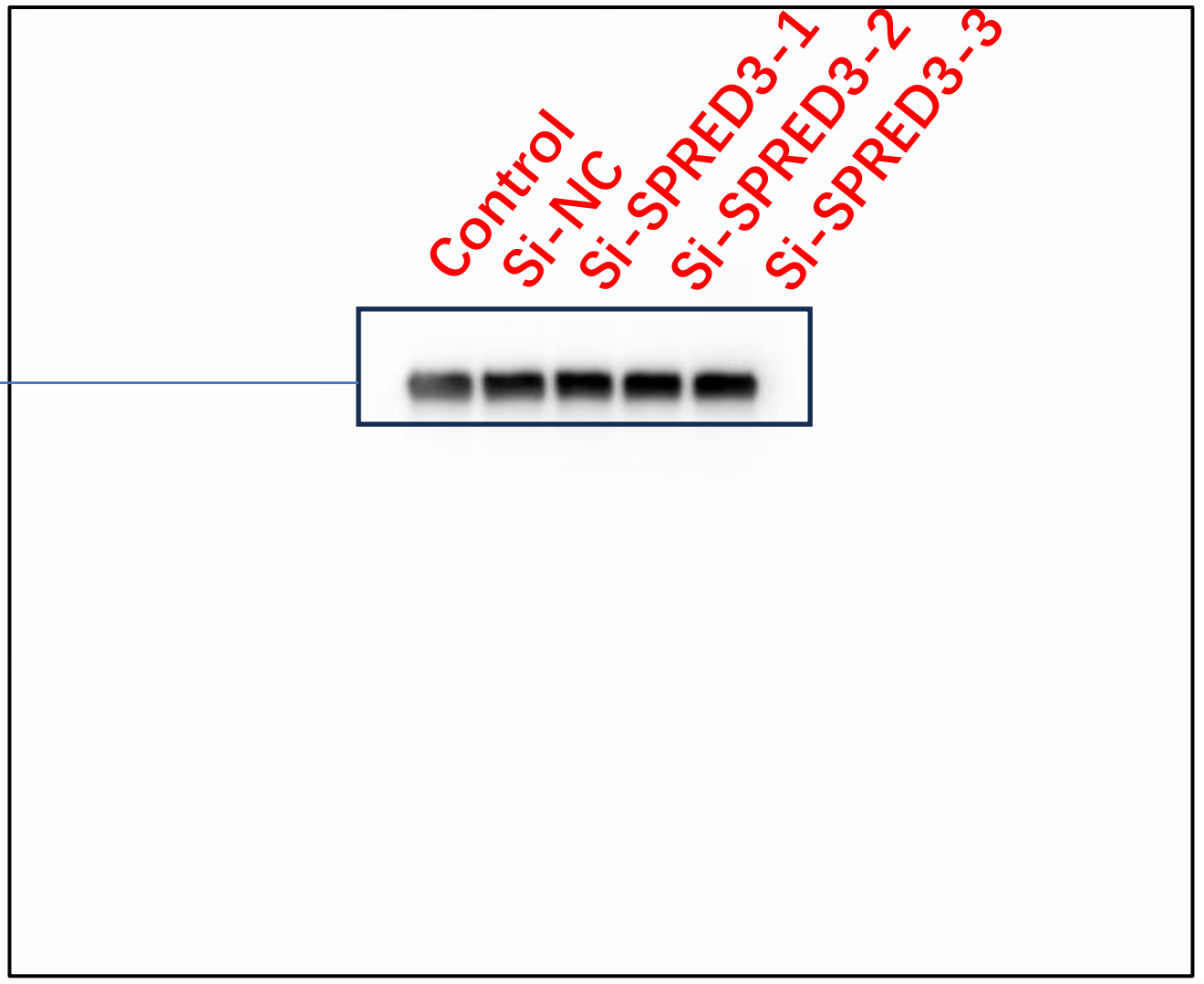

White light

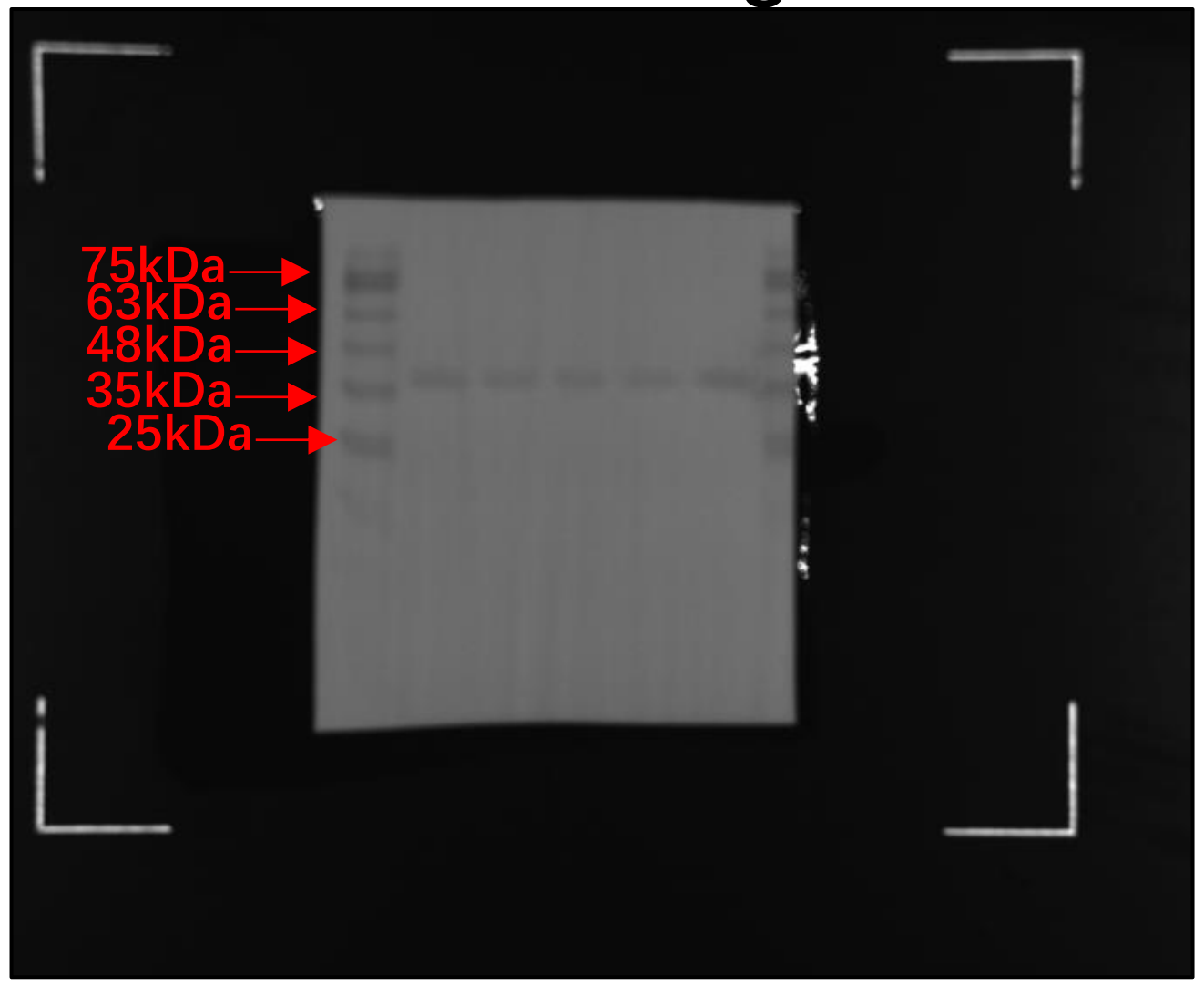

Merge

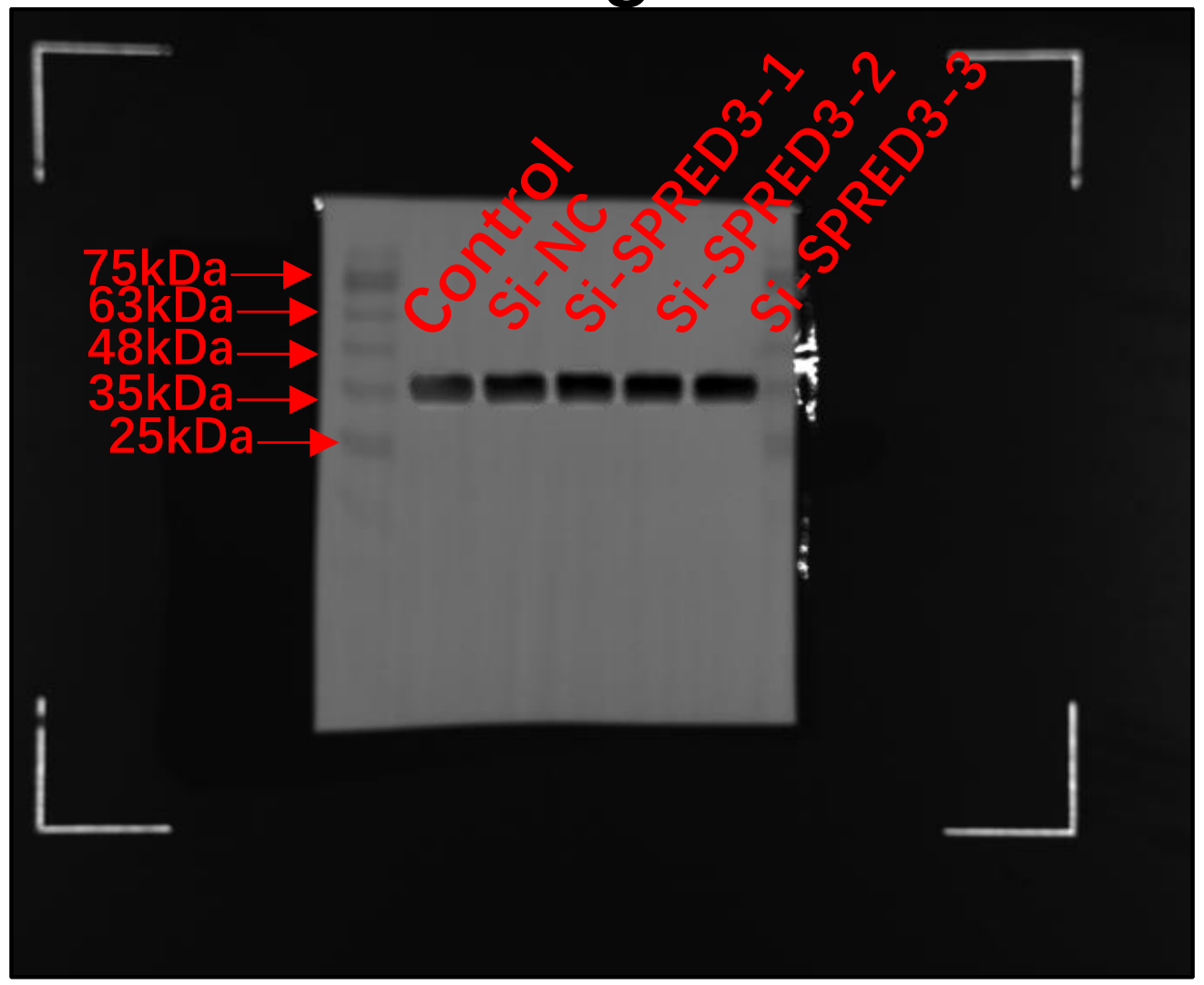

Multiple exposure images

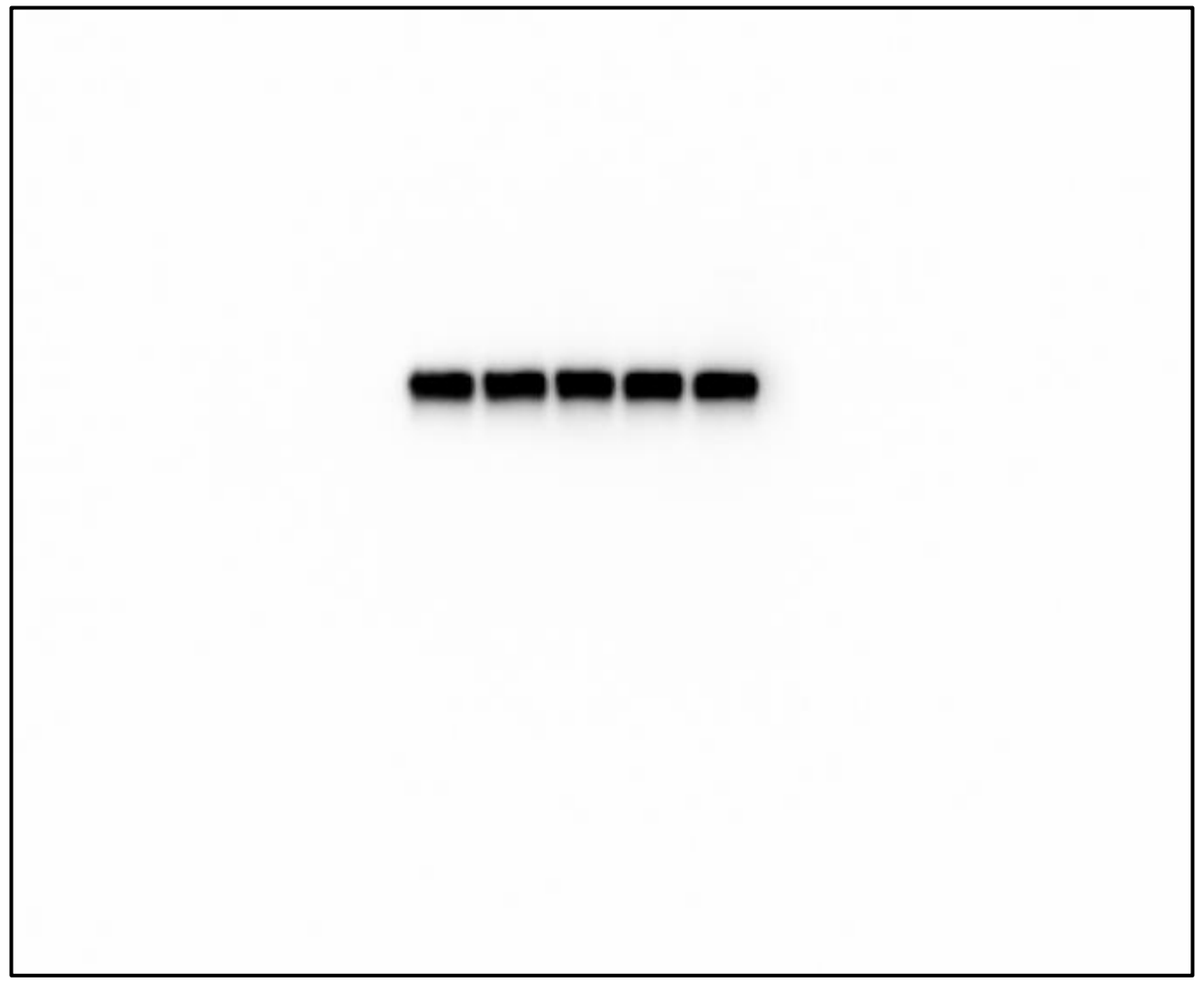

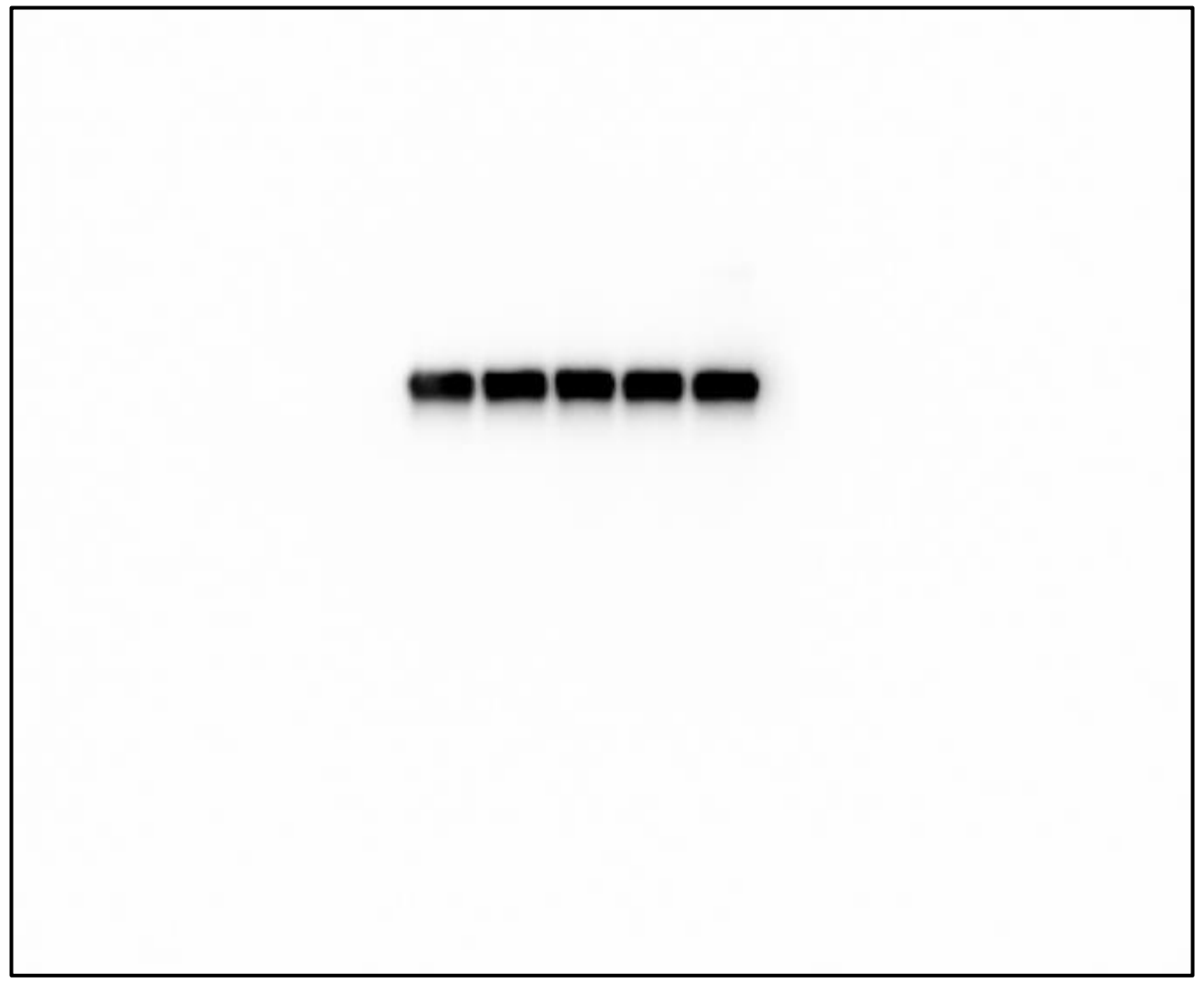

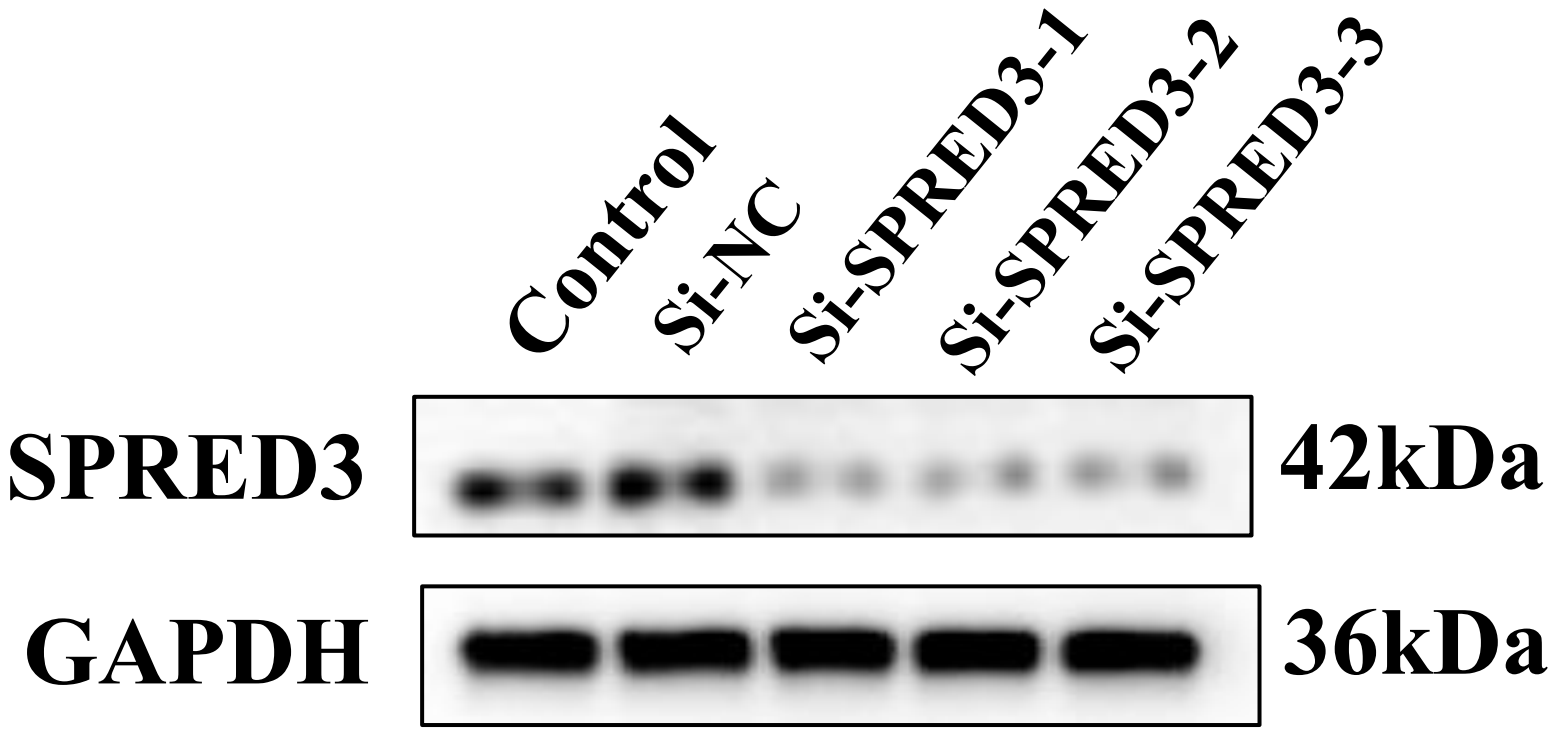

The primary images of the blots-Figure 4D—SPRED3

N87 cell line

ECL

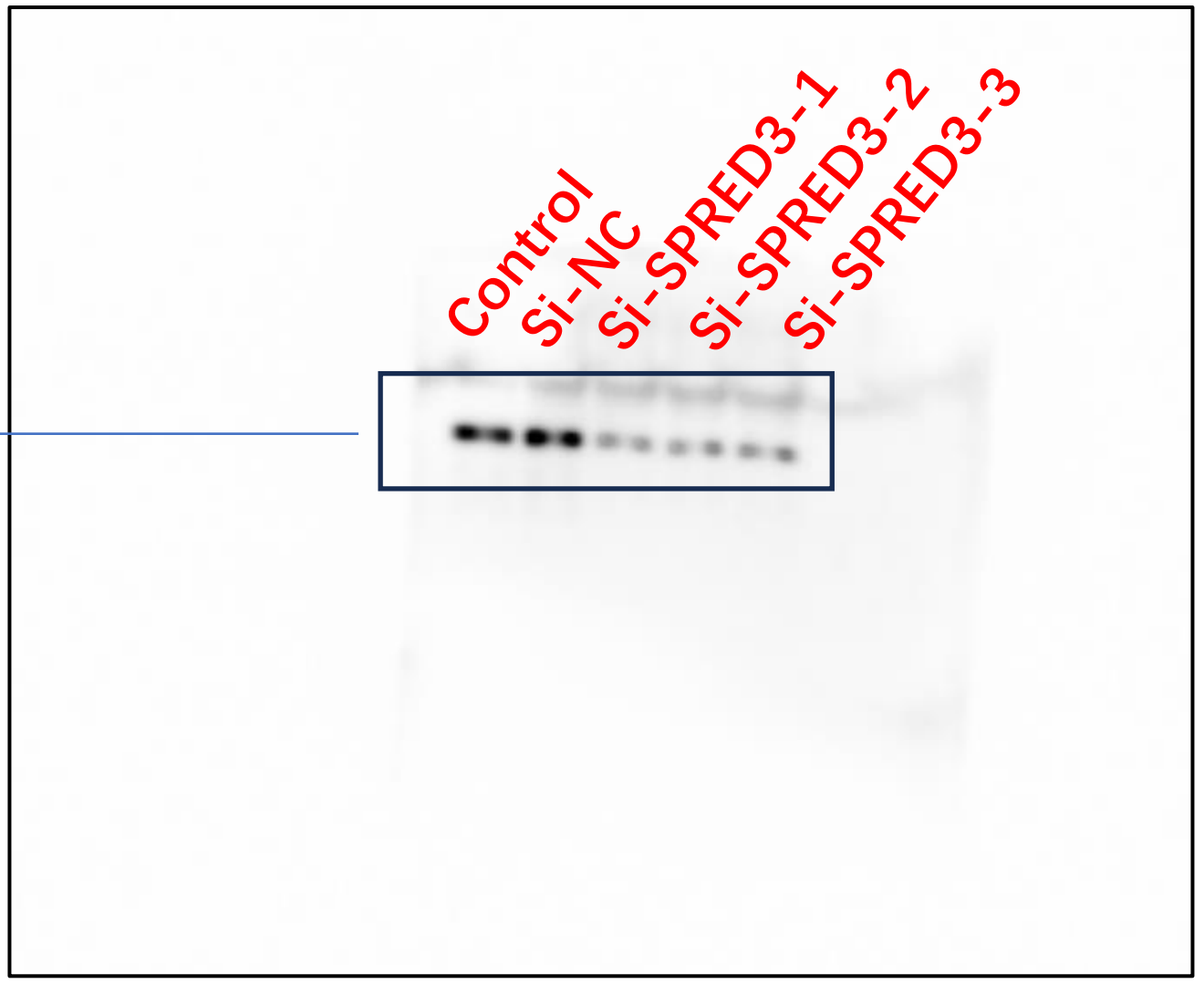

SPRED3
42kDa

White light

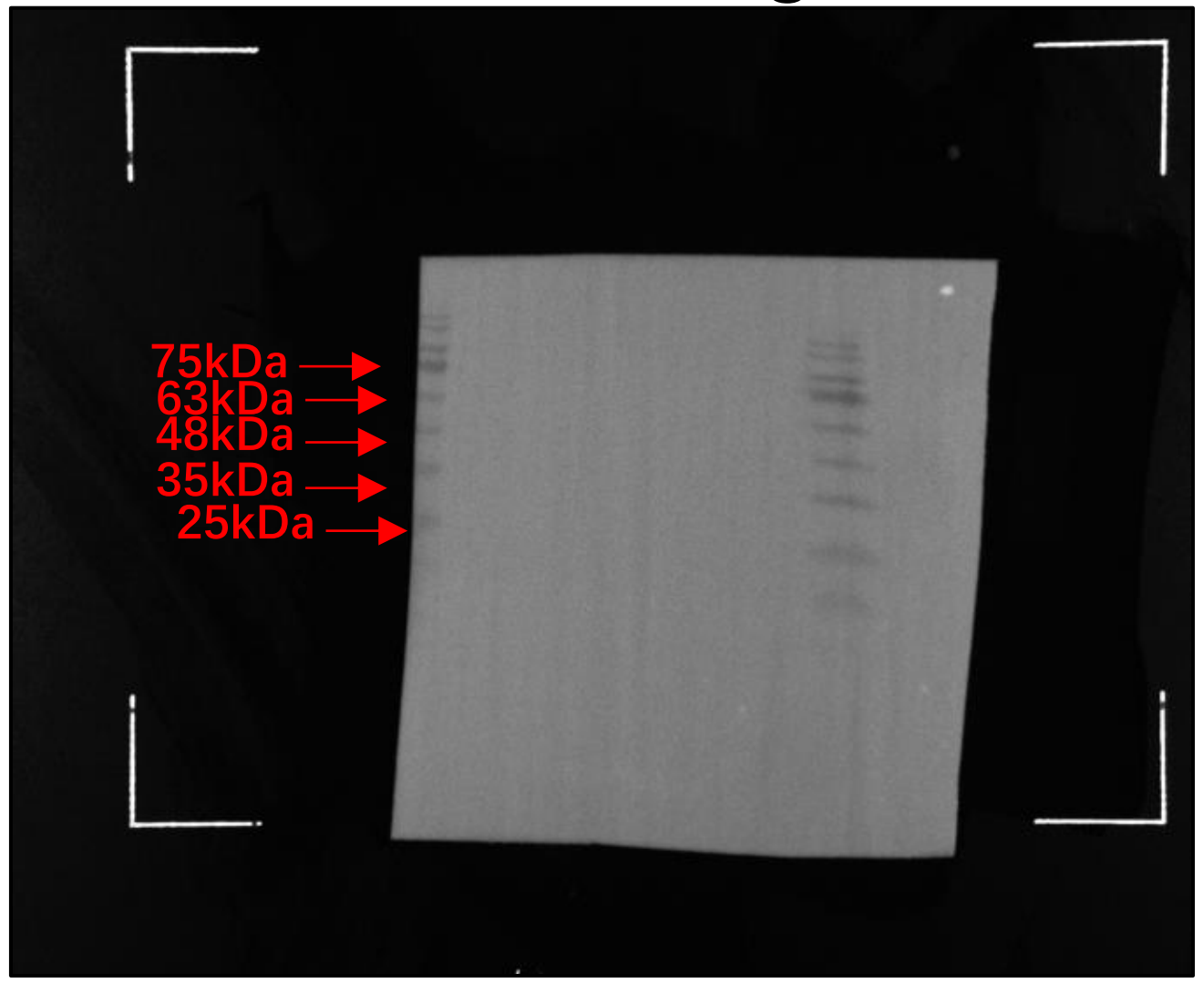

Merge

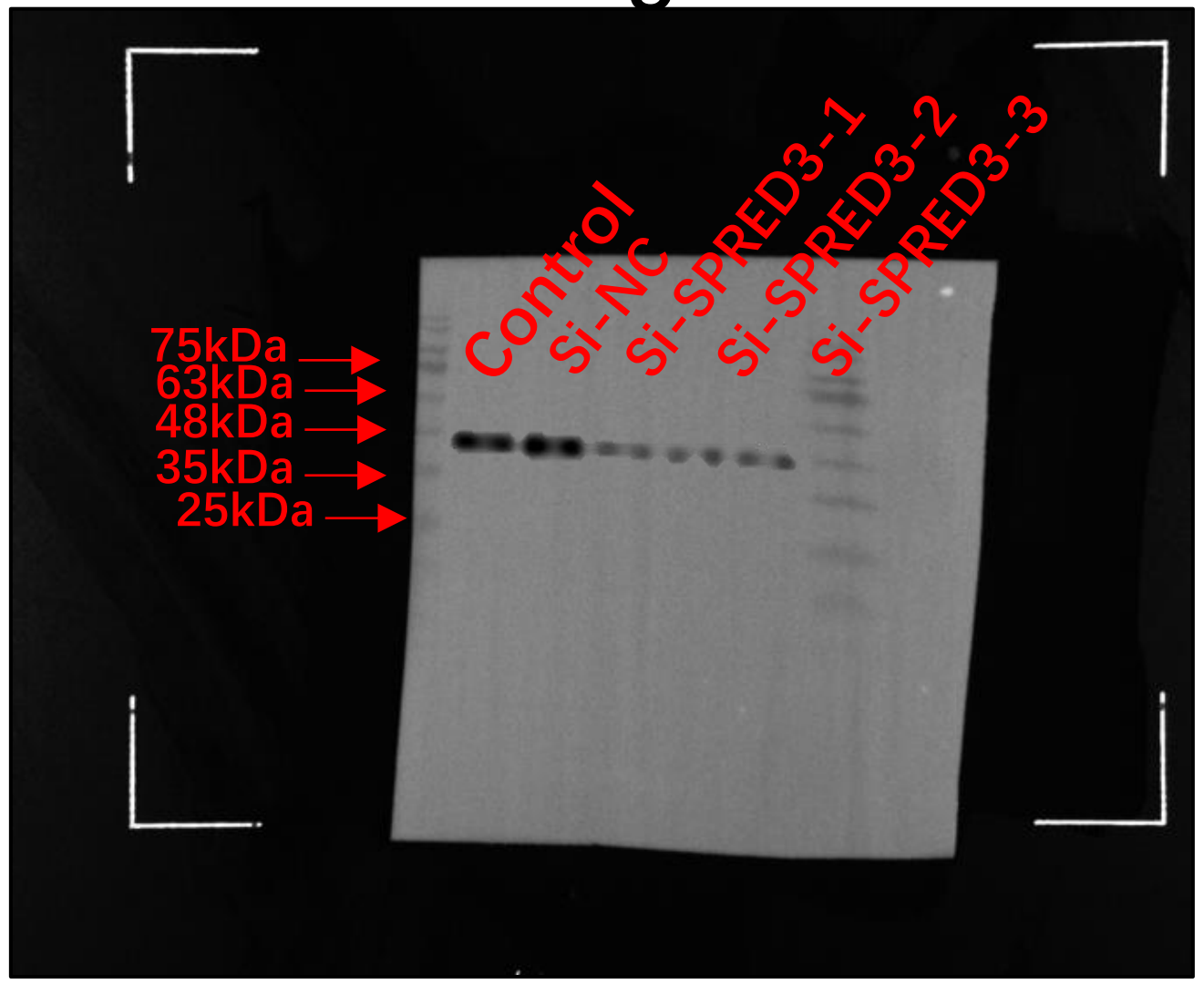

Multiple exposure images

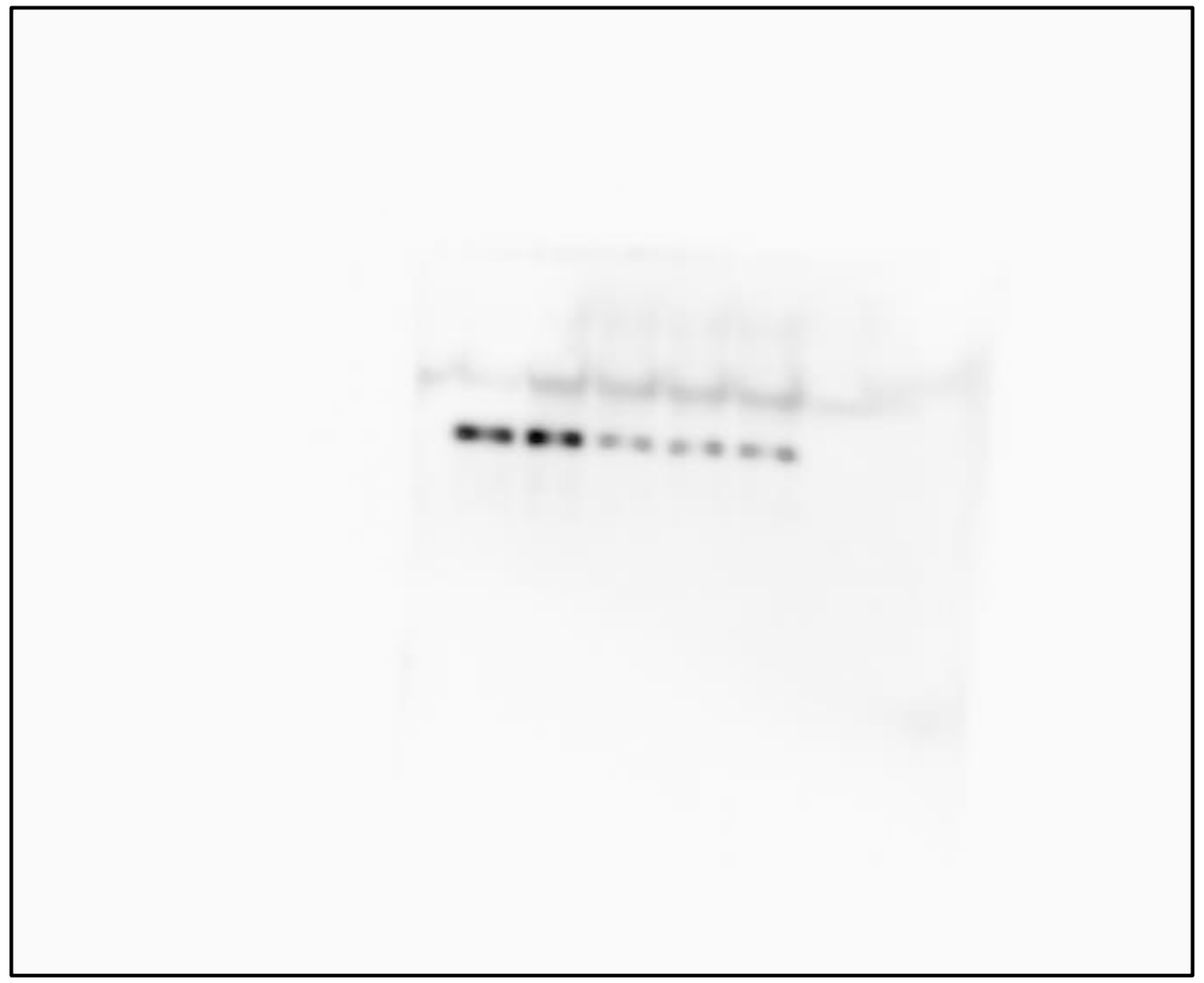

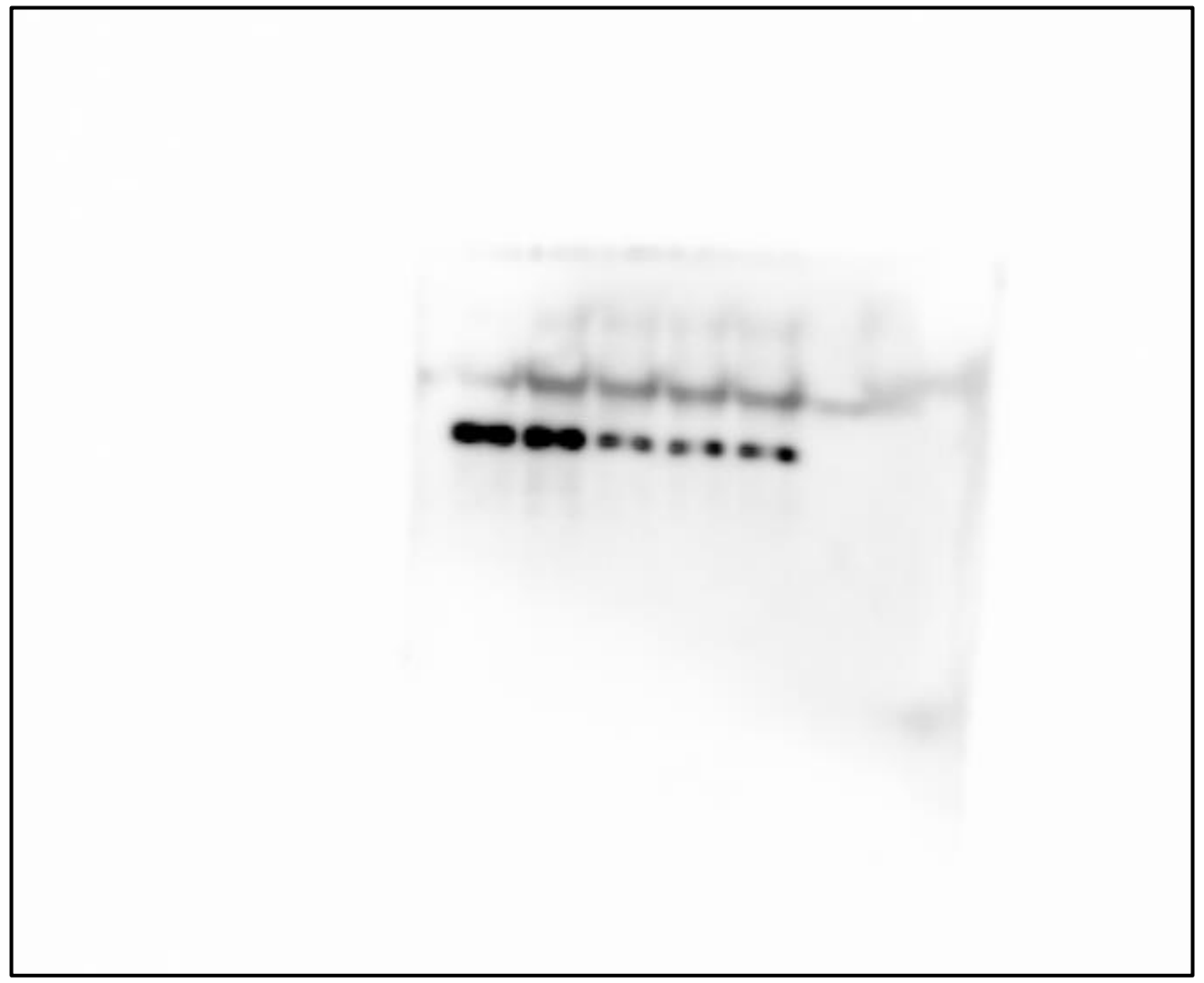

The primary images of the blots-Figure 5E—p-p38

AGS cell line

ECL | White light | Merge

p-p38
38kDa

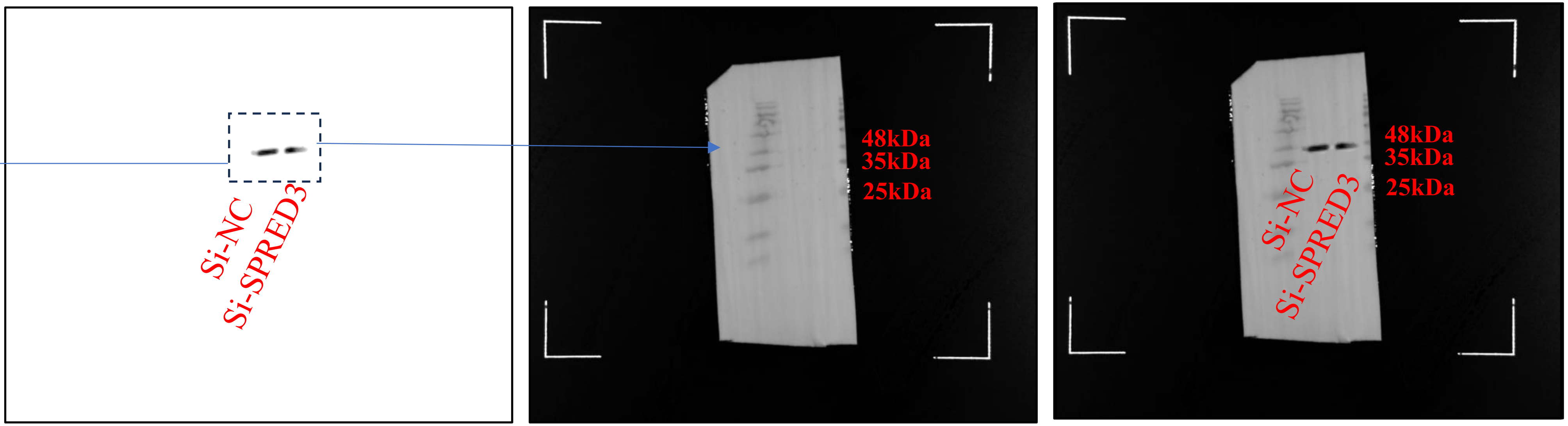

Multiple exposure images

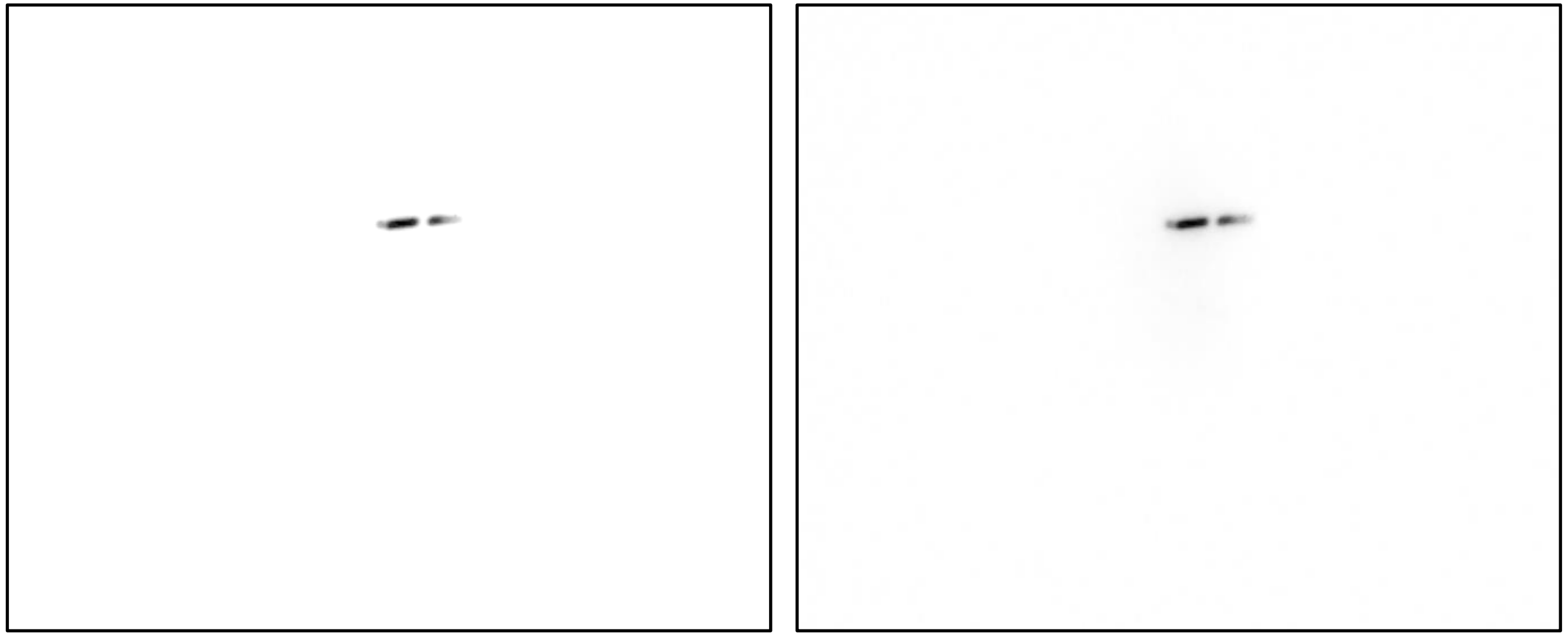

The primary images of the blots-Figure 5E—p38

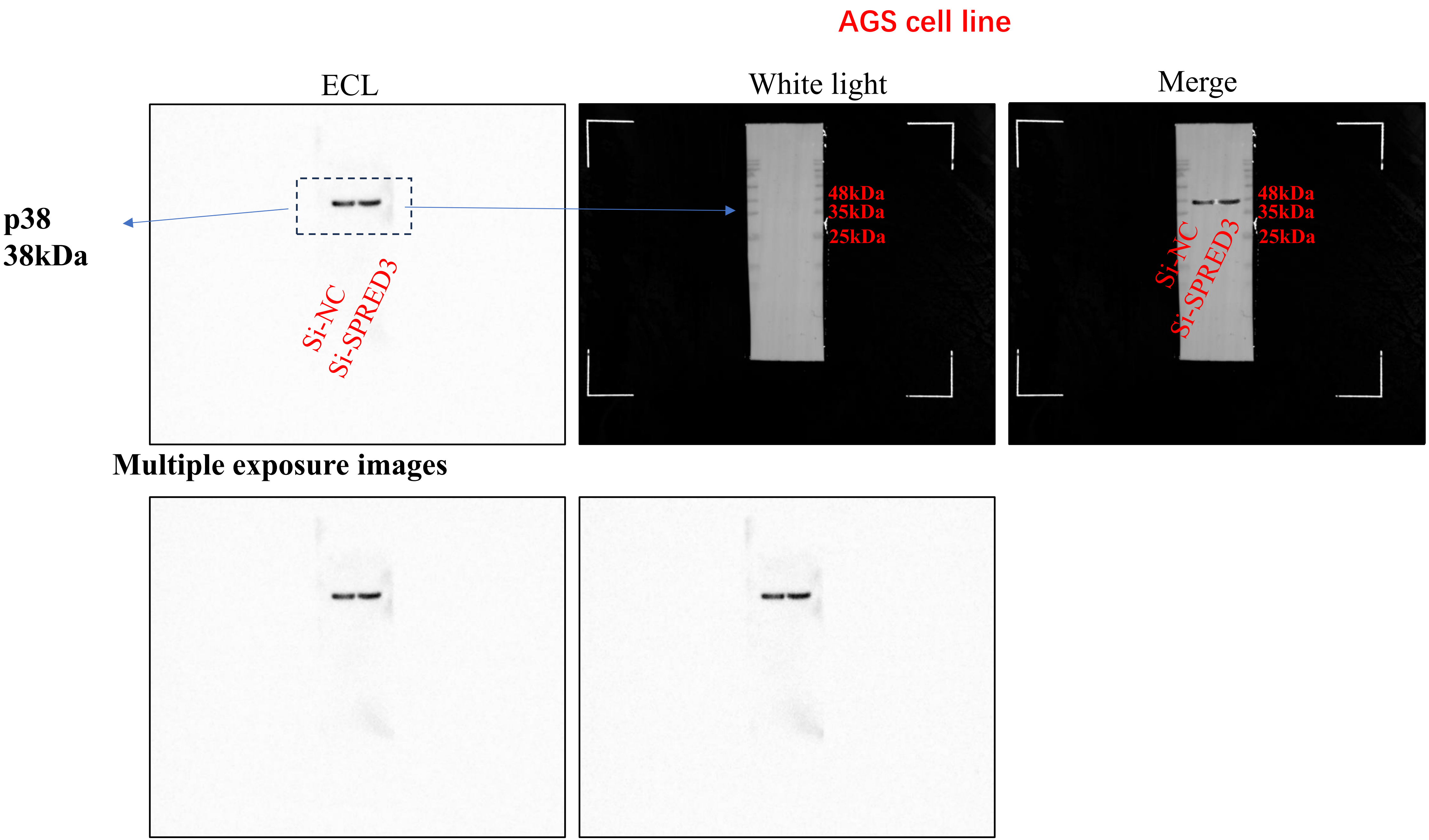

The primary images of the blots-Figure 5E—p-p65

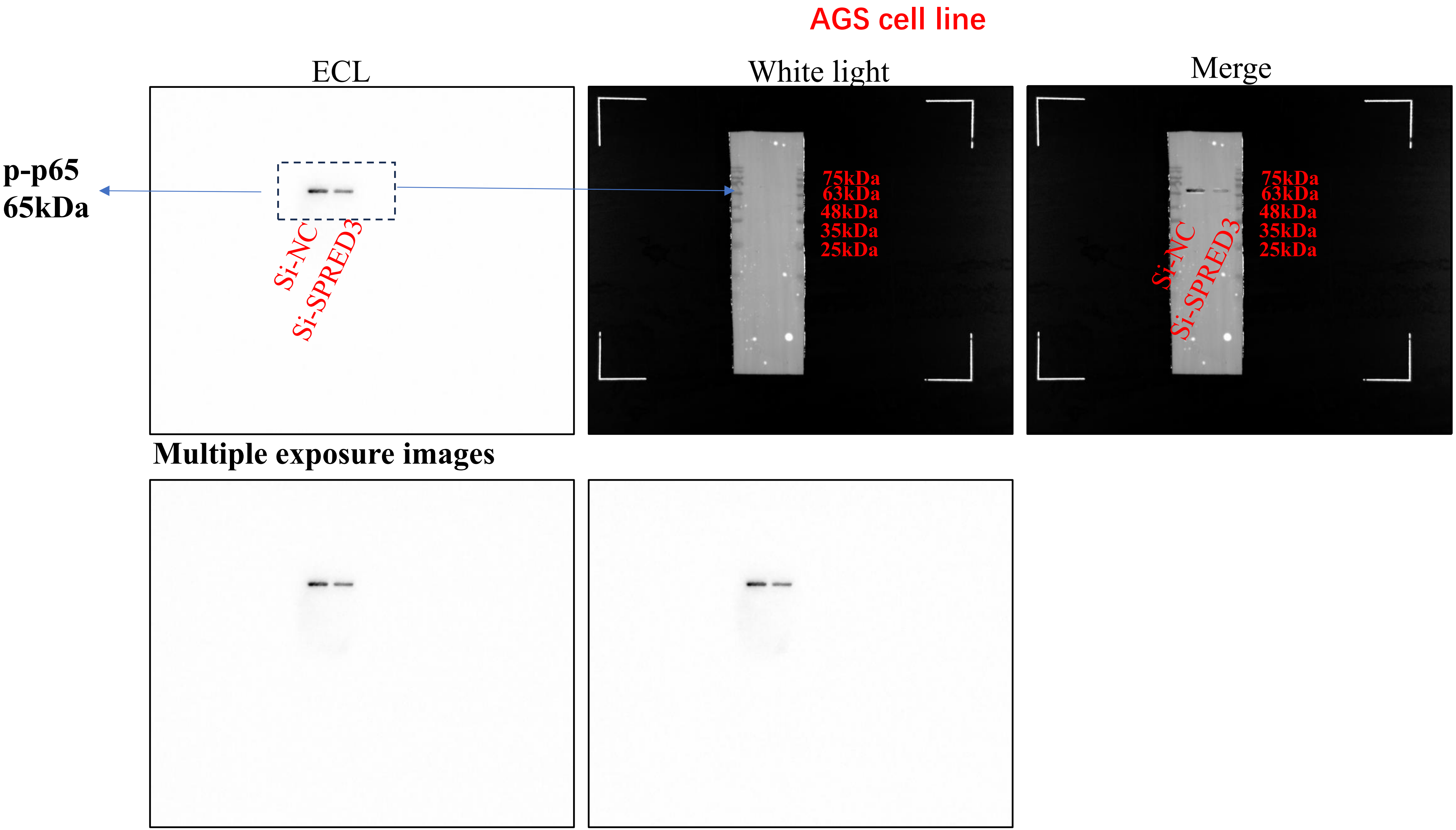

The primary images of the blots-Figure 5E—p65

AGS cell line

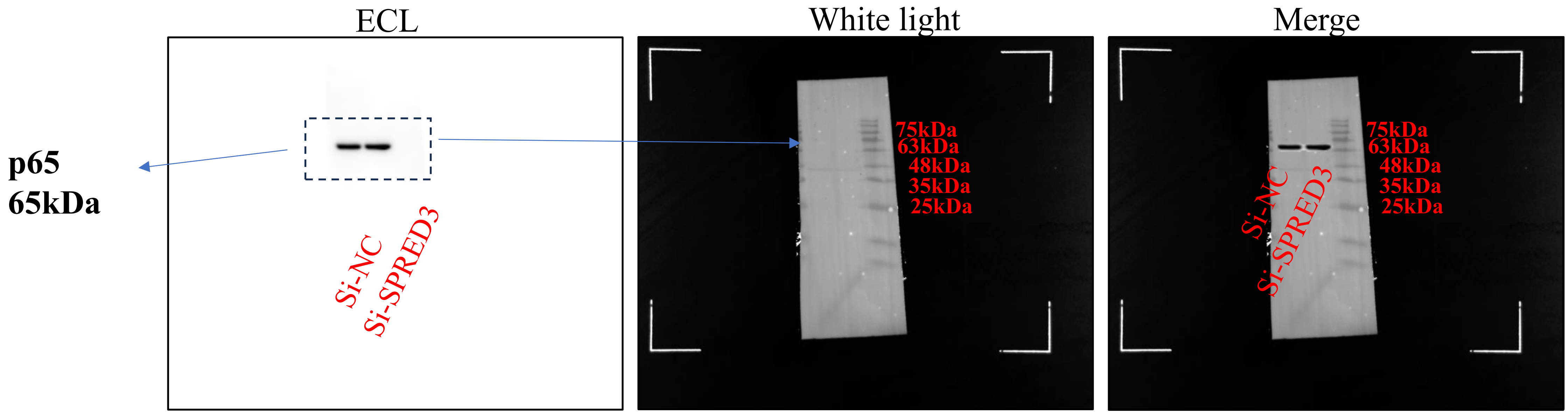

Multiple exposure images

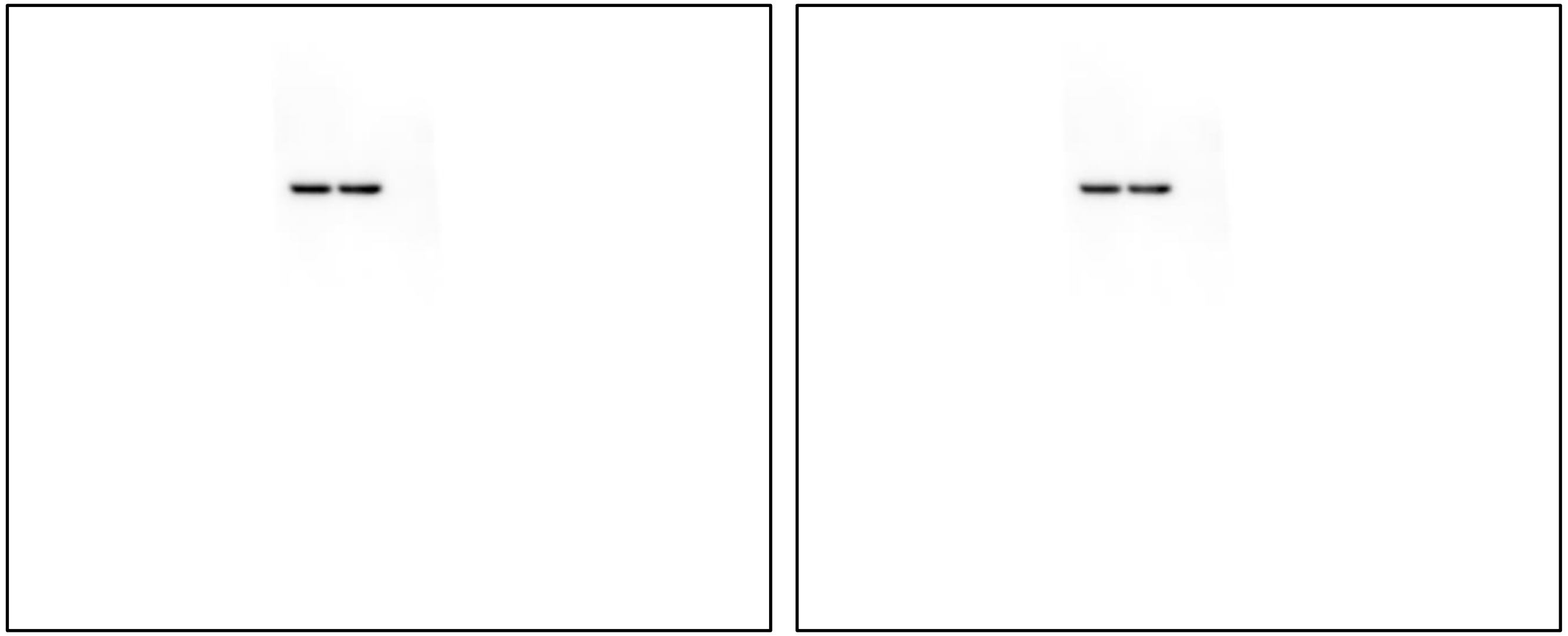

The primary images of the blots-Figure 5E—GAPDH

AGS cell line

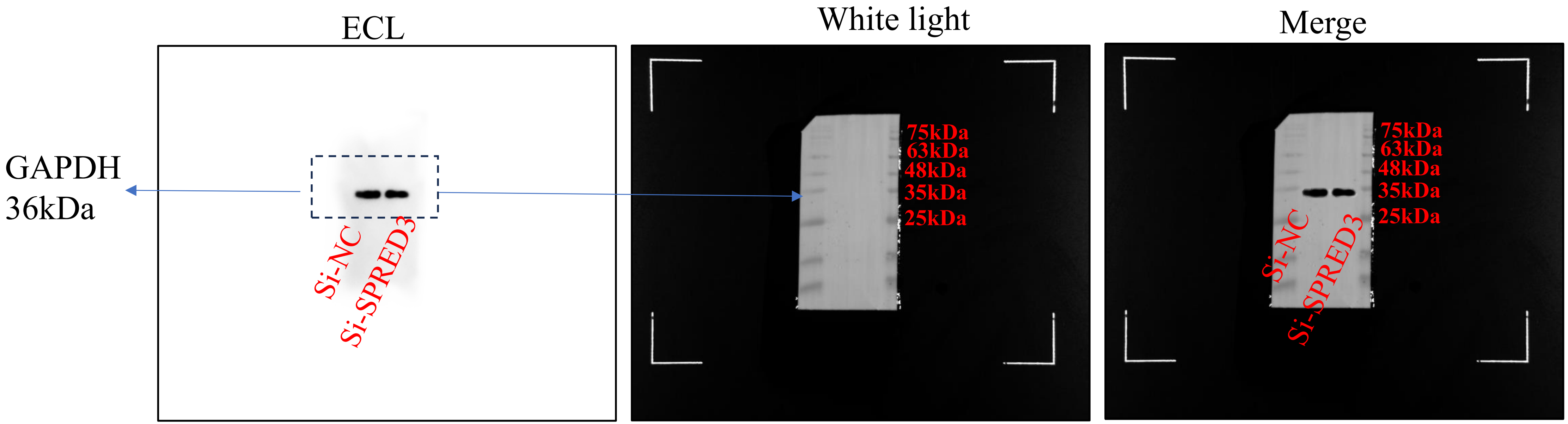

Multiple exposure images

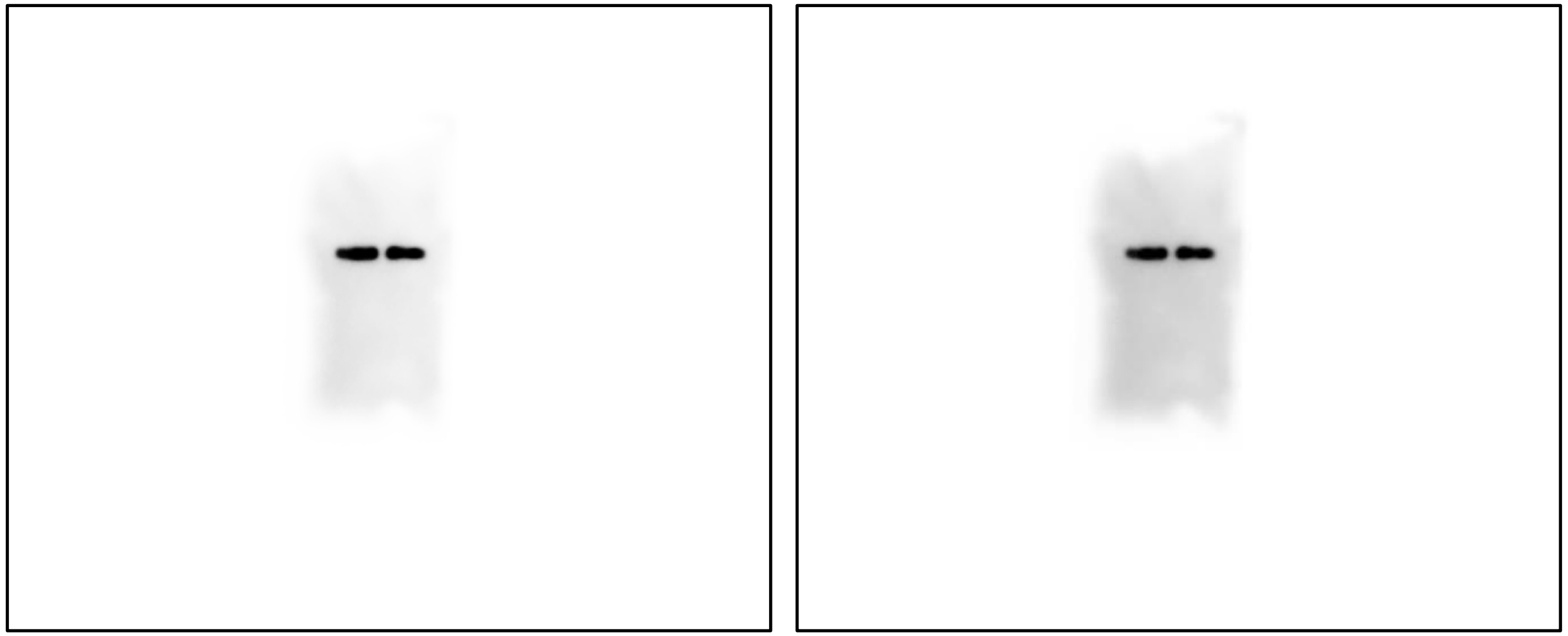

The primary images of the blots-Figure 5E—GAPDH

N87 cell line

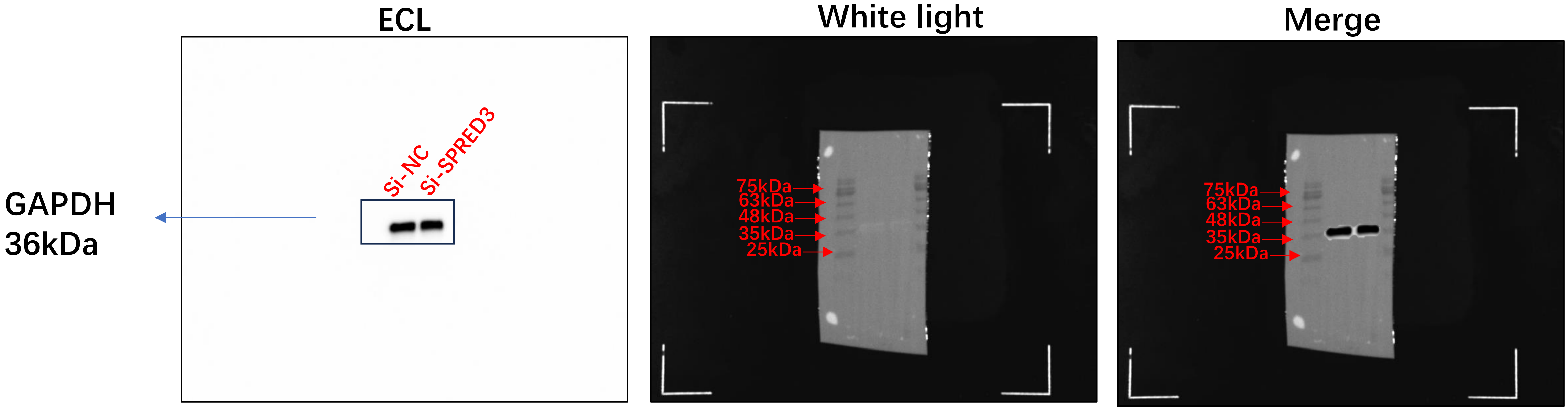

Multiple exposure images

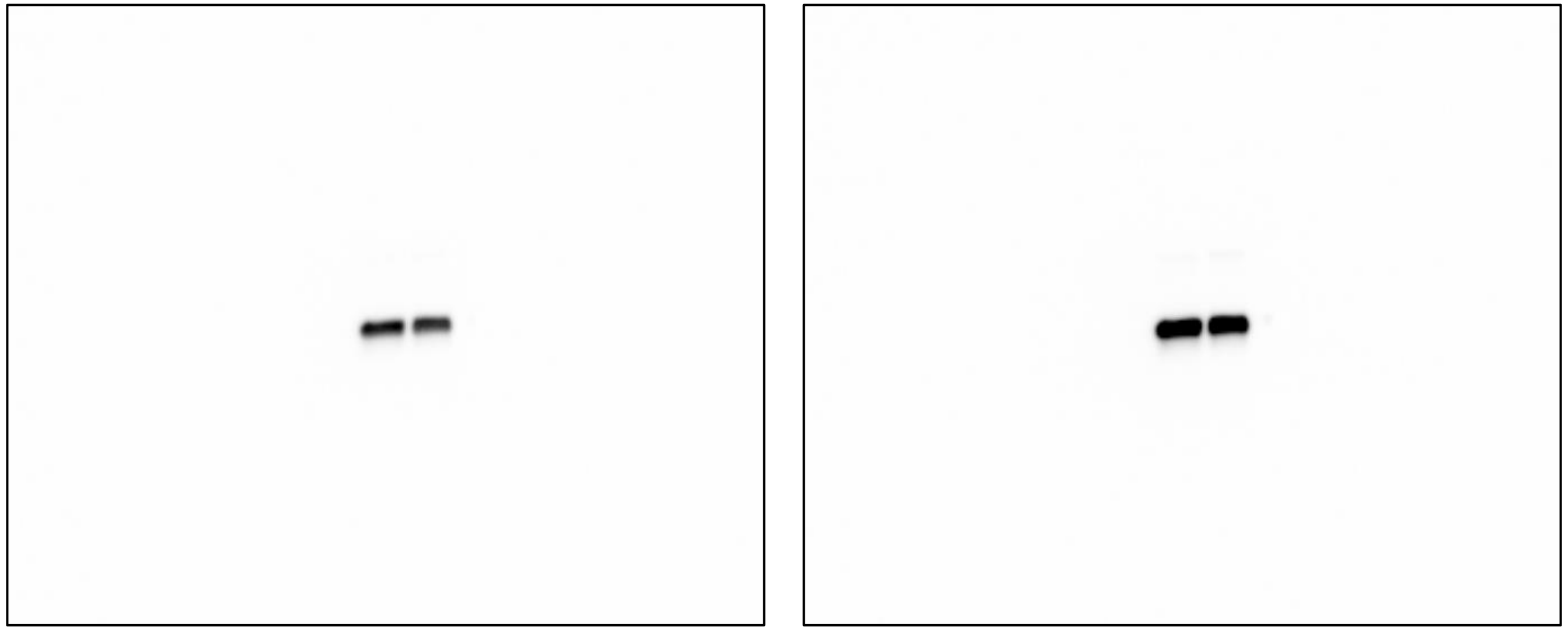

The primary images of the blots-Figure 5E—p38

N87 cell line

ECL

p38
38kDa

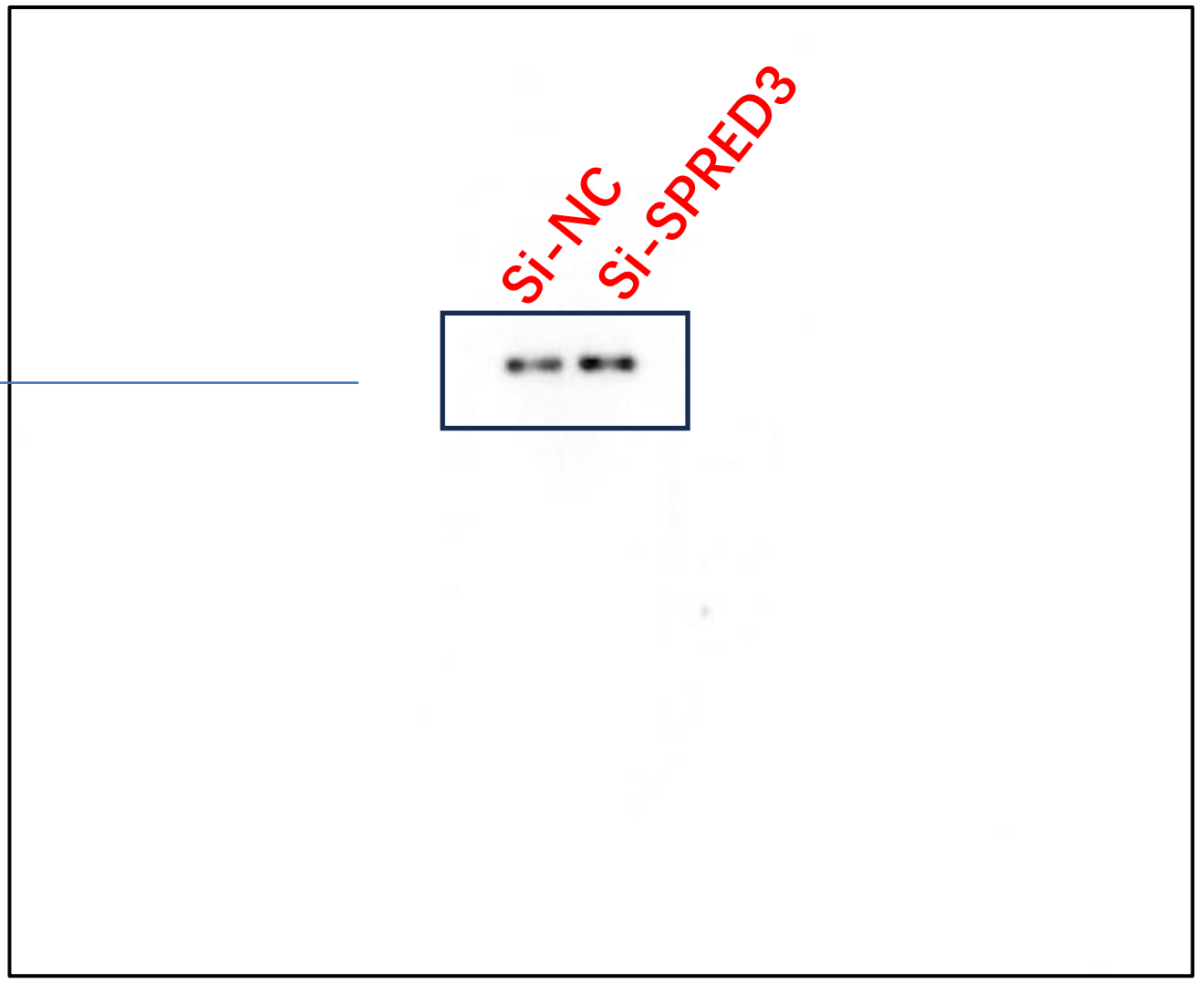

White light

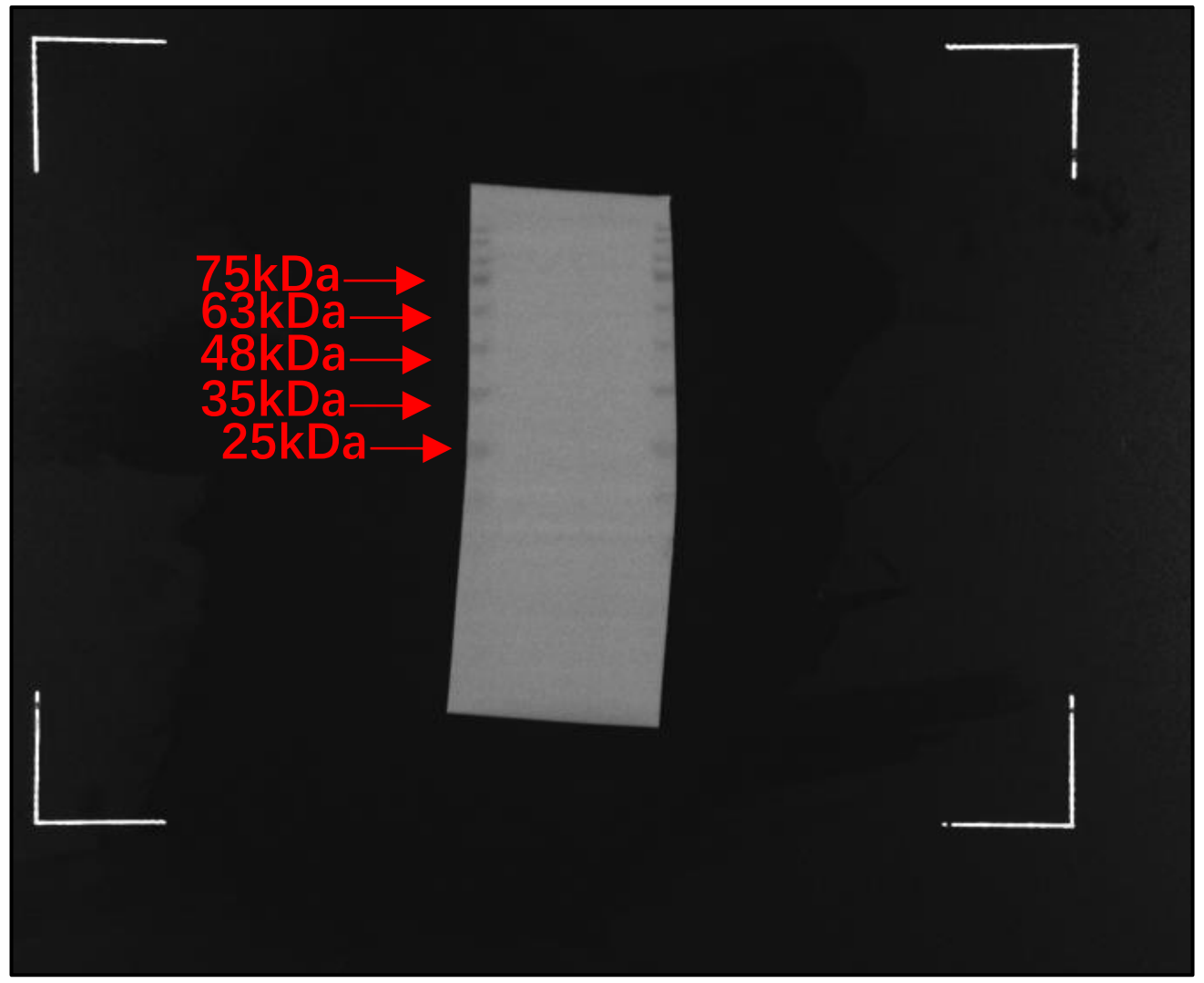

Merge

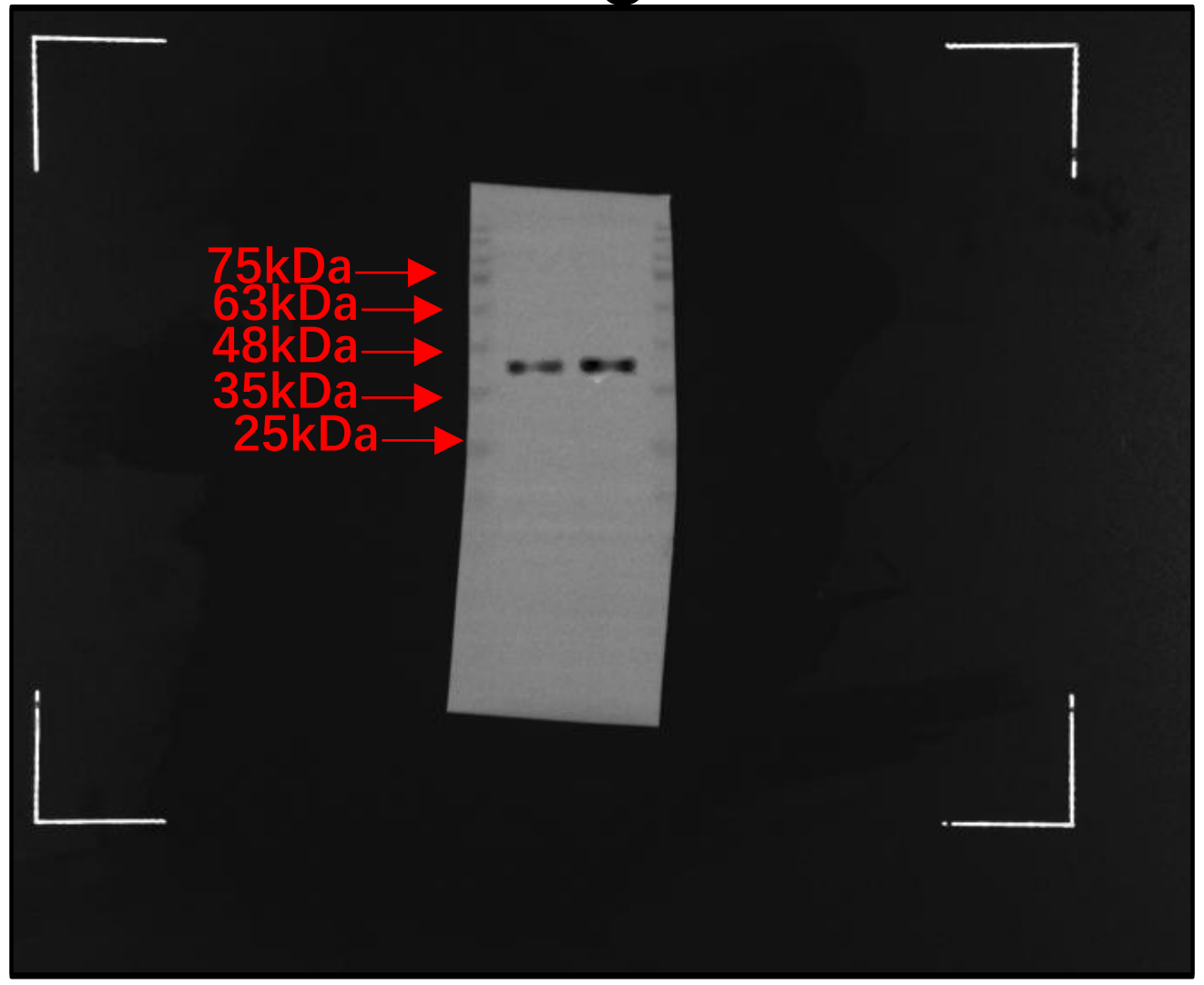

Multiple exposure images

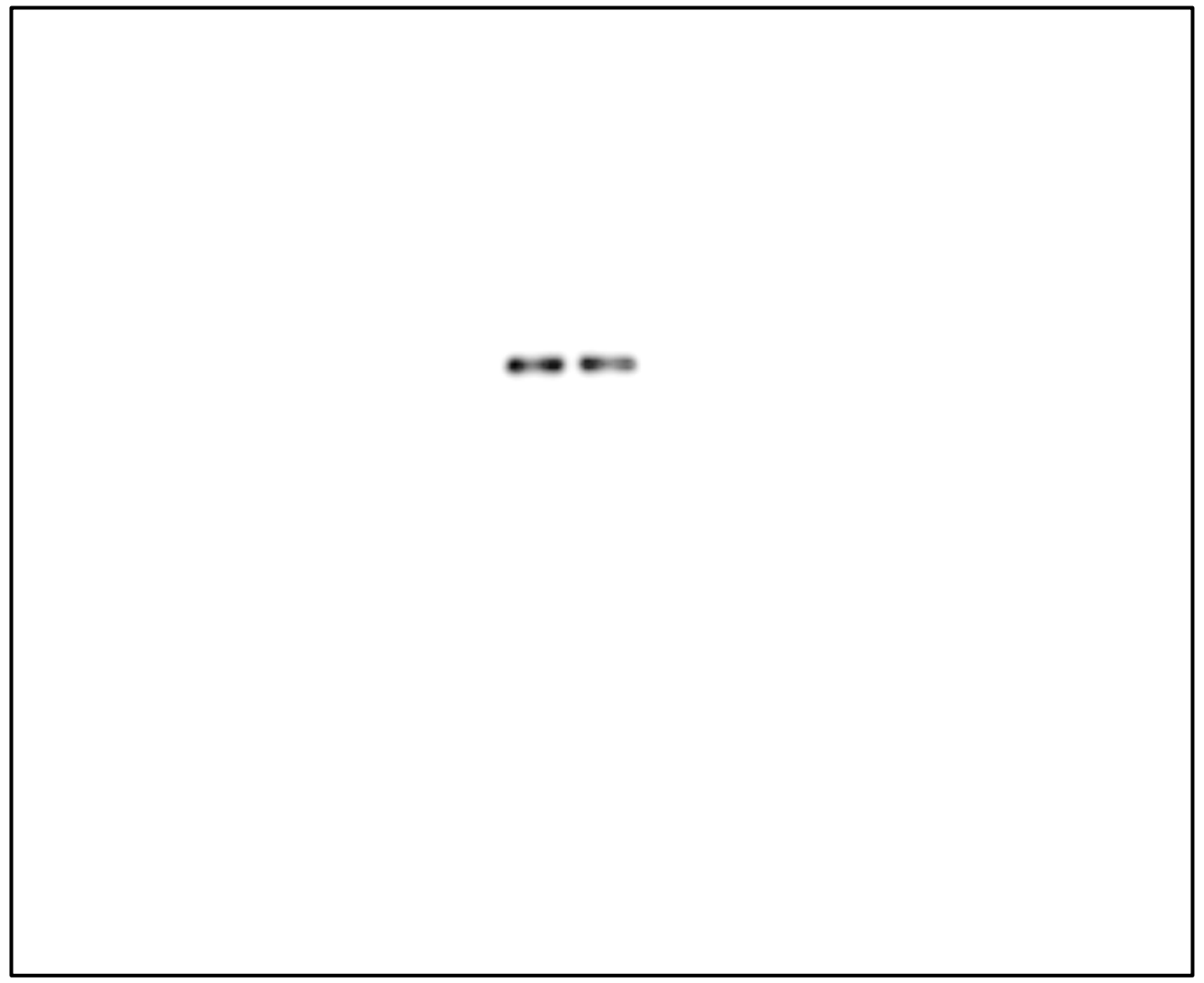

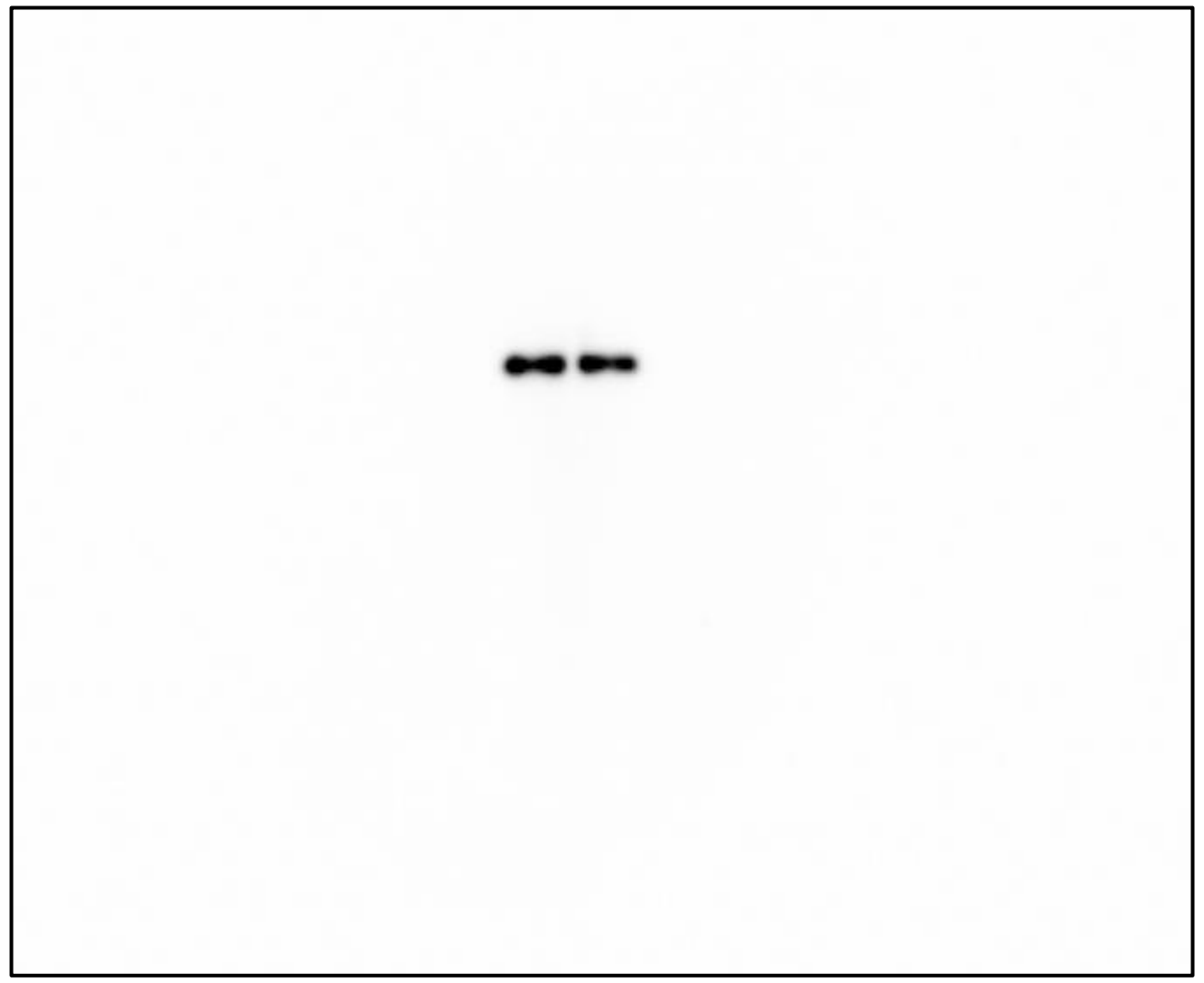

The primary images of the blots-Figure 5E—p-p38

N87 cell line

p-p38
38kDa

ECL

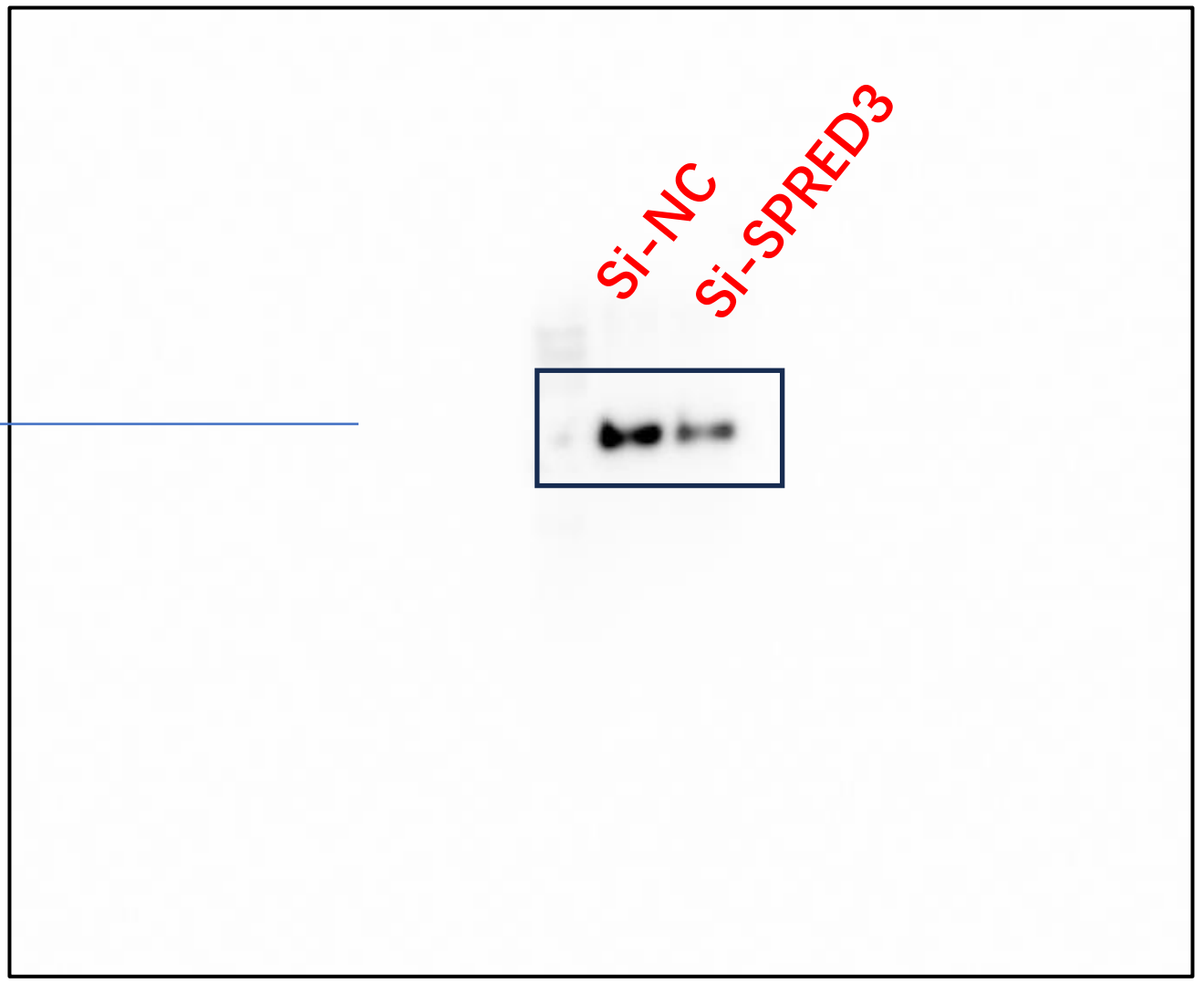

White light

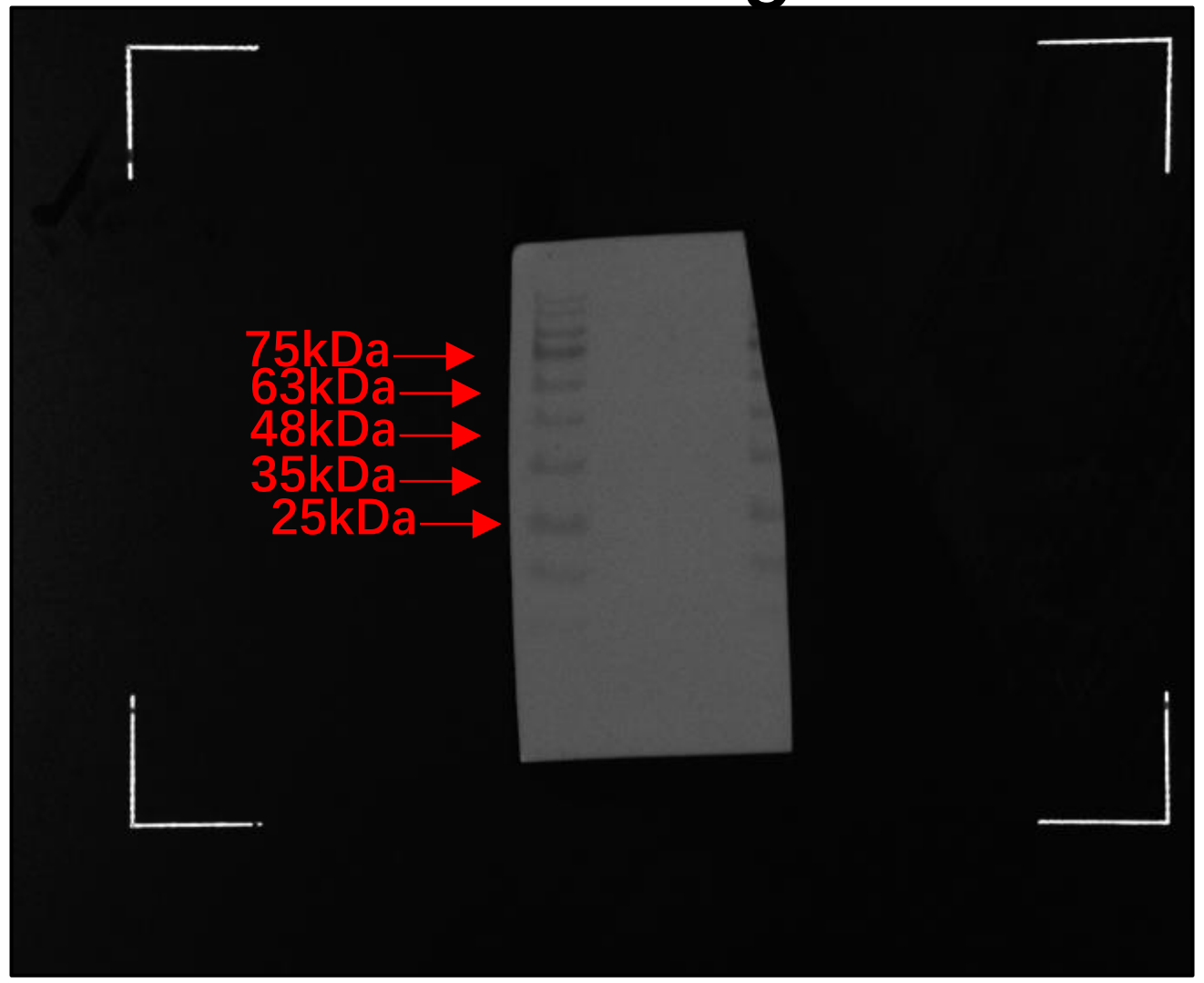

Merge

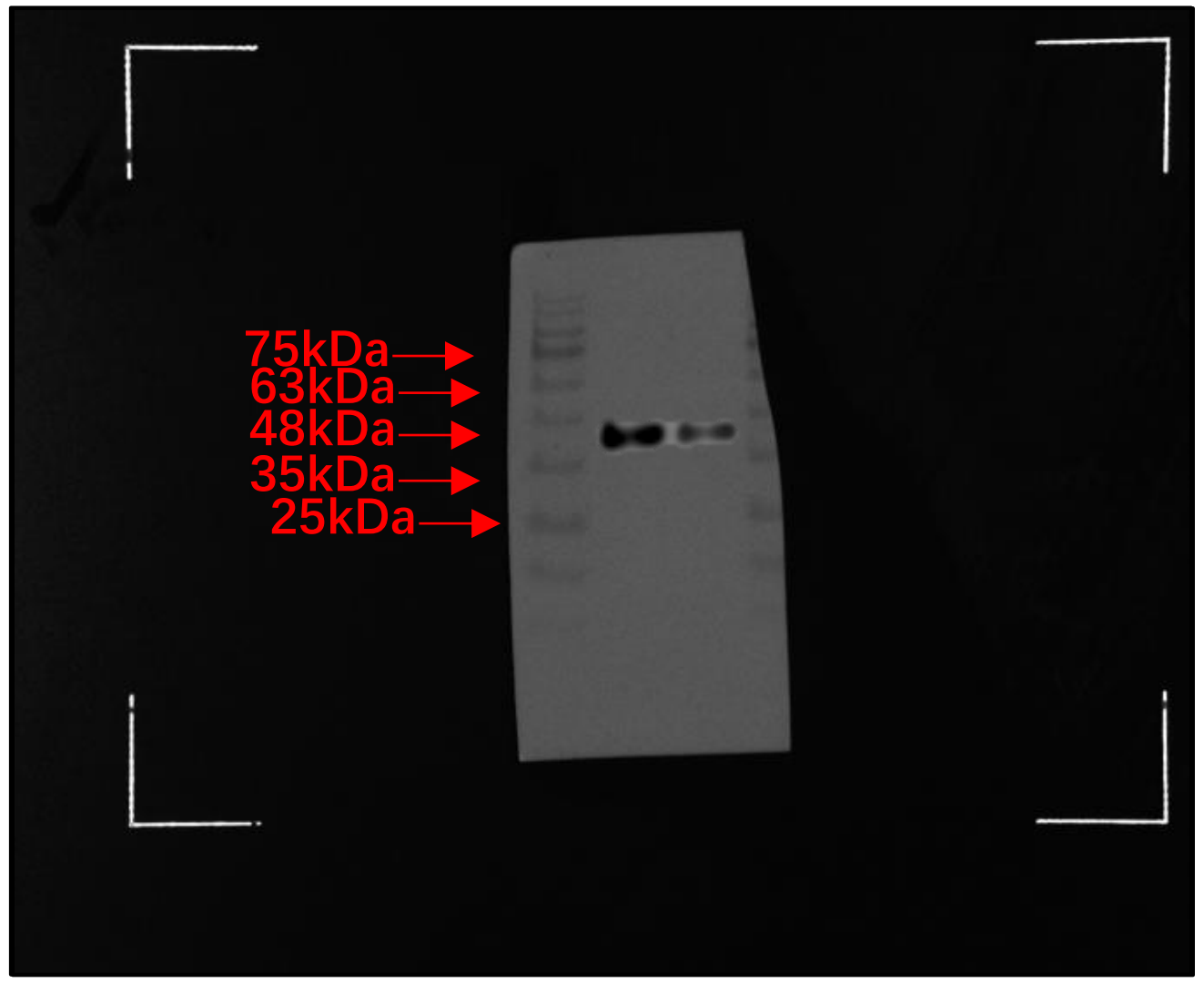

Multiple exposure images

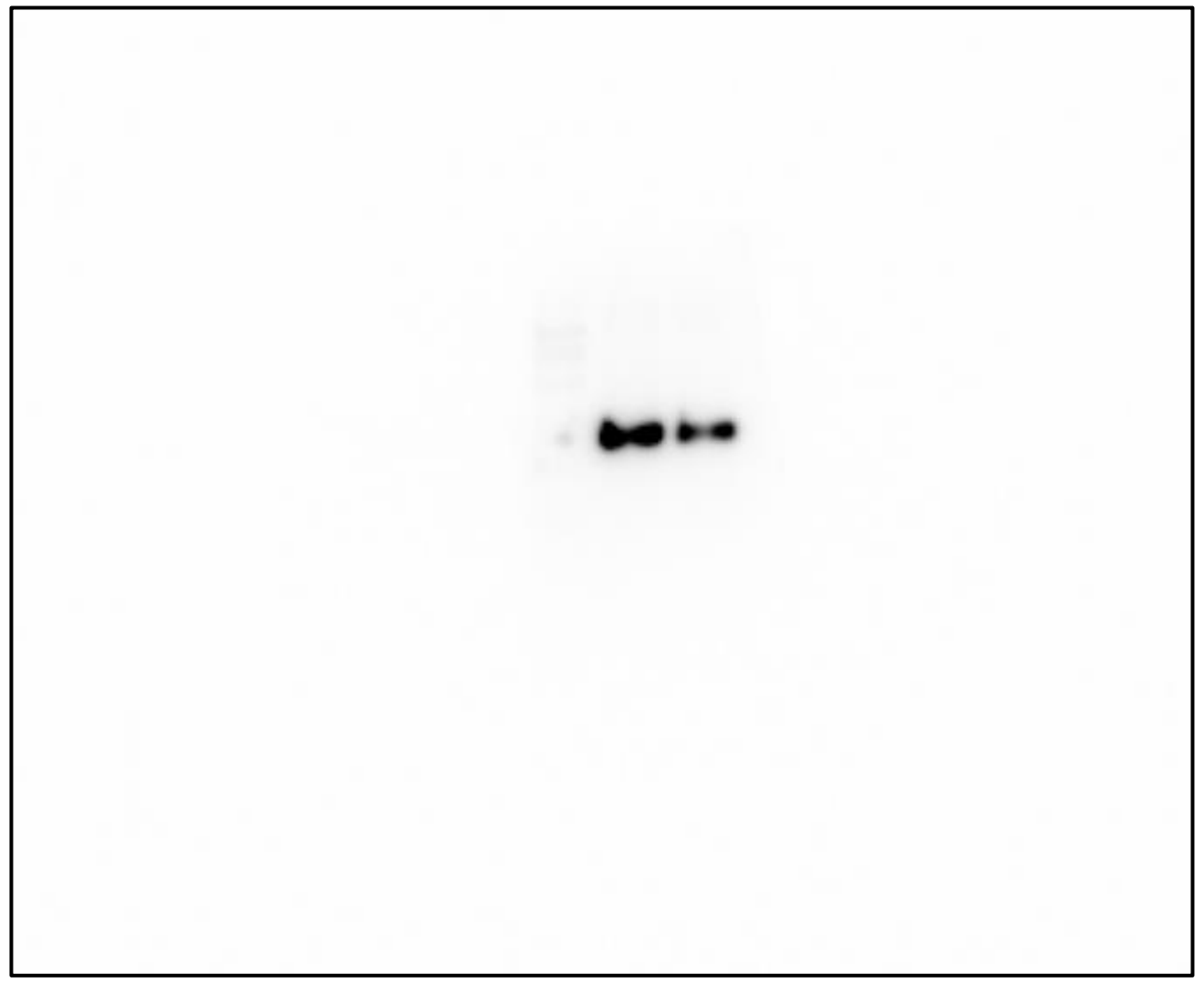

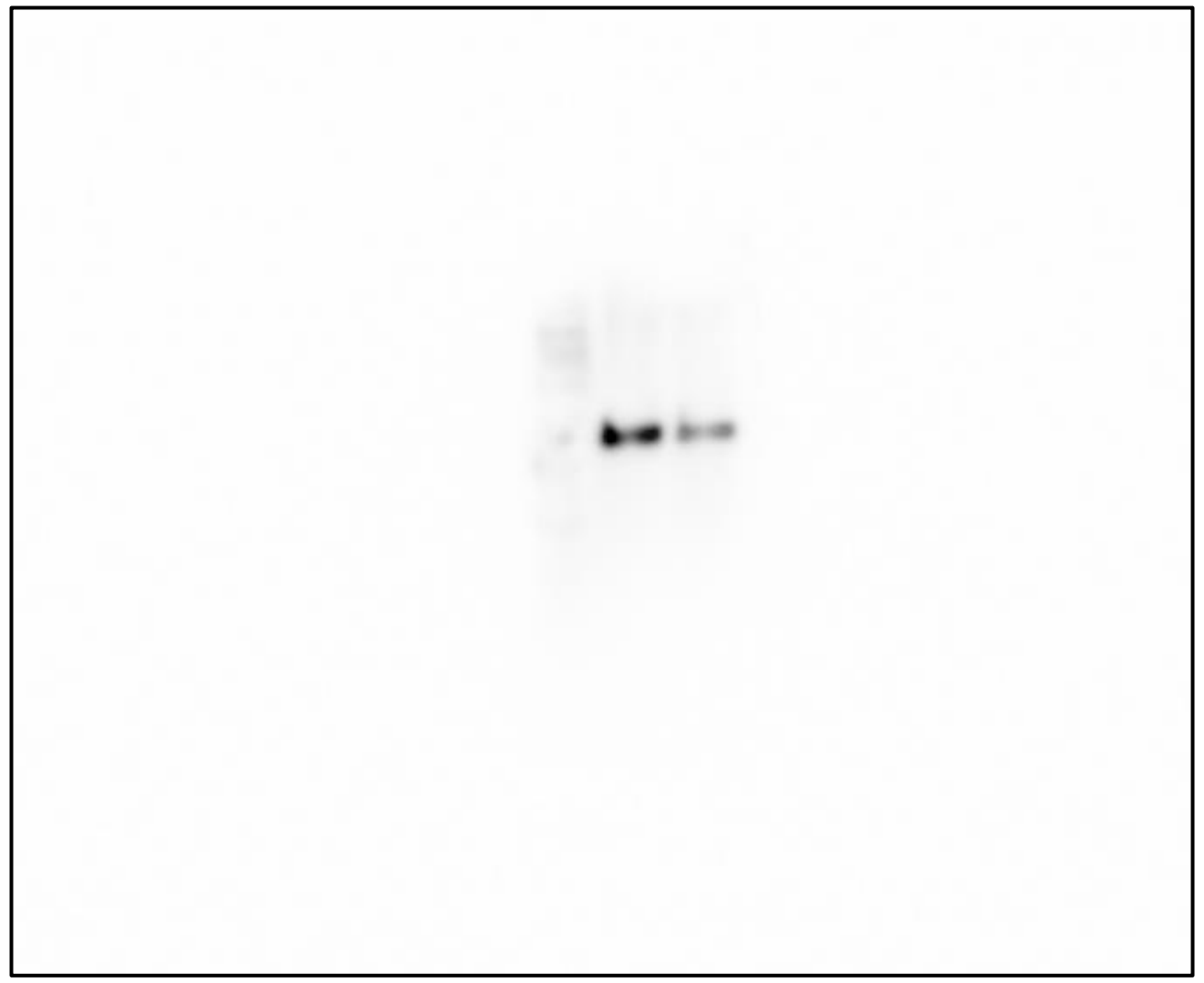

The primary images of the blots-Figure 5E—p65

N87 cell line

p65
65kDa

ECL

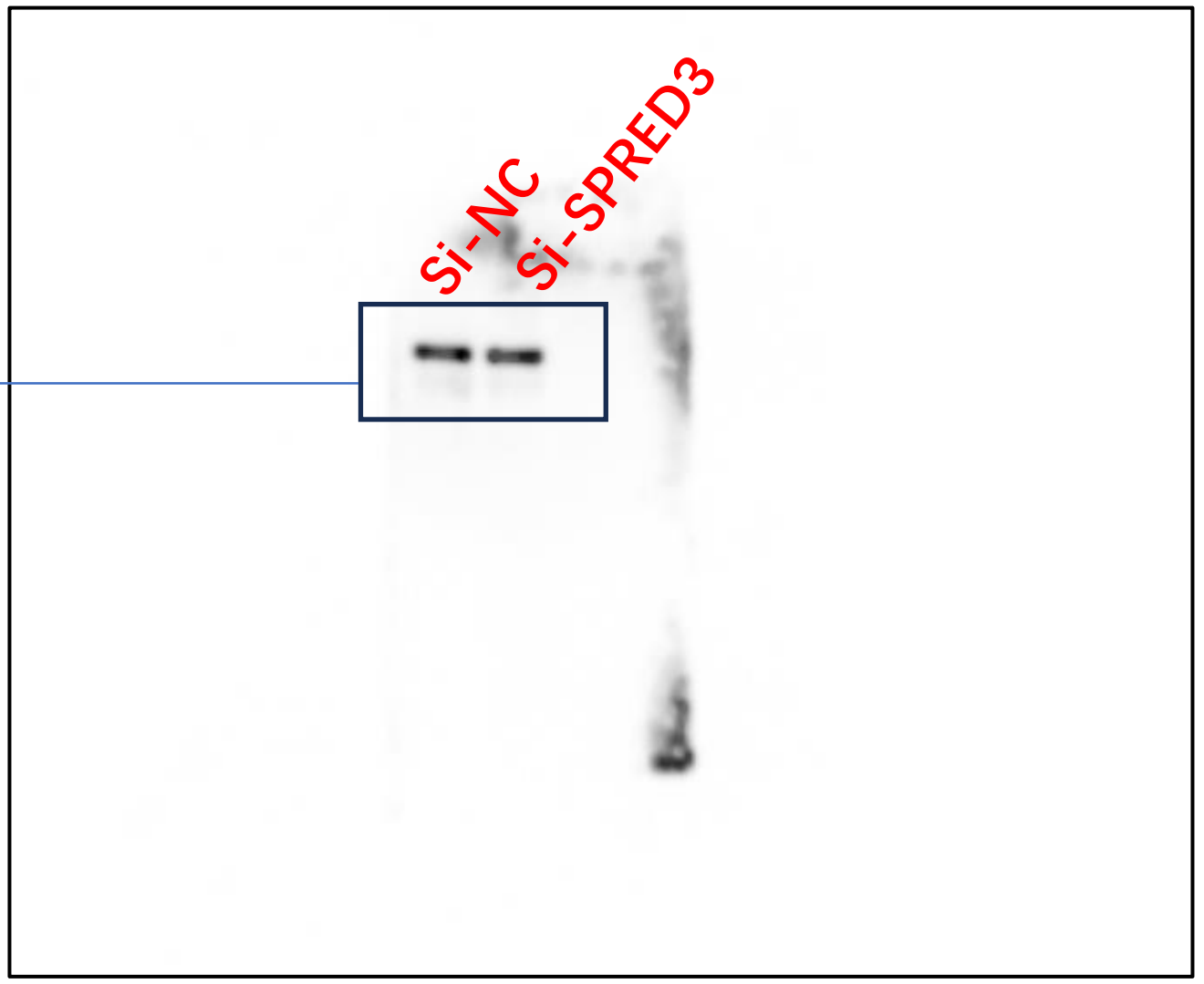

White light

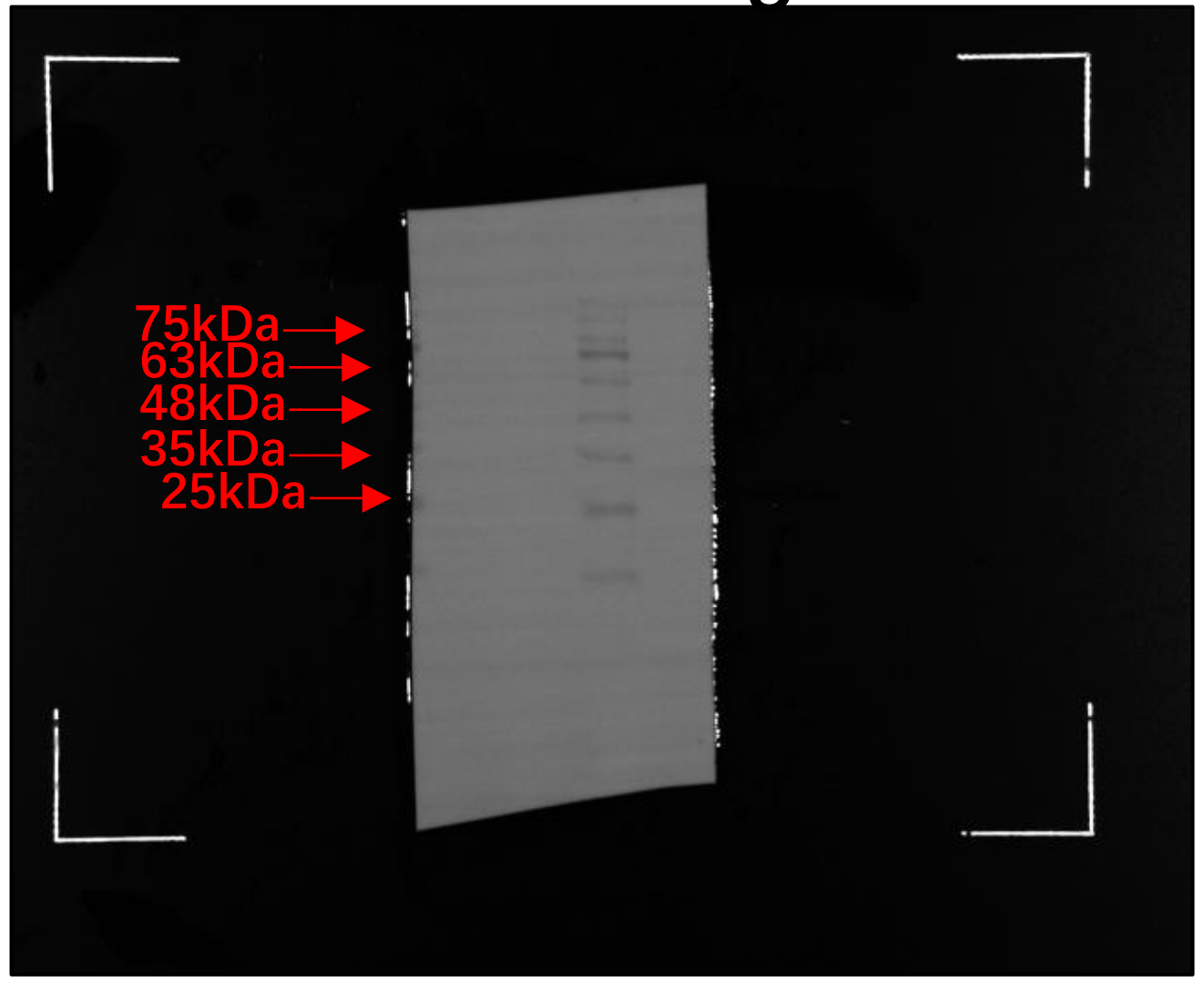

Merge

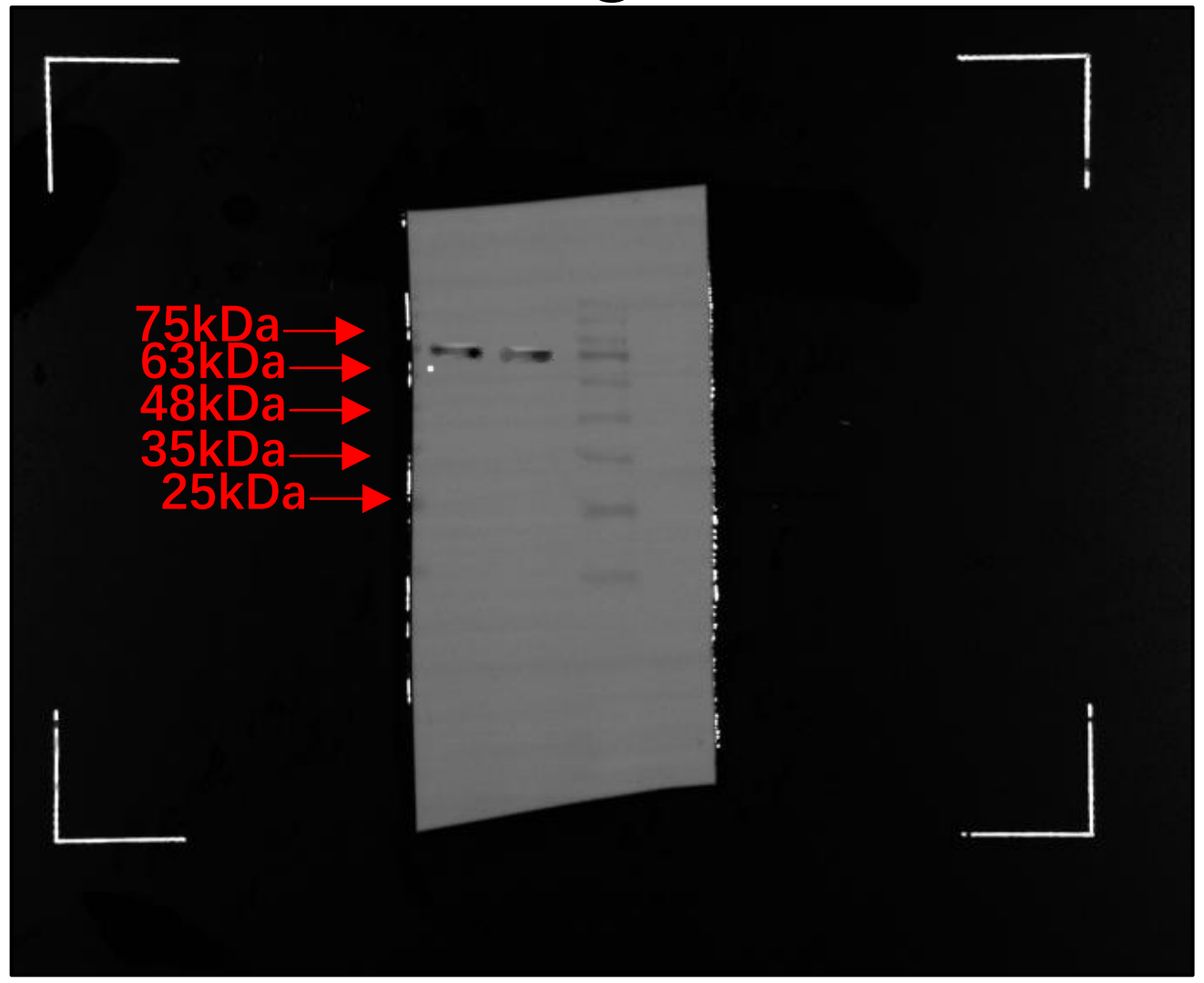

Multiple exposure images

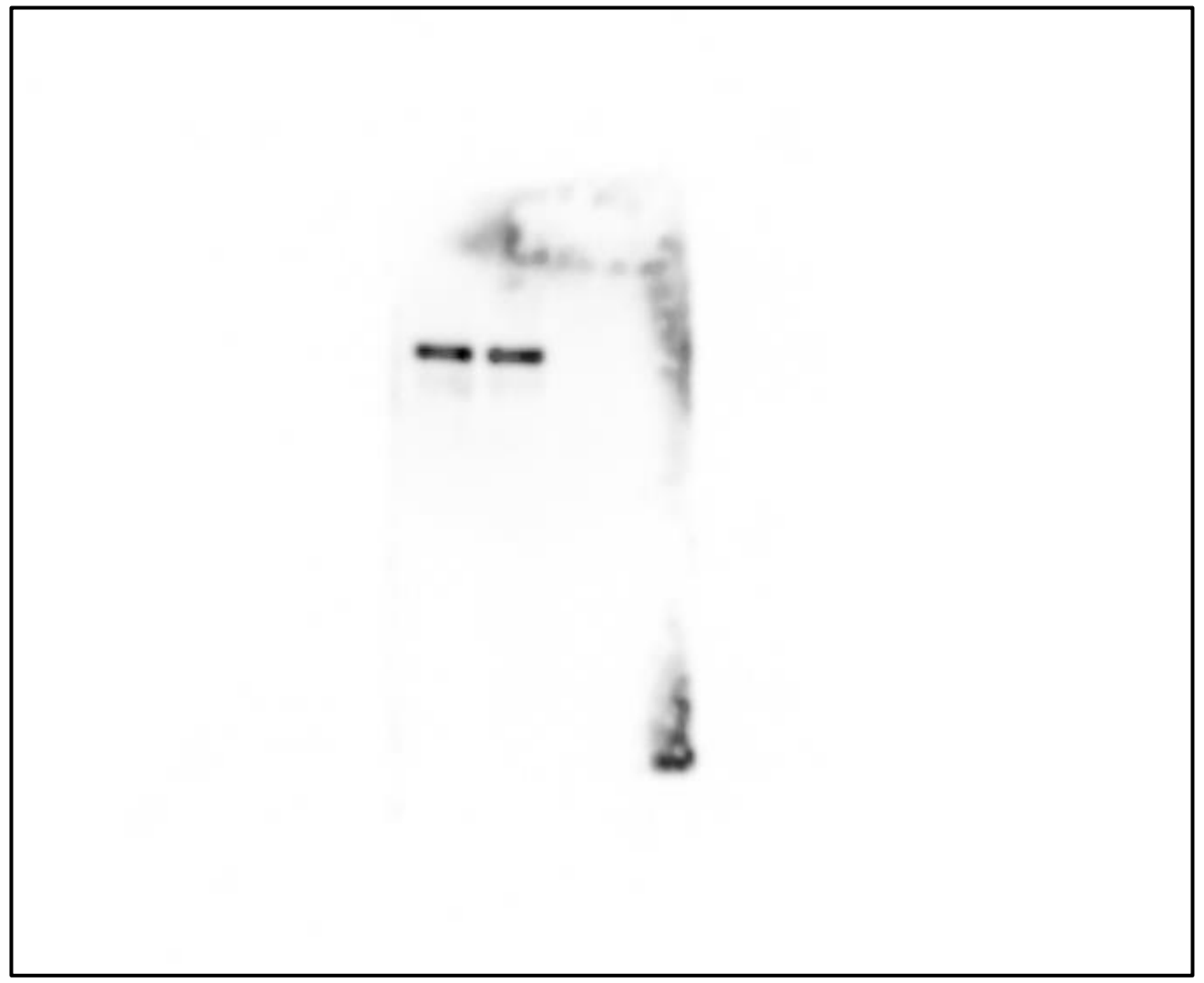

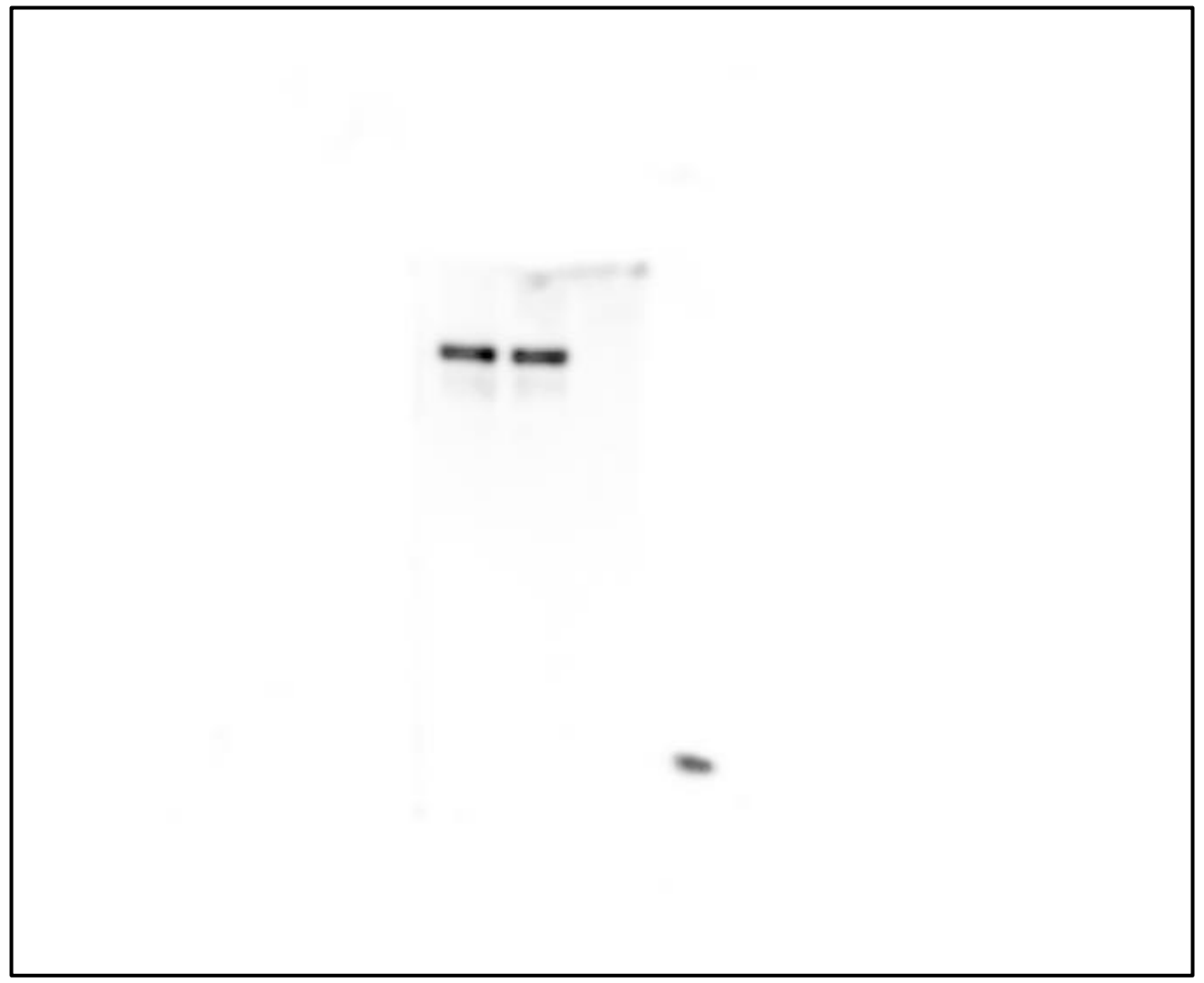

The primary images of the blots-Figure 5E—p-p65

N87 cell line

ECL

p-p65
65kDa

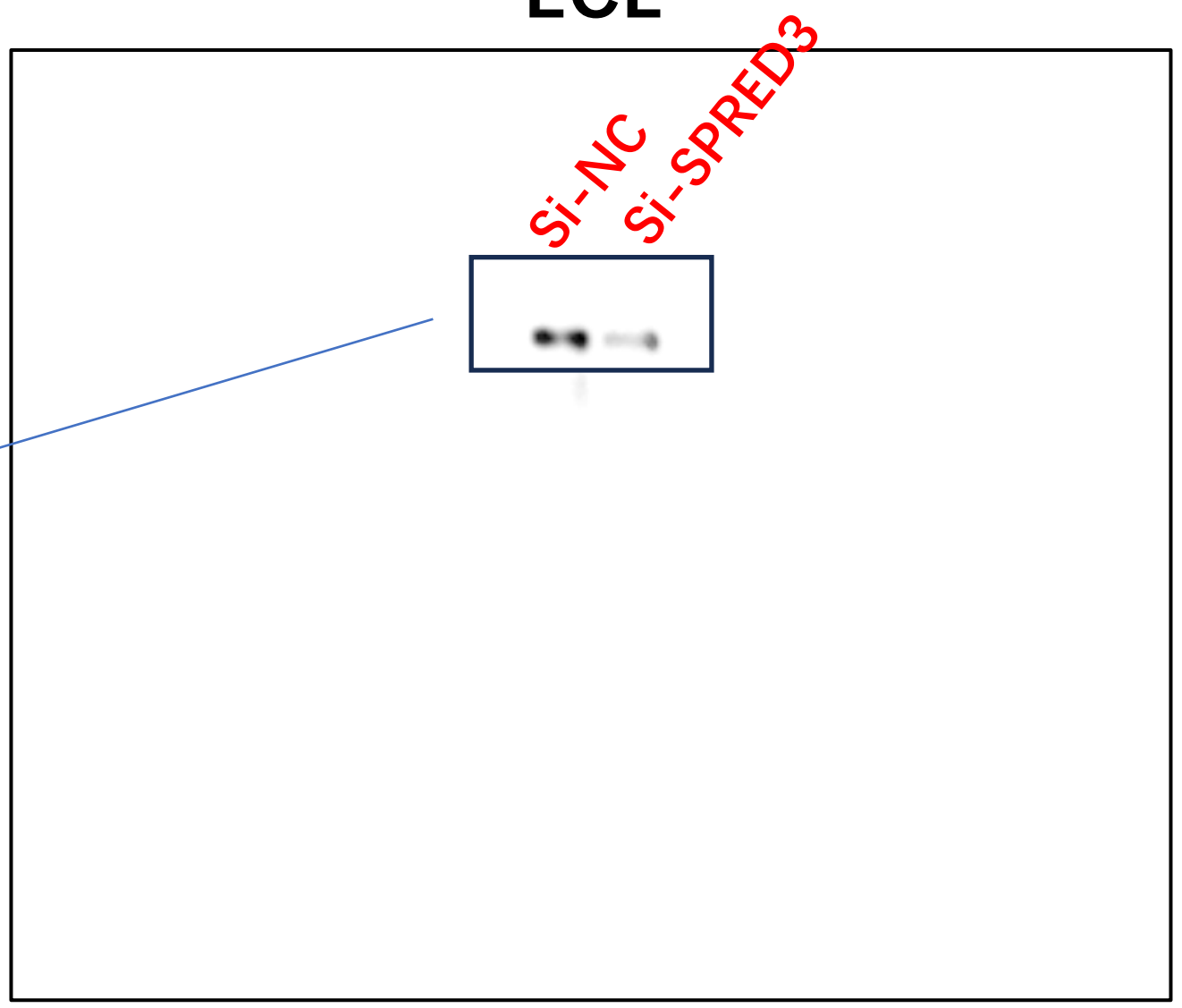

White light

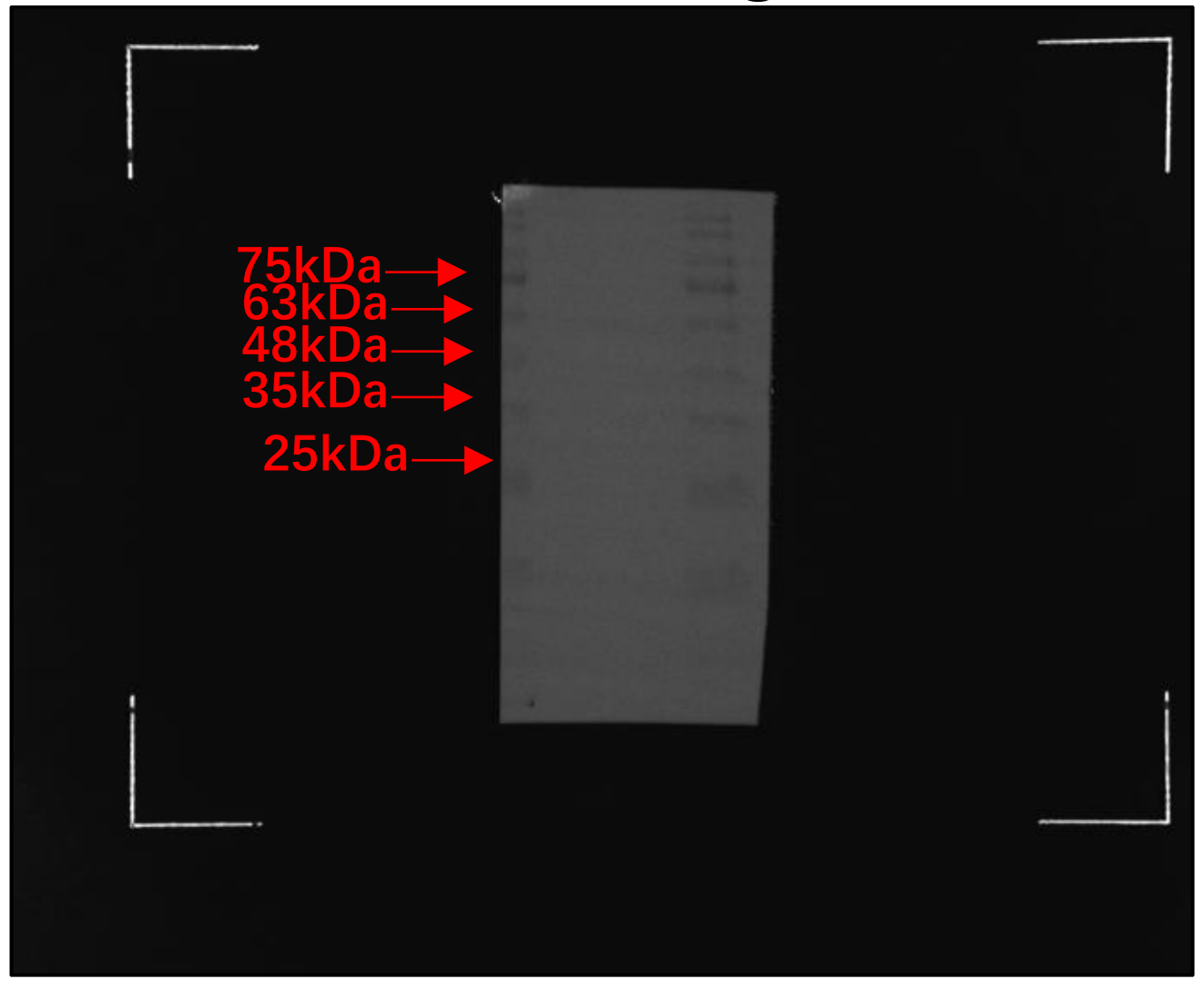

Merge

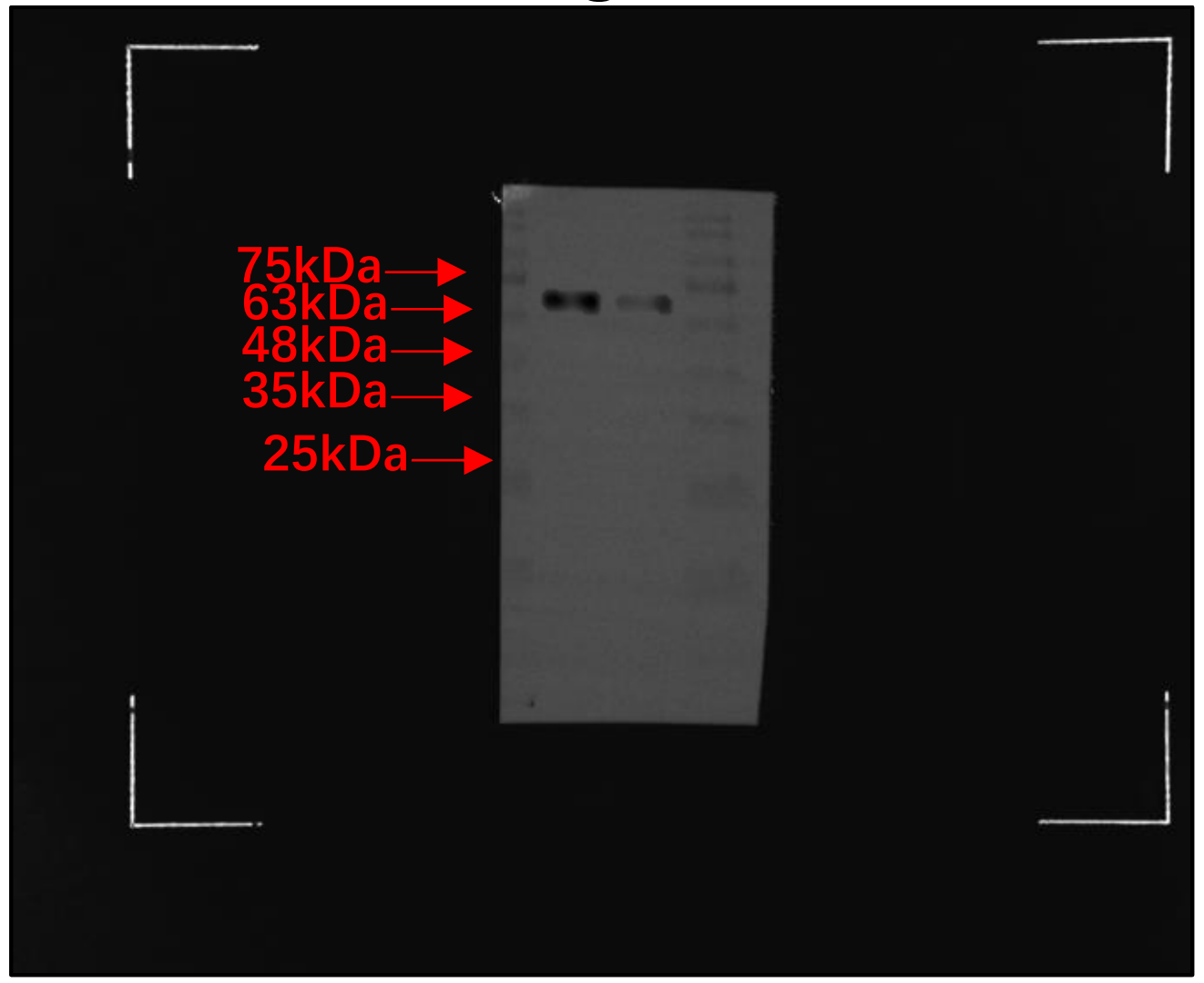

Multiple exposure images

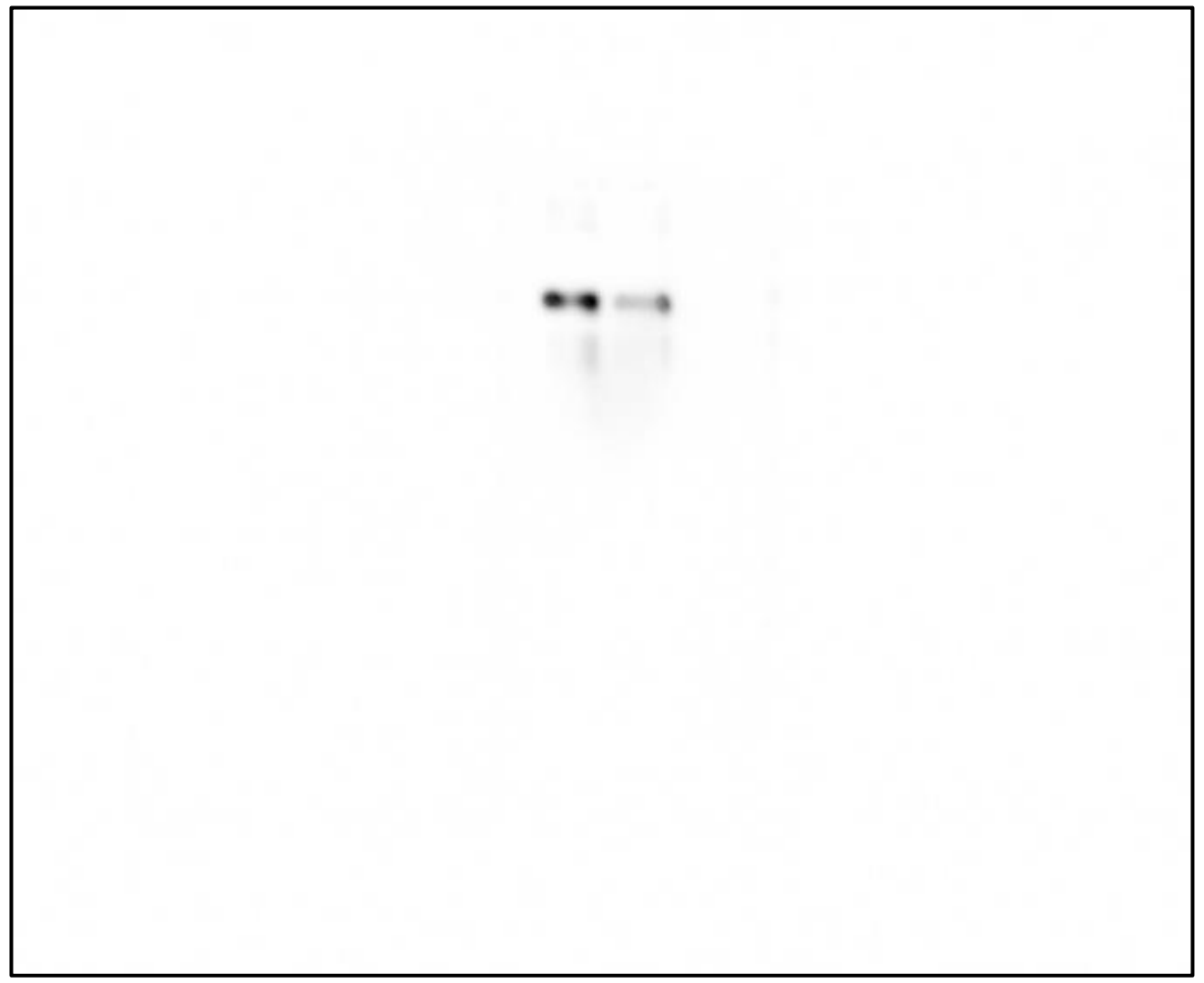

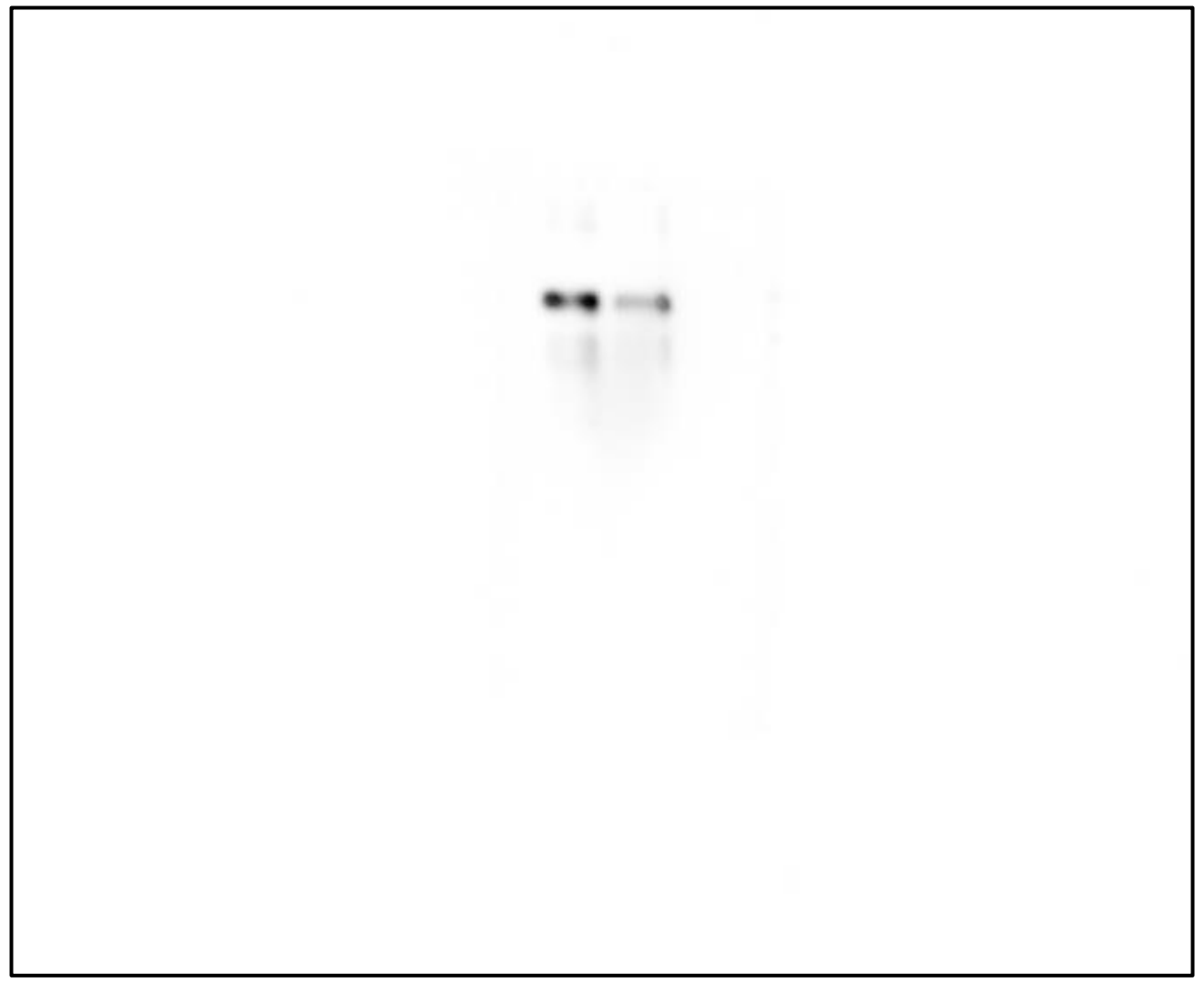

The primary images of the blots-Figure 5F—GAPDH

AGS and N87 cell lines

ECL

GAPDH
36kDa

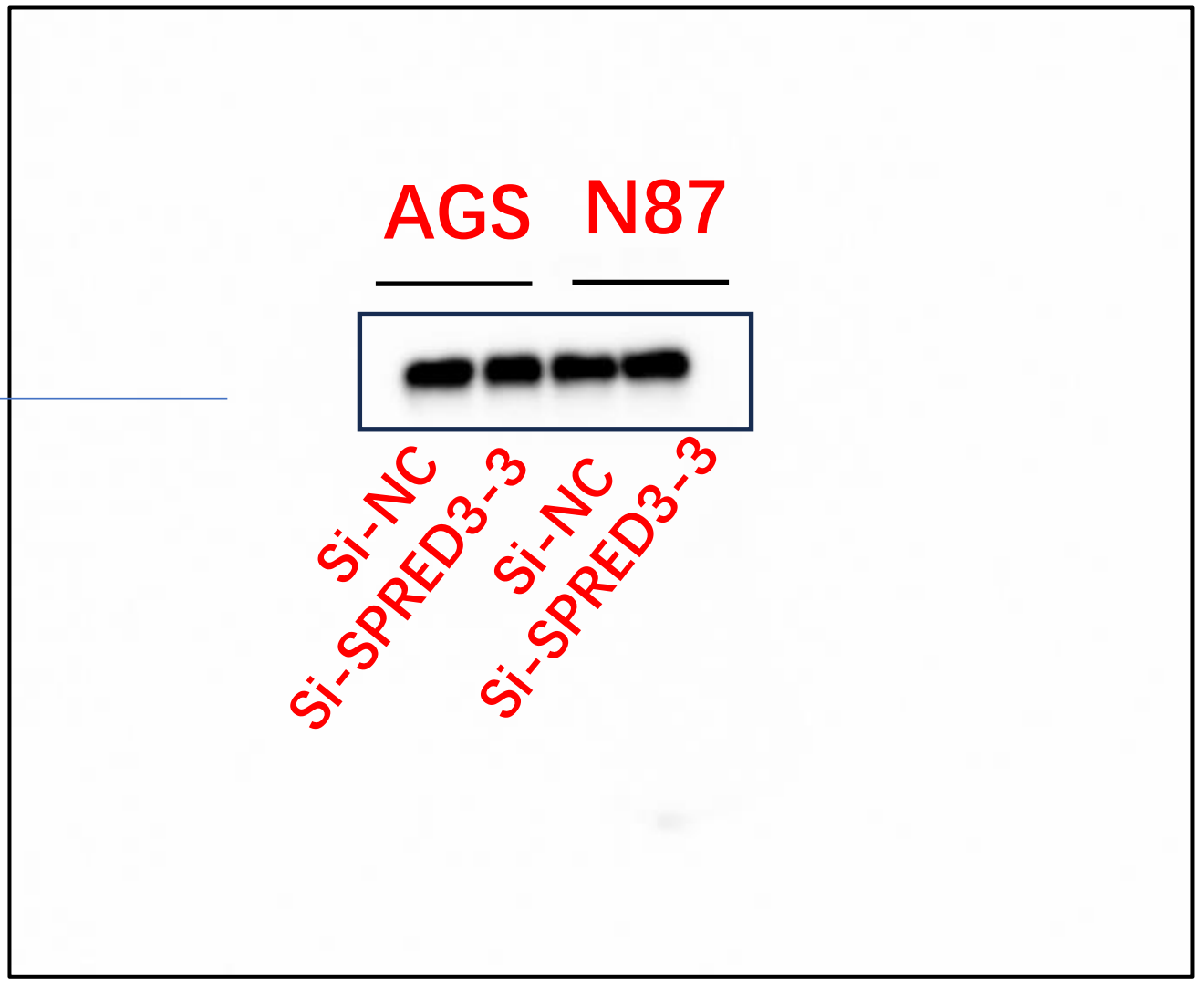

White light

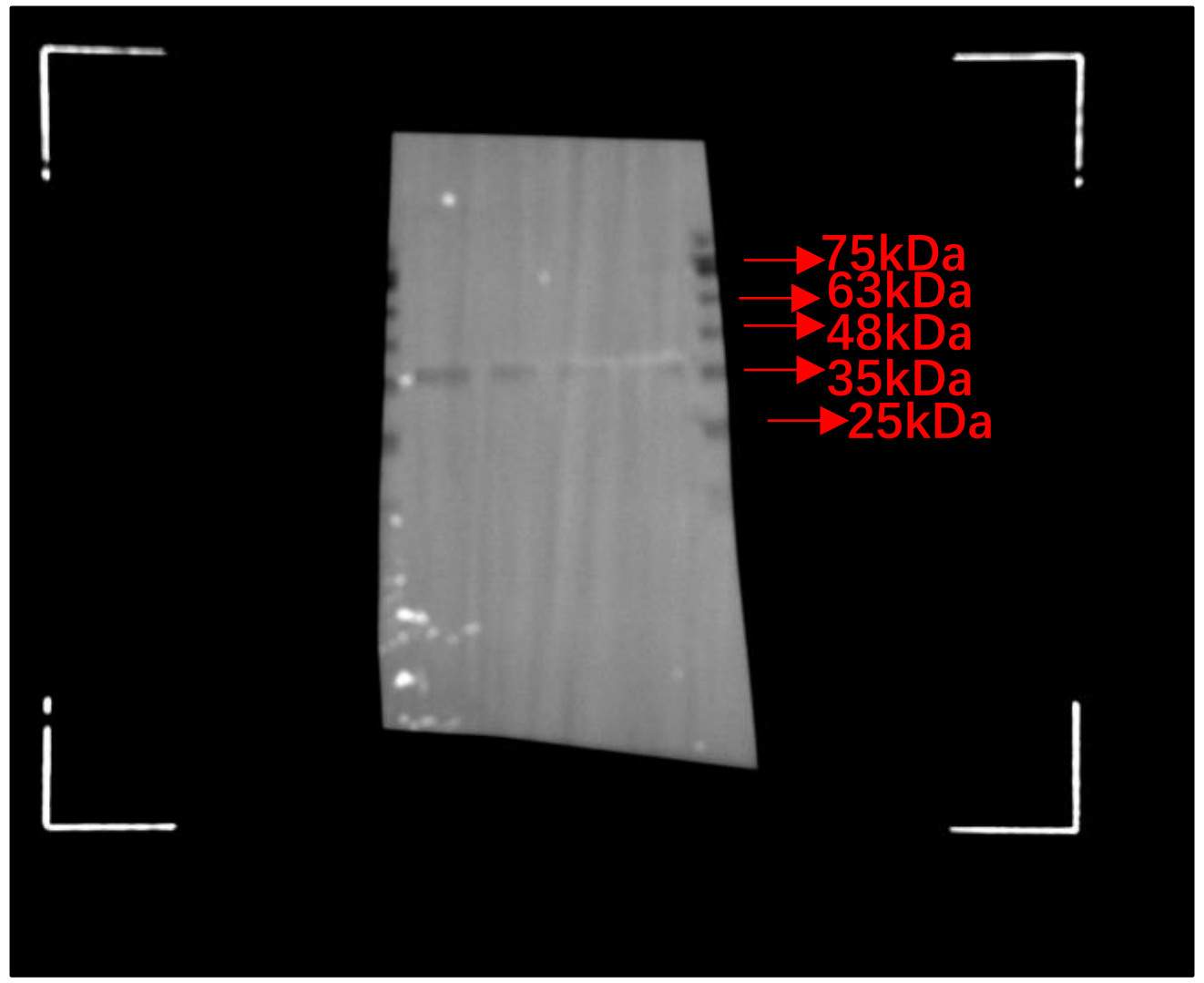

Merge

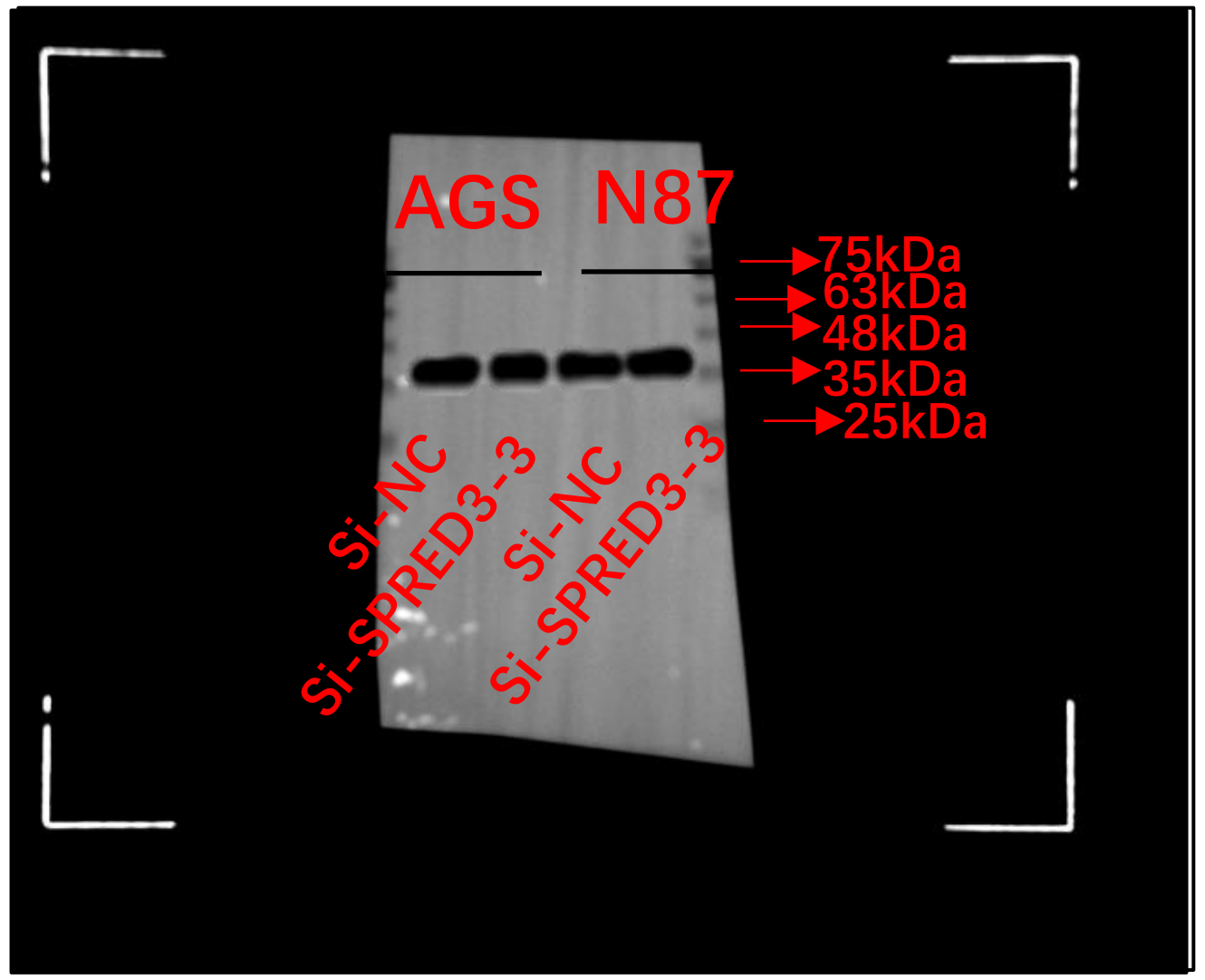

Multiple exposure images

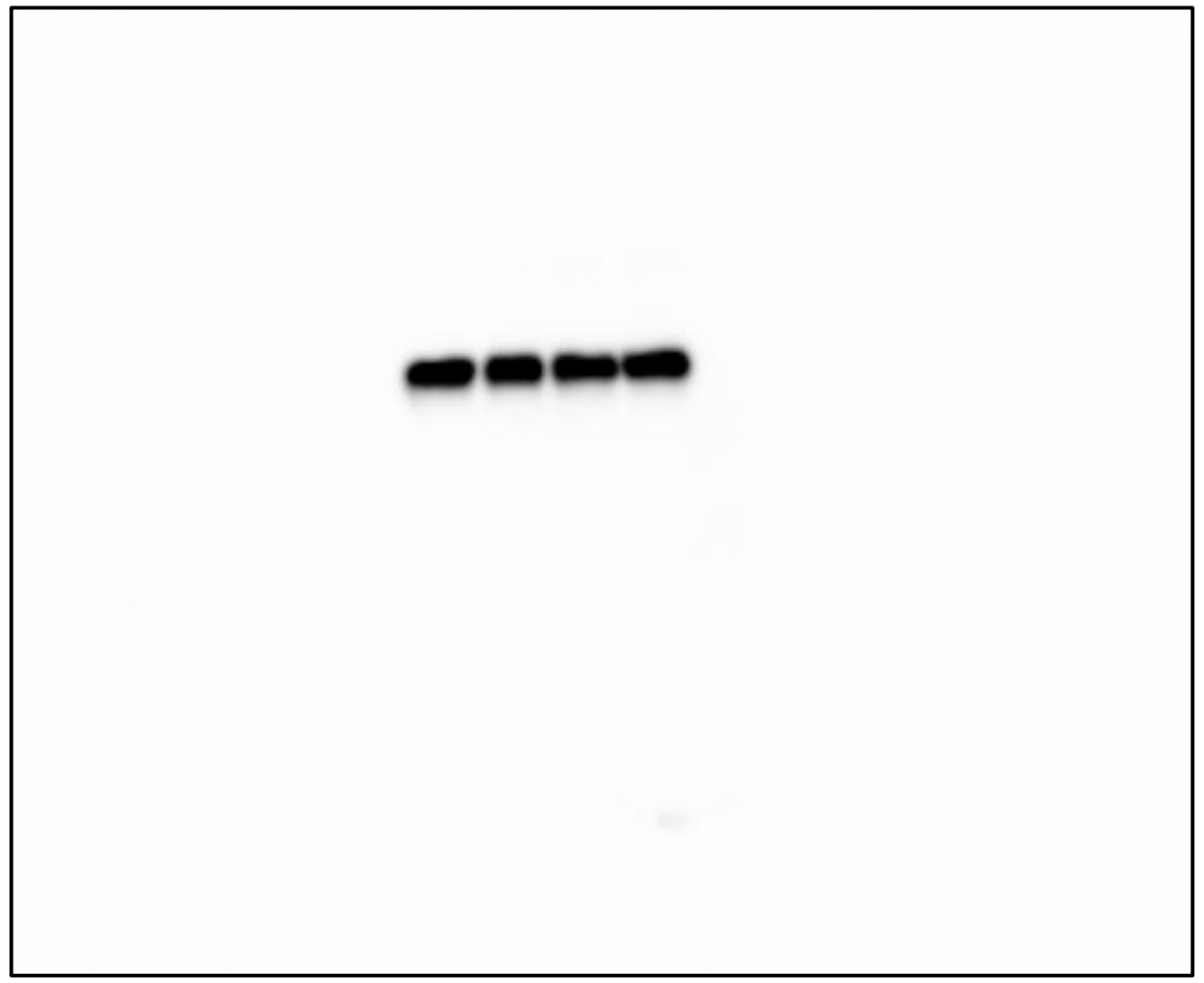

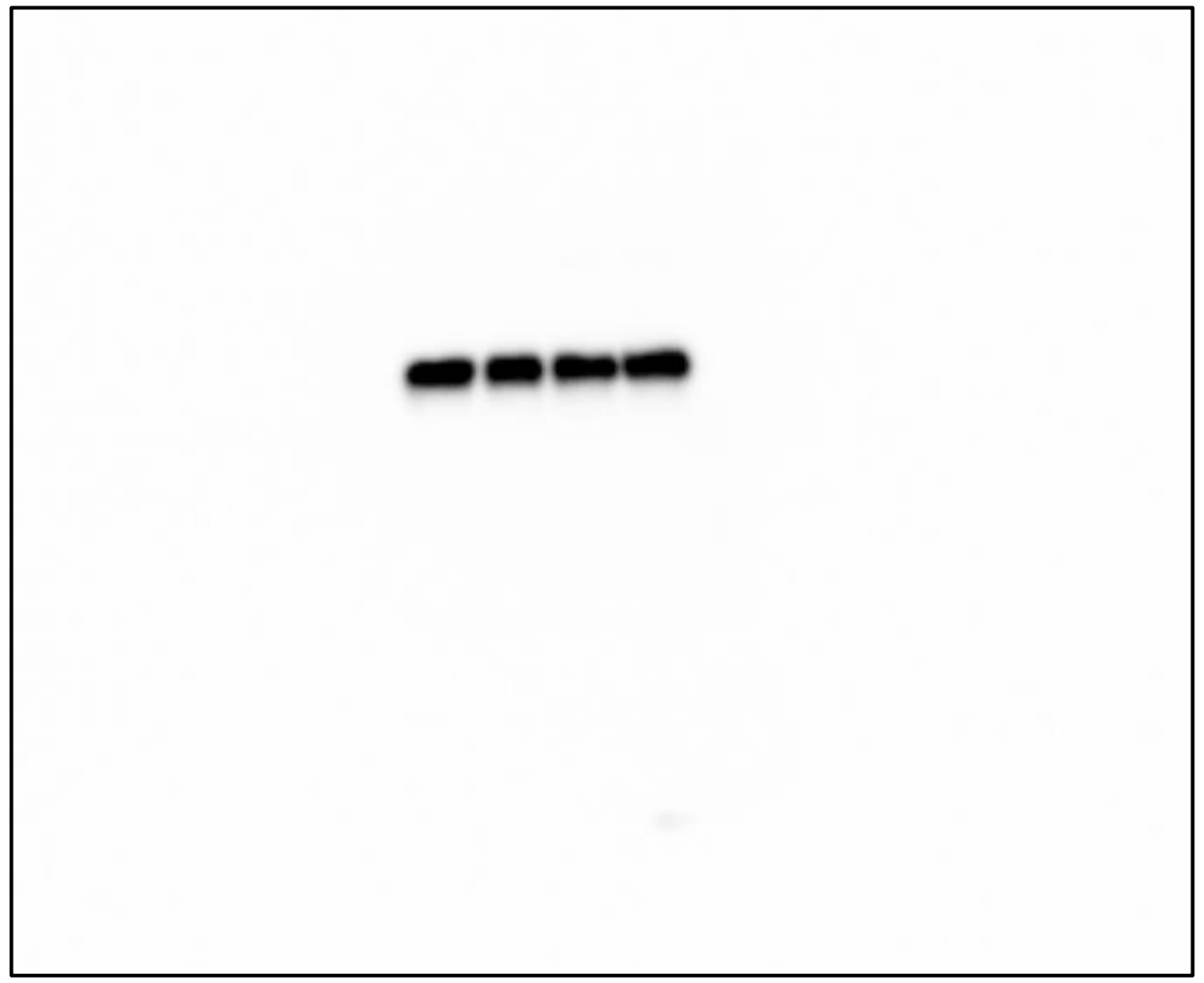

The primary images of the blots-Figure 5F—MKK3/6

AGS and N87 cell lines

ECL

MKK3/6
35kDa

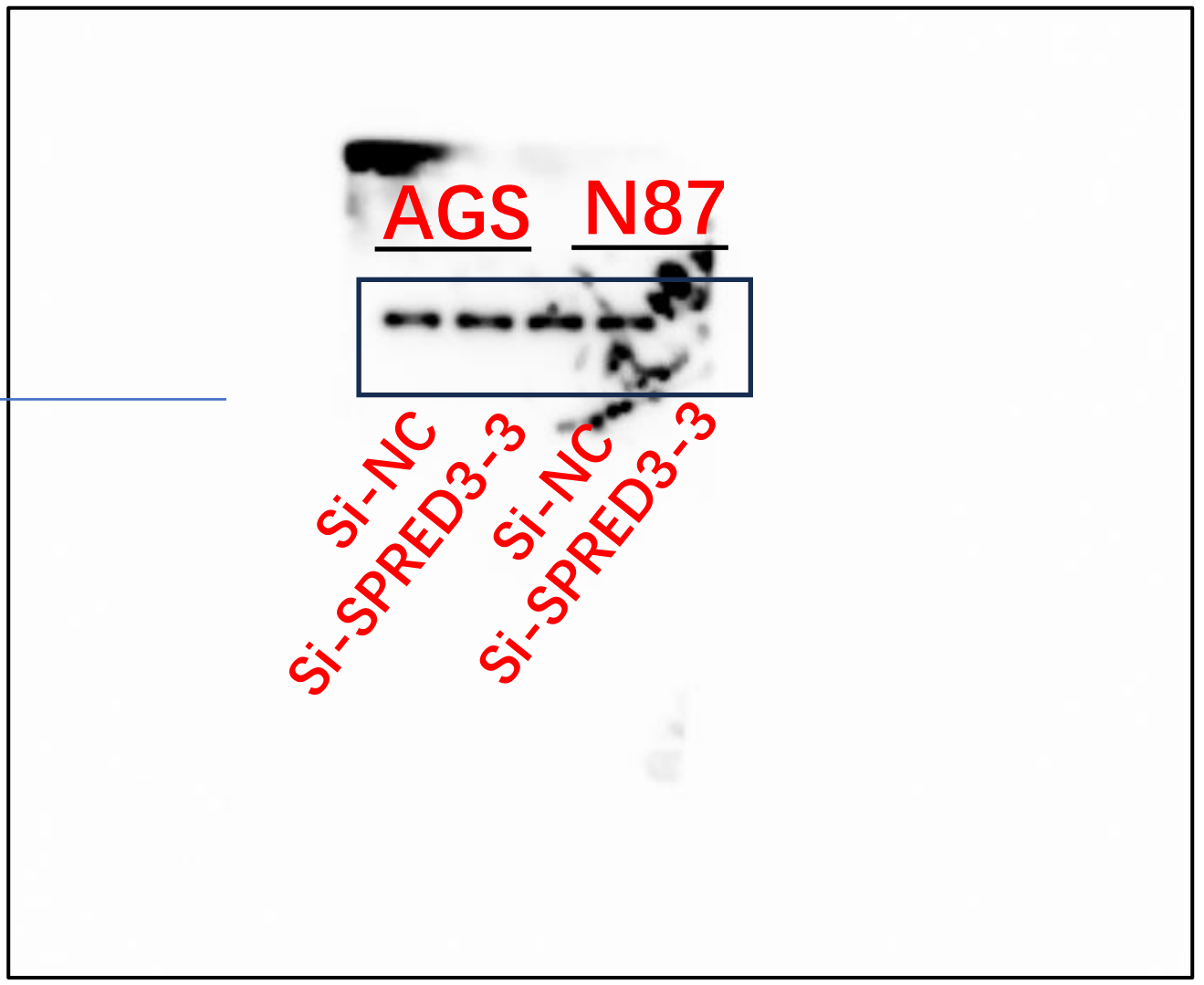

White light

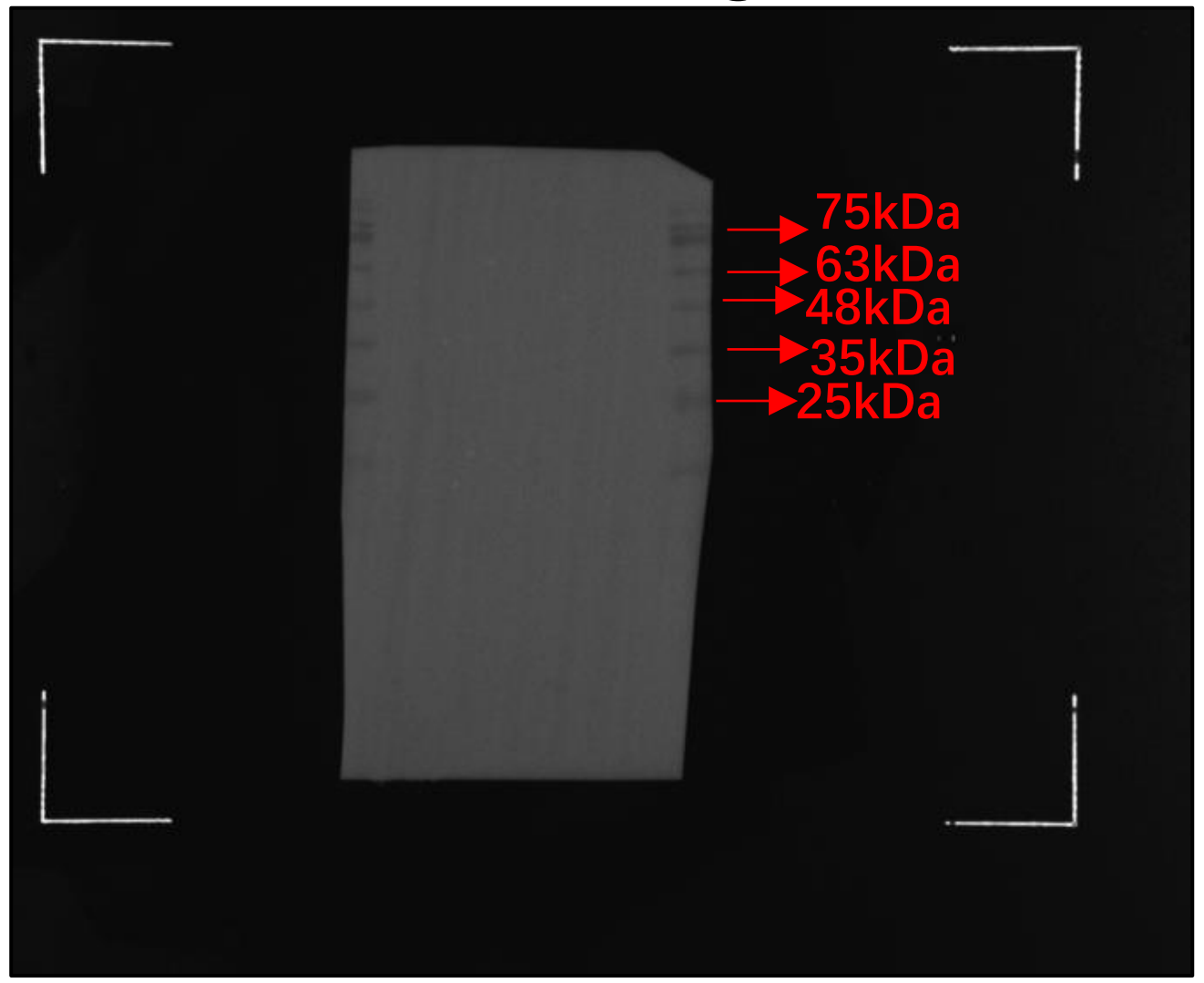

Merge

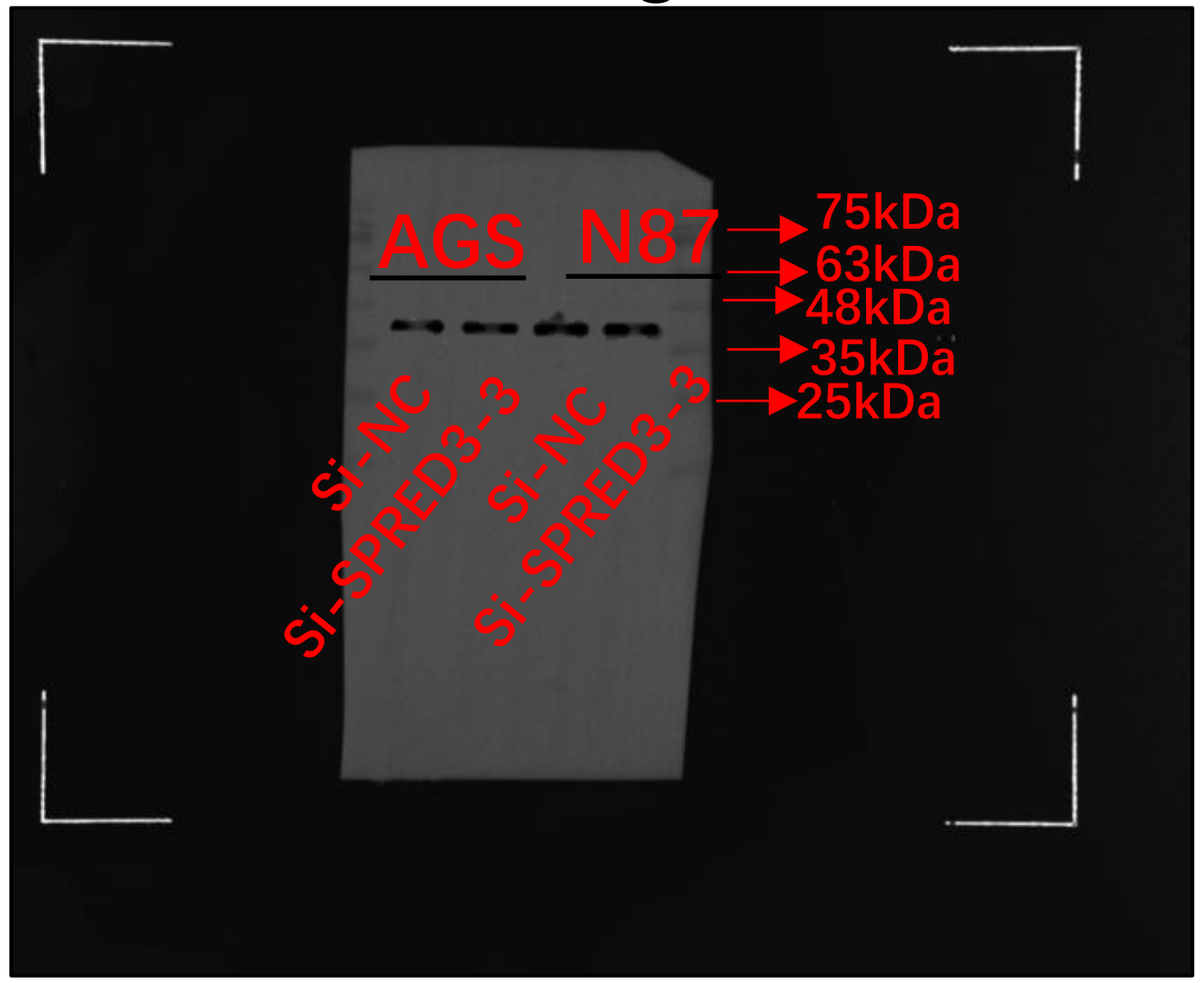

Multiple exposure images

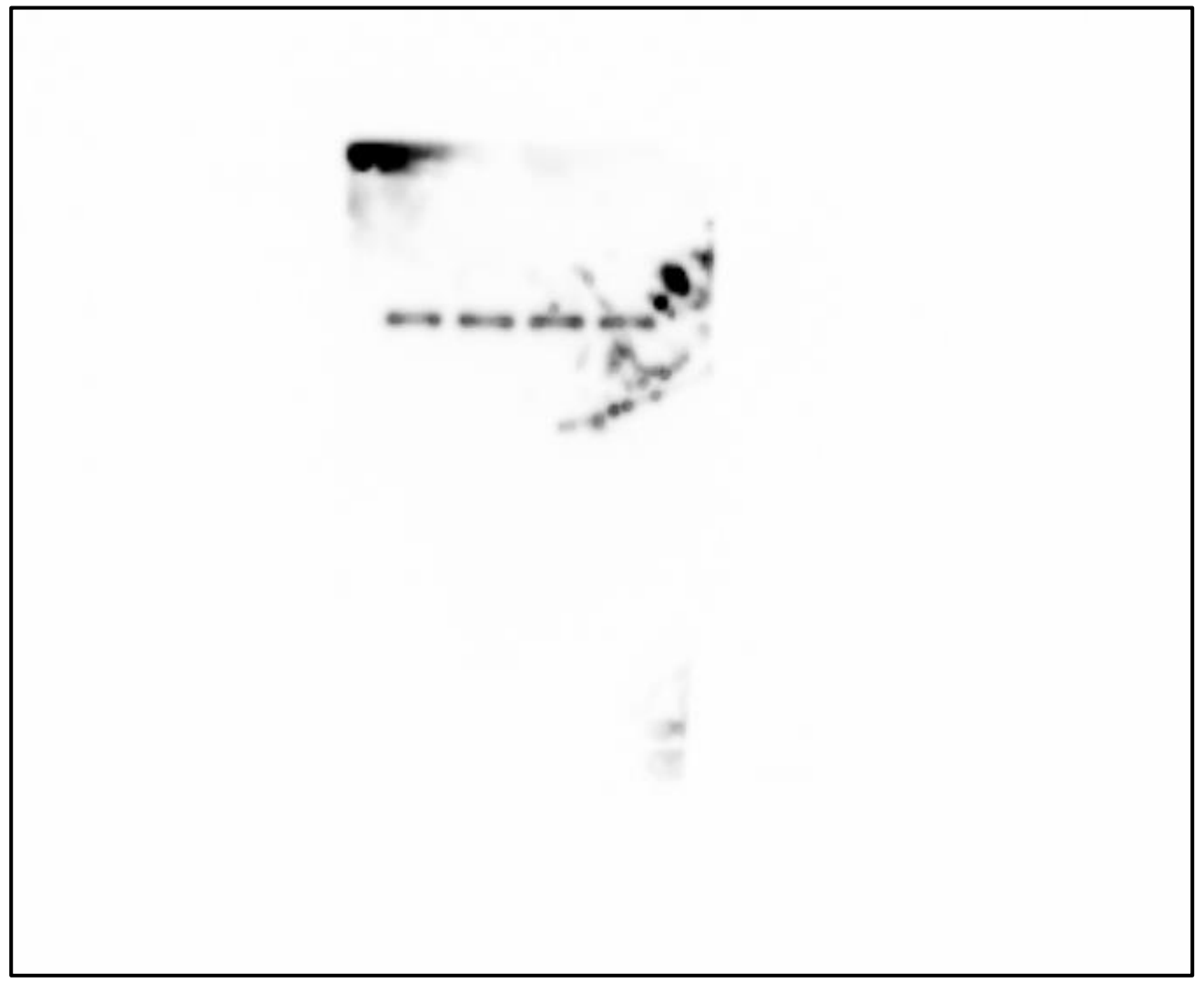

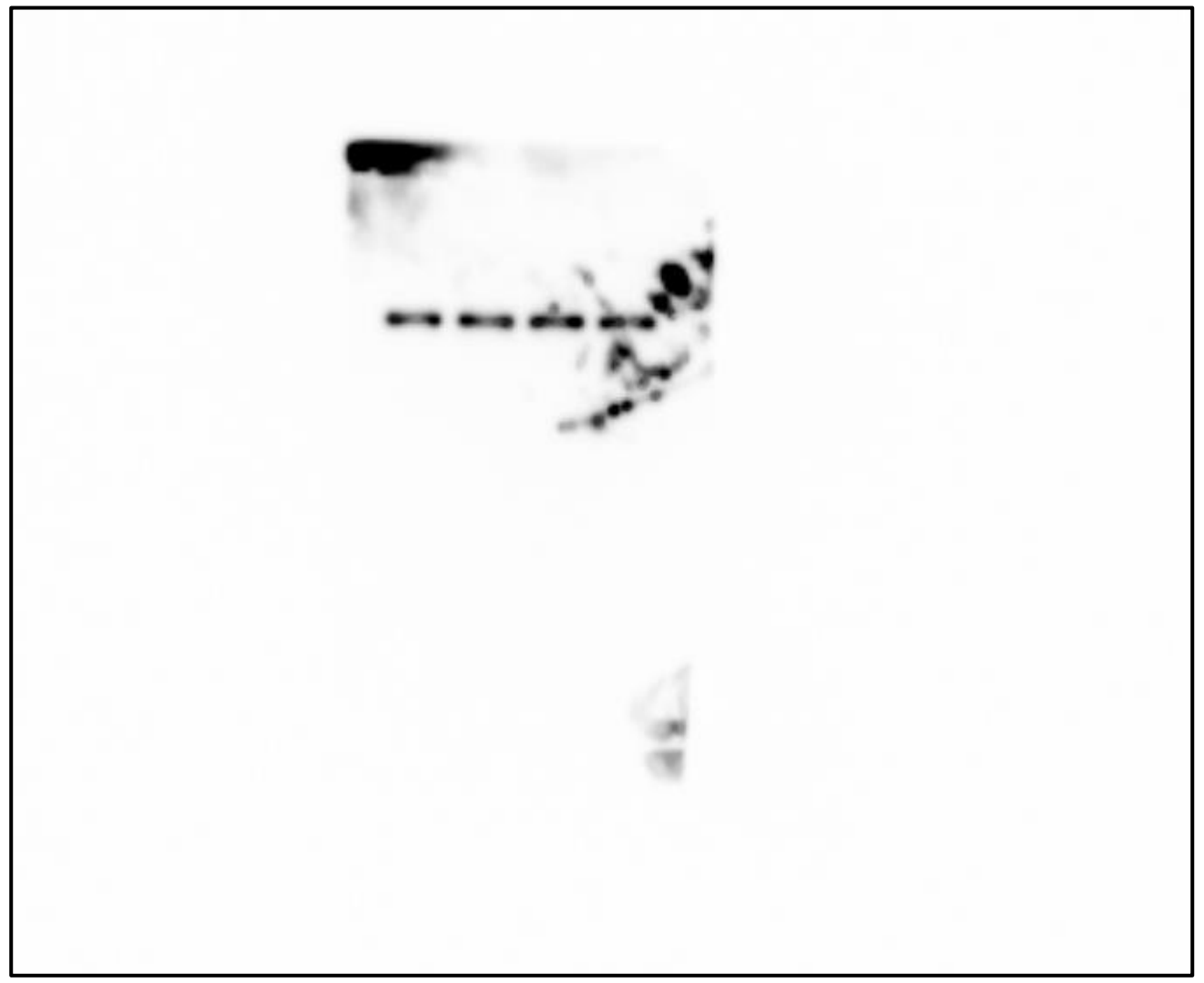

The primary images of the blots-Figure 5F—p-MKK3/6

AGS and N87 cell lines

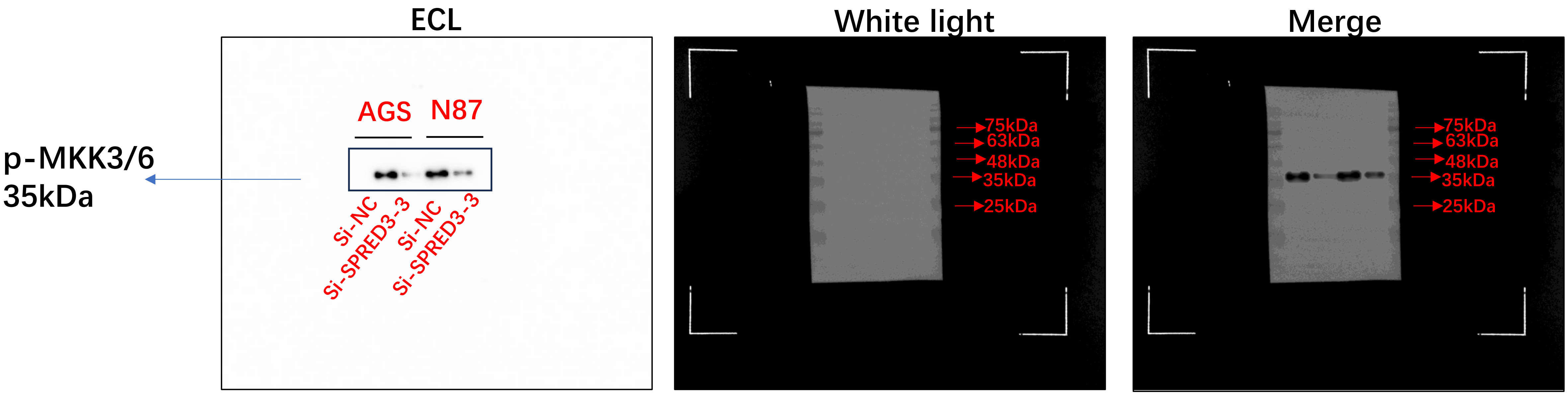

Multiple exposure images

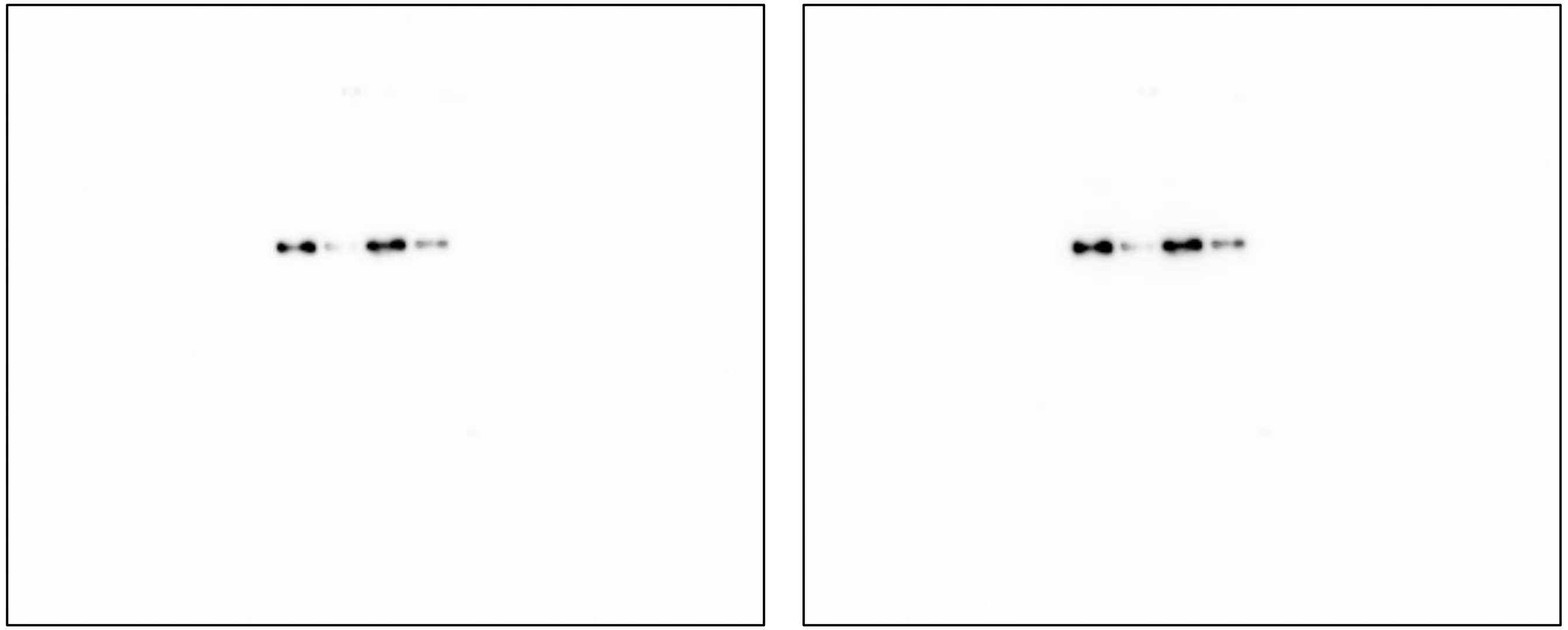

The primary images of the blots-Figure 5F—IκBα

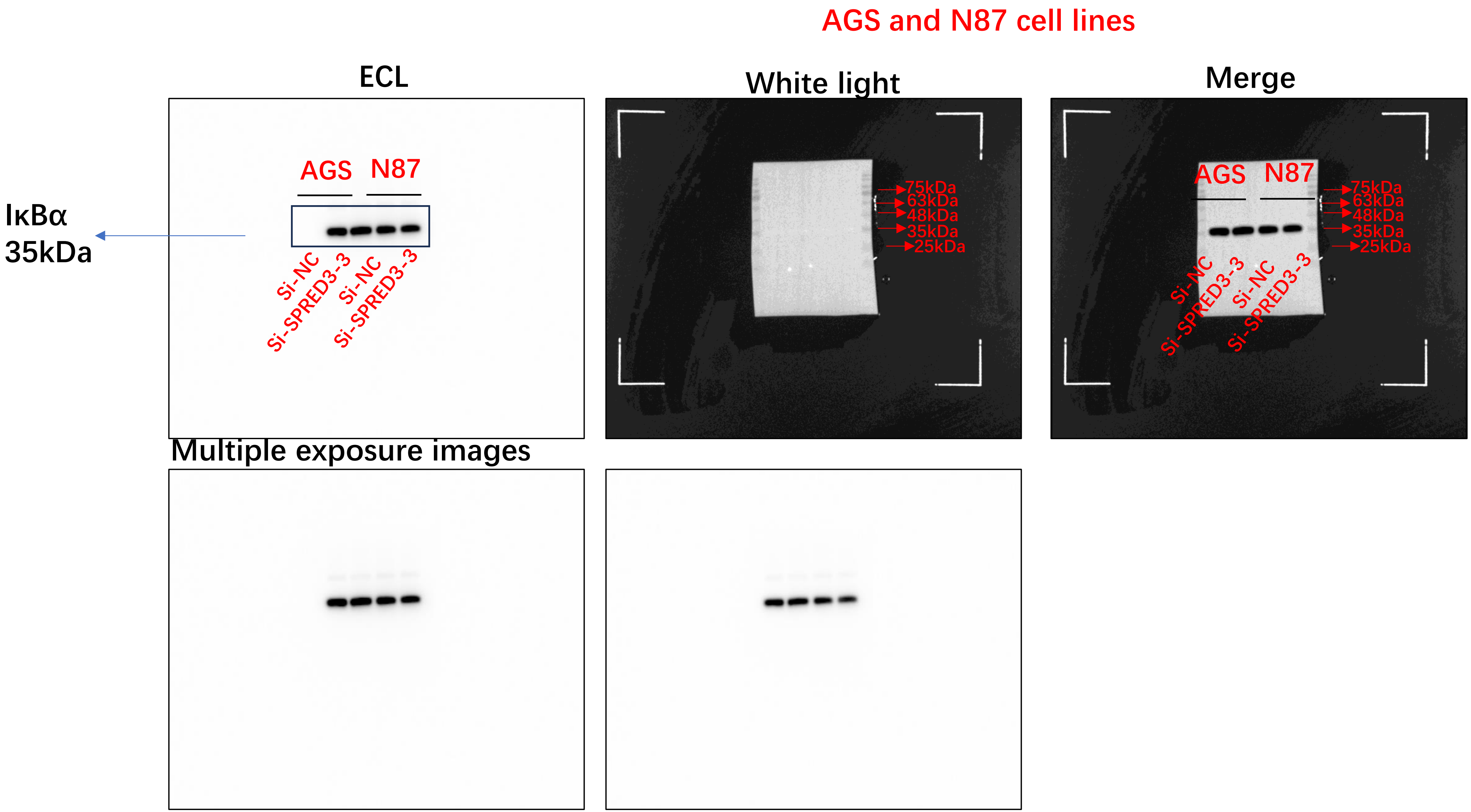

The primary images of the blots-Figure 5F—p-IκBα

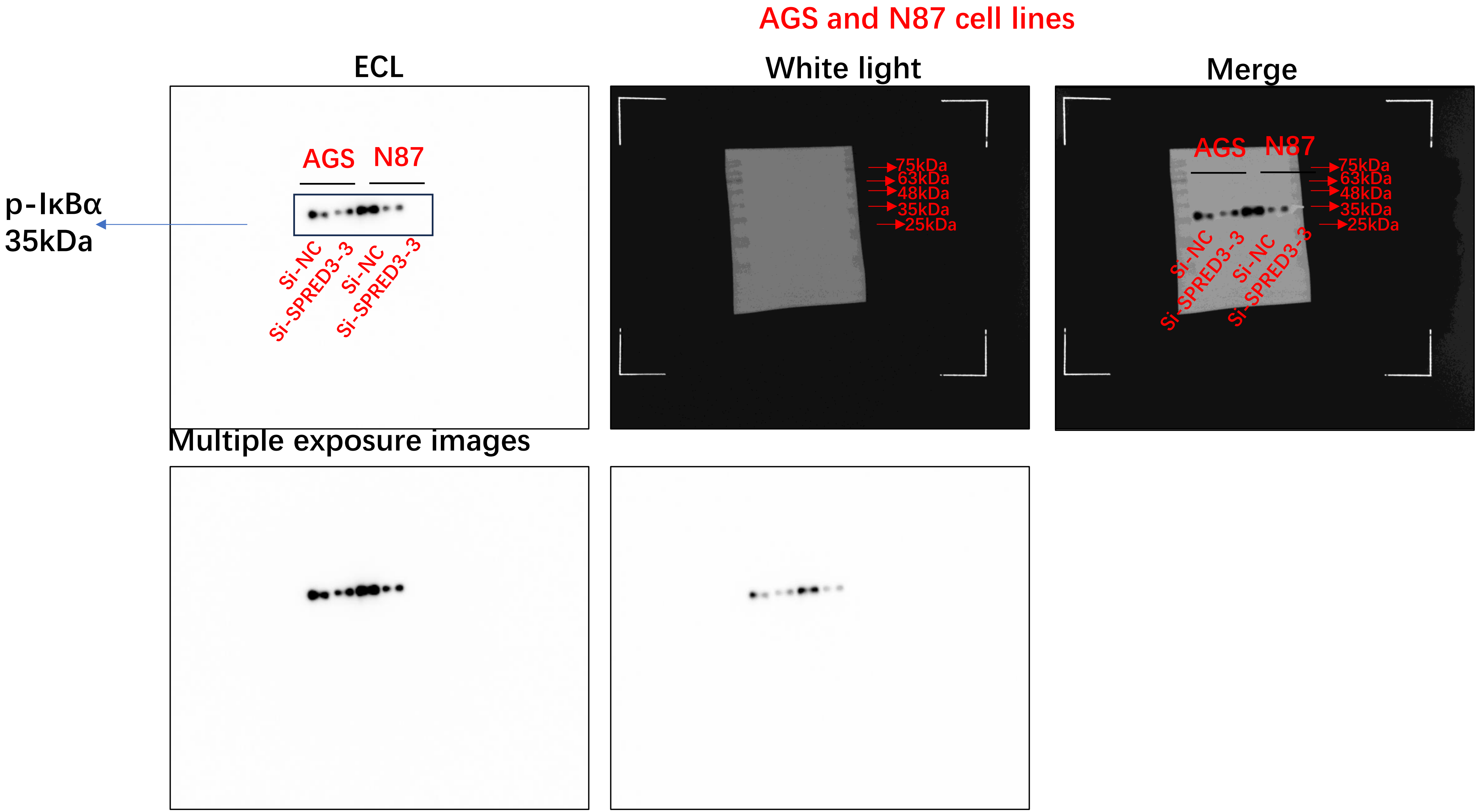

The primary images of the blots-Figure 8D--Alpelisib

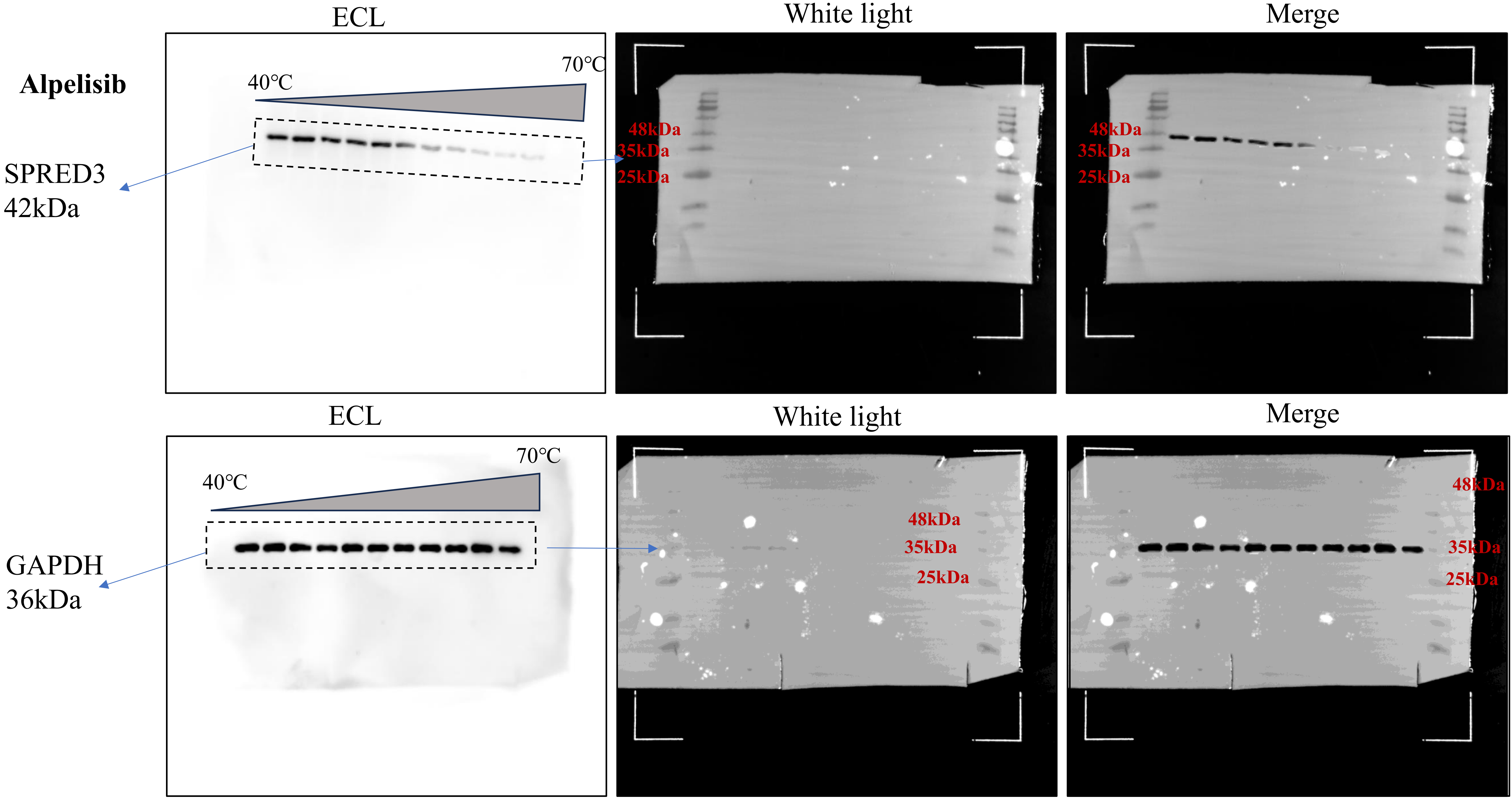

The primary images of the blots-Figure 8D--DMSO

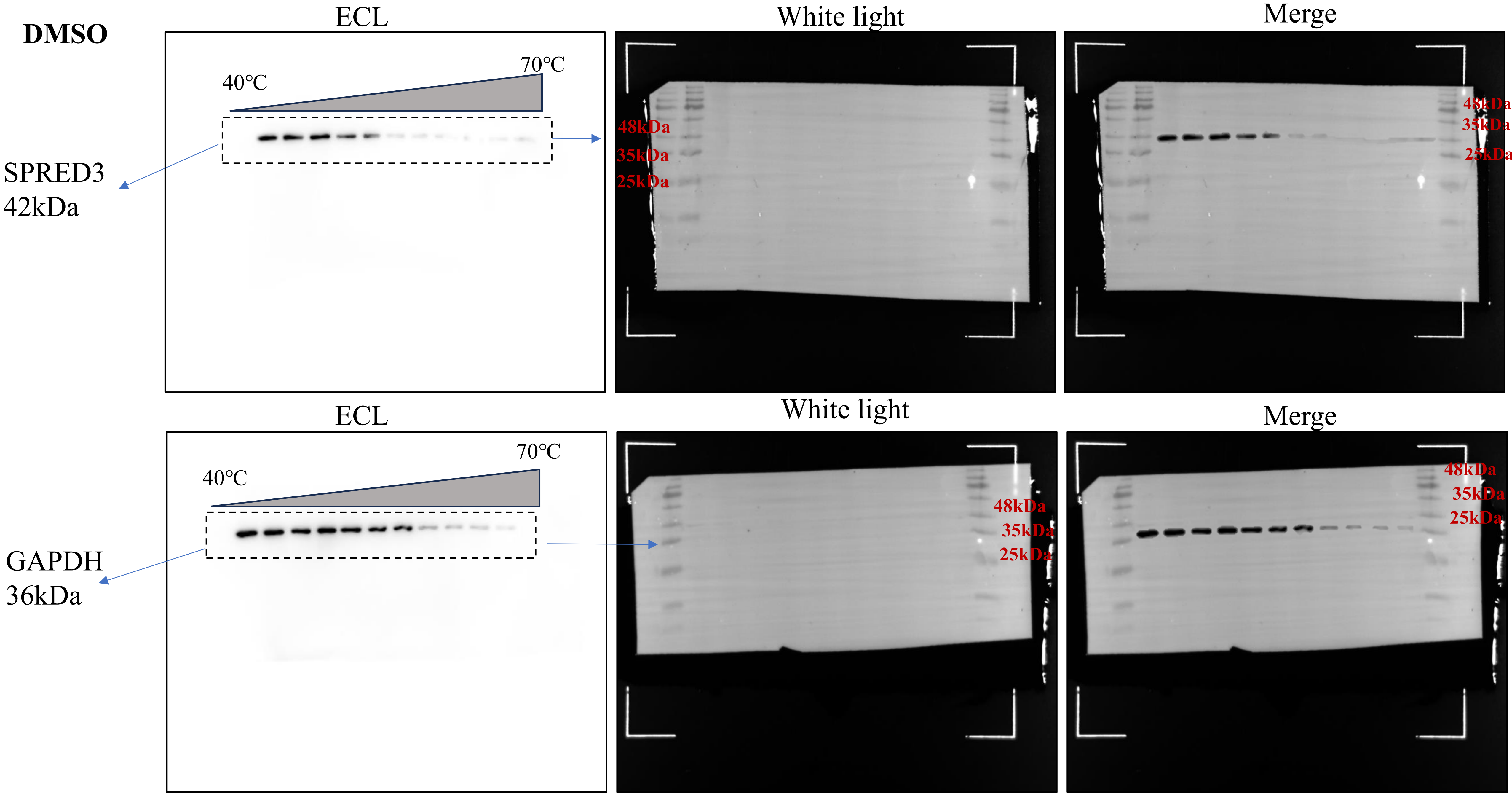

The primary images of the blots-Figure 8F--GAPDH

AGS + DMSO

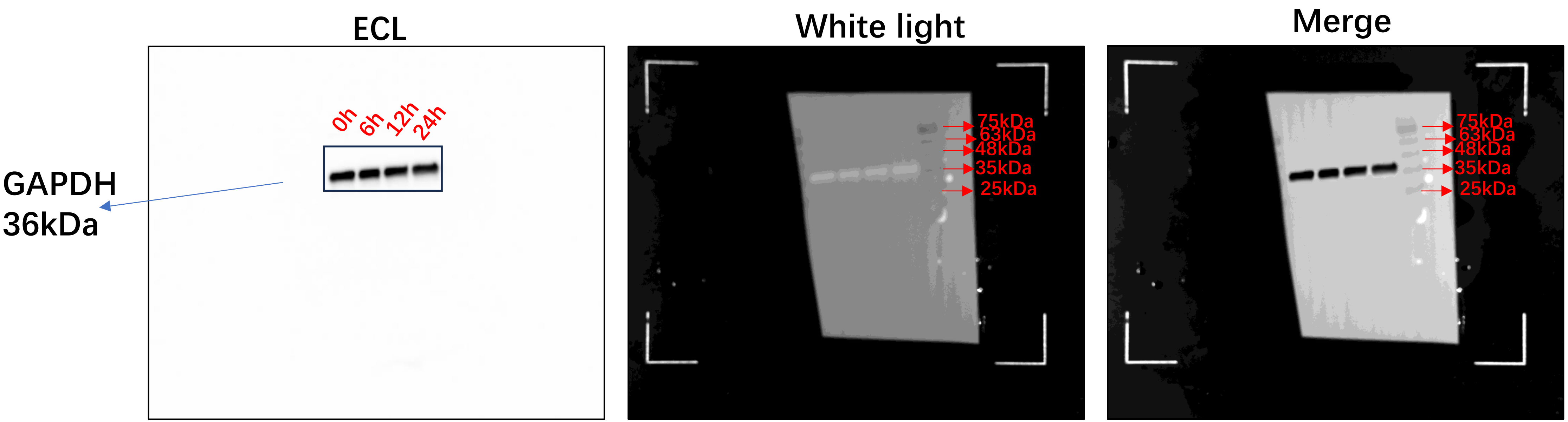

Multiple exposure images

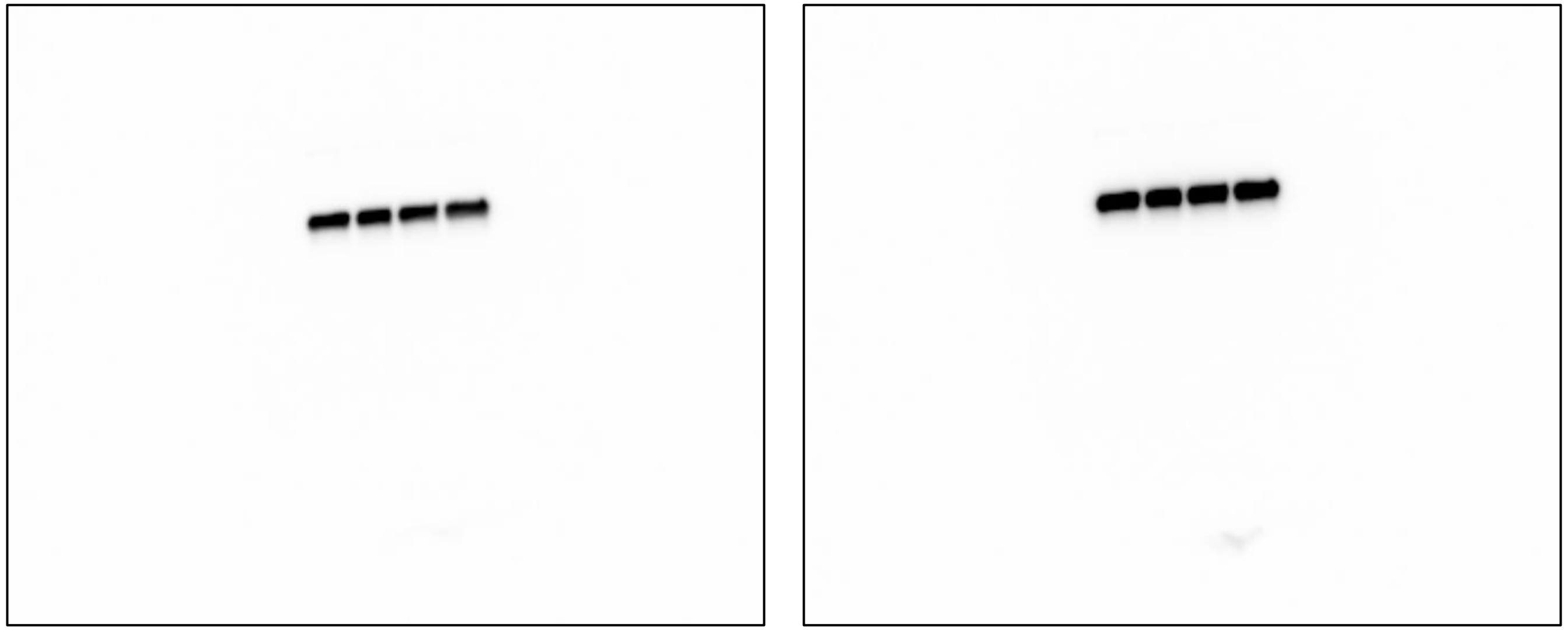

The primary images of the blots-Figure 8F—SPRED3

AGS + DMSO

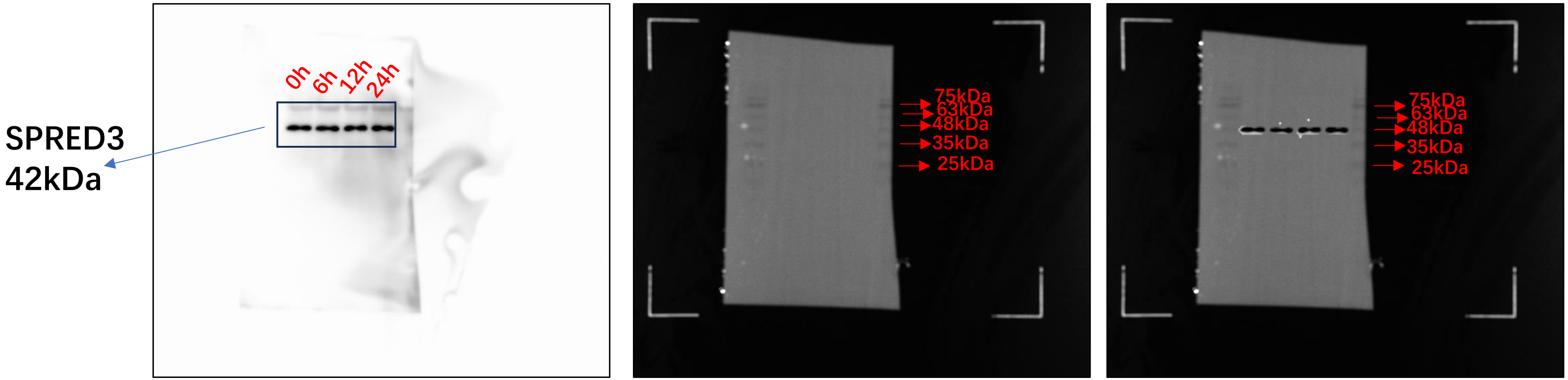

Multiple exposure images

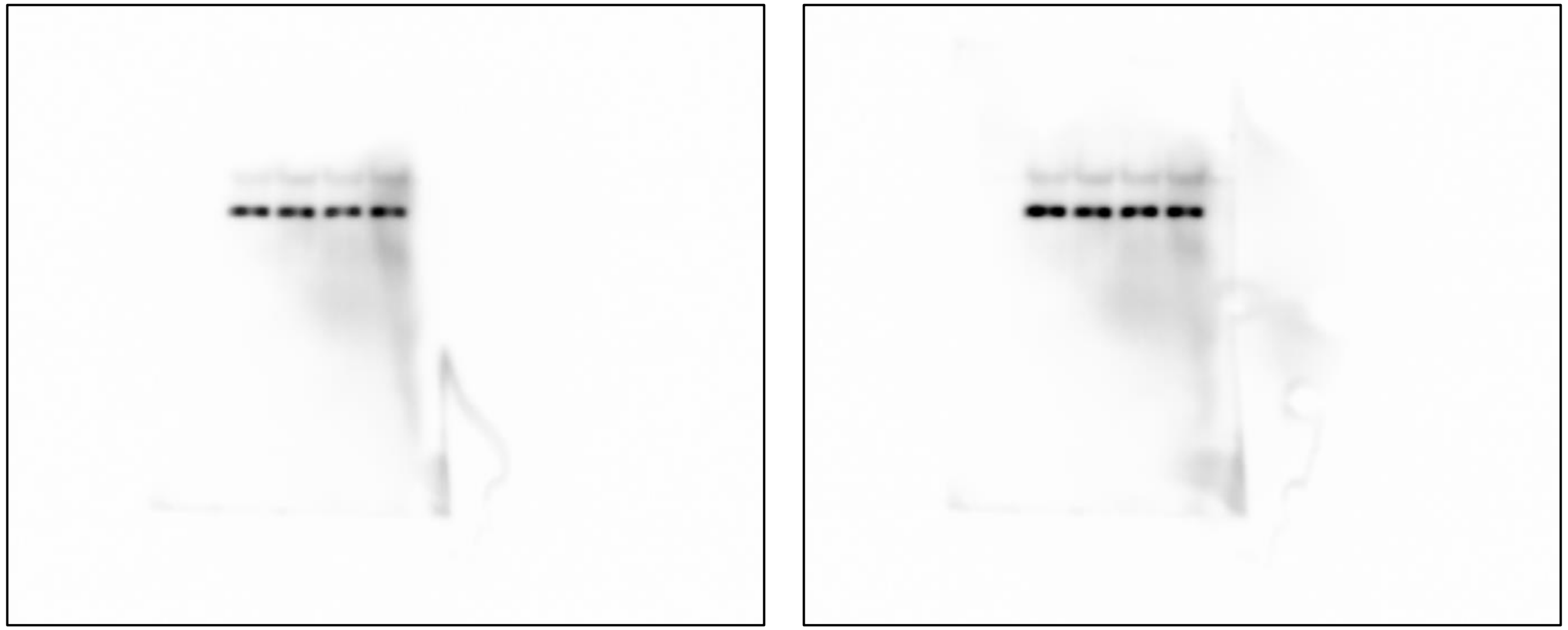

The primary images of the blots-Figure 8F--GAPDH

AGS + Alpelisib

ECL | White light | Merge

GAPDH
36kDa

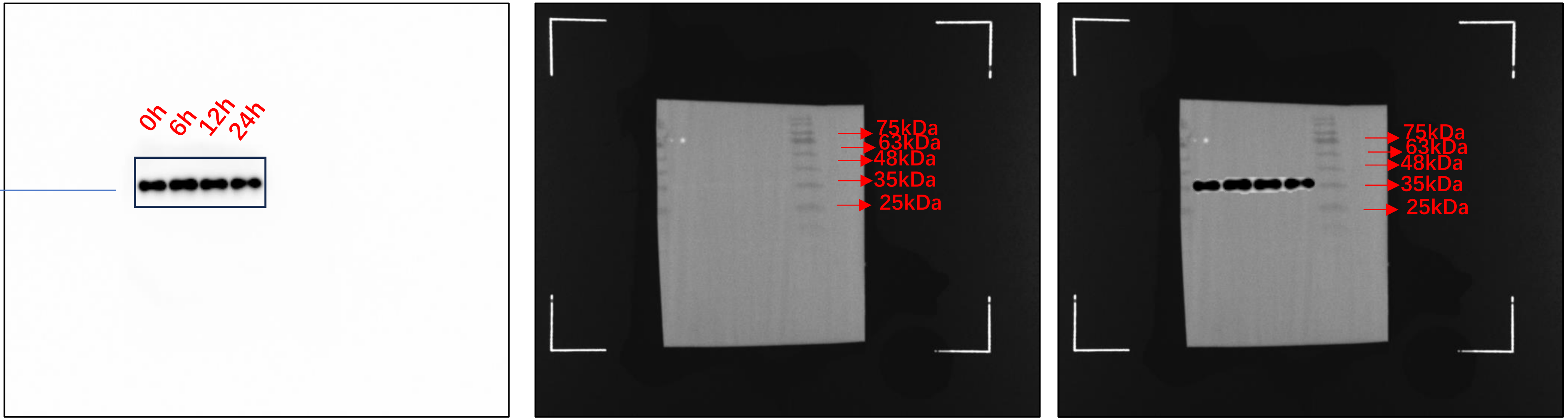

Multiple exposure images

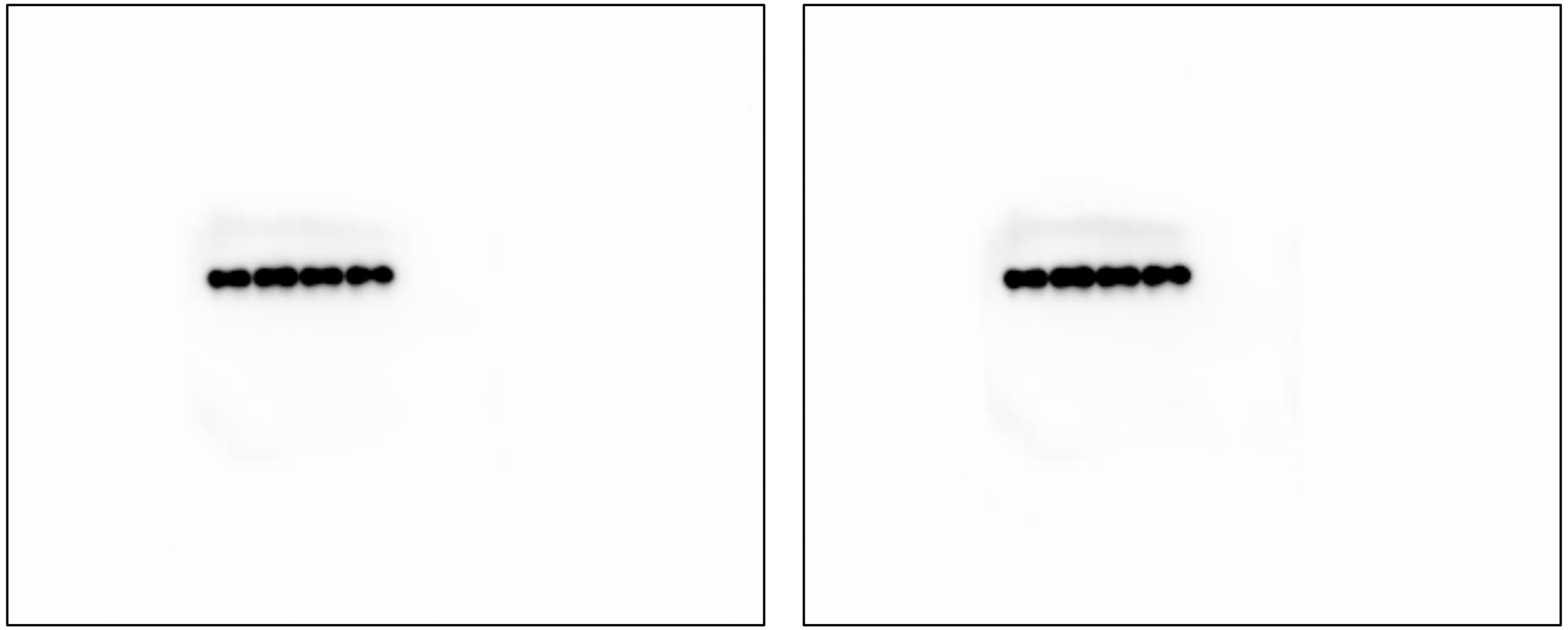

The primary images of the blots-Figure 8F—SPRED3

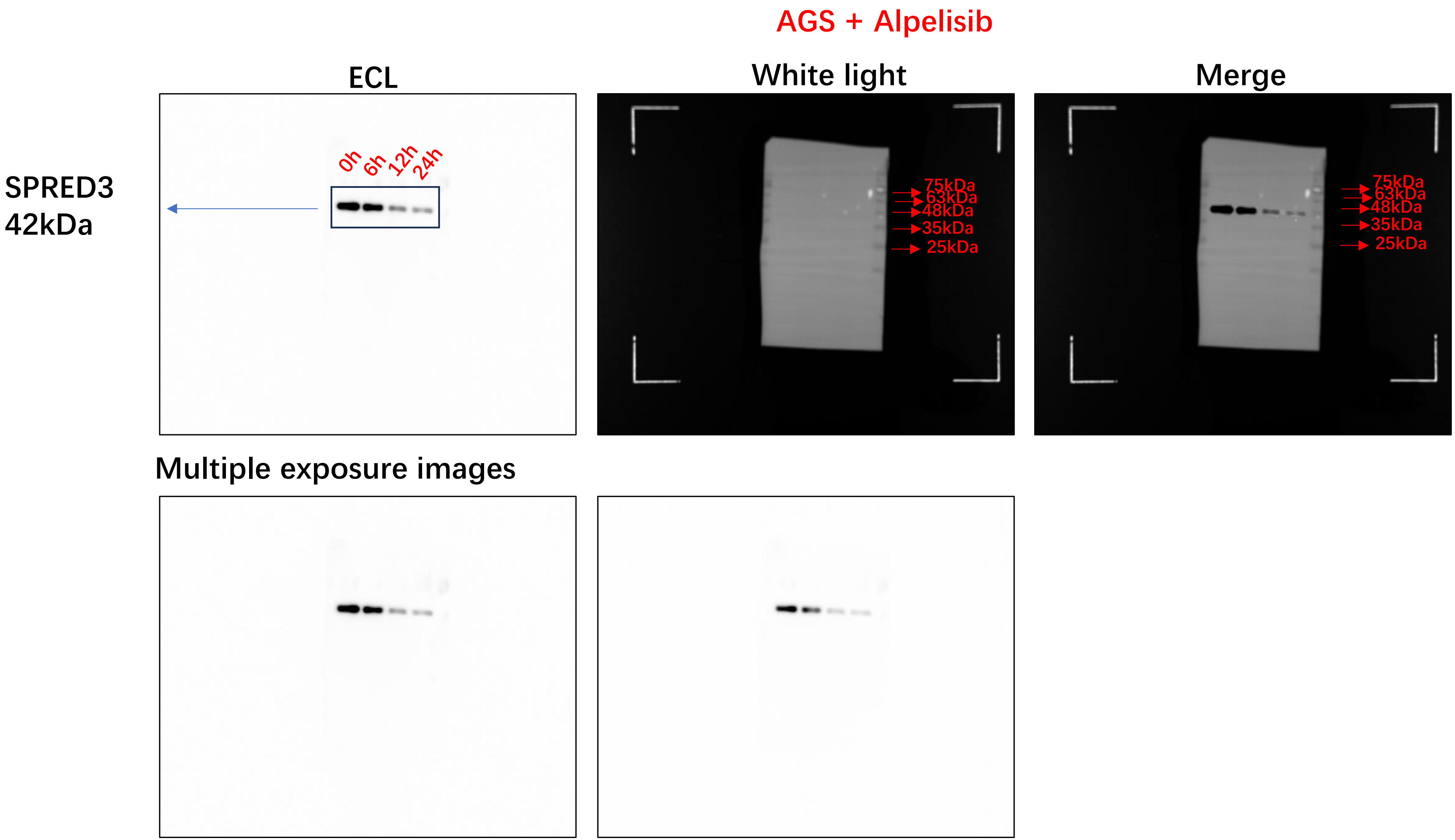

Supplement: Supplementary file 1 [file Image1.pdf]
